# AutoTransOP: translating omics signatures without orthologue requirements using deep learning

**DOI:** 10.1038/s41540-024-00341-9

**Published:** 2024-01-29

**Authors:** Nikolaos Meimetis, Krista M. Pullen, Daniel Y. Zhu, Avlant Nilsson, Trong Nghia Hoang, Sara Magliacane, Douglas A. Lauffenburger

**Affiliations:** 1 Department of Biological Engineering, Massachusetts Institute of Technology, Cambridge, MA 02139 USA; 2 Department of Biology and Biological Engineering, Chalmers University of Technology, Gothenburg, SE 41296 Sweden; 3 School of Electrical Engineering and Computer Science, Washington State University, Pullman, WA 99164-236 USA; 4 Institute of Informatics, University of Amsterdam, Amsterdam, The Netherlands; 5 MIT-IBM Watson AI Lab, Cambridge, MA 02139 USA

**Keywords:** Computational biology and bioinformatics, Computer modelling

## Abstract

The development of therapeutics and vaccines for human diseases requires a systematic understanding of human biology. Although animal and in vitro culture models can elucidate some disease mechanisms, they typically fail to adequately recapitulate human biology as evidenced by the predominant likelihood of clinical trial failure. To address this problem, we developed AutoTransOP, a neural network autoencoder framework, to map omics profiles from designated species or cellular contexts into a global latent space, from which germane information for different contexts can be identified without the typically imposed requirement of matched orthologues. This approach was found in general to perform at least as well as current alternative methods in identifying animal/culture-specific molecular features predictive of other contexts—most importantly without requiring homology matching. For an especially challenging test case, we successfully applied our framework to a set of inter-species vaccine serology studies, where 1-to-1 mapping between human and non-human primate features does not exist.

## Introduction

Animal and cellular models are essential tools for studying the underlying biology of human diseases, but these insights are not always clinically translatable, resulting in the failure of numerous therapeutics in clinical trials^1,2^. A common approach is to choose orthologous biomolecules, including genes, proteins, and cellular pathways, to perform direct functional comparisons across species. However, functional divergence and the absence of orthologous biomarkers can hinder these direct comparisons between species^3–5^. Furthermore, within the same species, the transcriptional response to chemical stimuli can be cell type-specific due to distinct genetic profiles, creating an additional barrier to understanding the mechanism of action of therapeutics^6–9^. Consequently, computational systems-based approaches are needed to gain a better understanding of the relationship between biological models and translate information gained from different model systems.

Advancements in sequencing technologies have enabled the generation of large-scale datasets from both animal and human species, facilitating more powerful analyses and comparisons of molecular features between different biological systems^2,3,10–13^. This has led to the development of numerous new statistical and machine learning models^3,13–17^ for identifying similarities between species and experimental models. Notably, most existing approaches focus on direct correlations between analogous biomarkers or processes across species despite known species and model system differences. In an attempt to address this challenge, Brubaker et al. proposed a technique called “Translatable Components Regression”^18^ (TransCompR), which maps human data into the principal component space of data from another species to identify translatable animal features that can predict human disease processes and phenotypes. Although this approach has been successfully applied to gain insights into some inflammatory pathologies^18,19^, it depends on homologs or comparable molecular features between species. While TransCompR is well suited to identify omics signatures in one species that is most germane for understanding phenotype characteristics in another, it is not centrally designed to integrate signatures across species. Moreover, this approach is by design only capable of deciphering linear relationships, thus potentially excluding non-linear biological relationships.

With the advent of deep learning, particularly autoencoders, there is great potential to develop approaches that can approximate the non-linear relationships underlying different biological systems and species. Autoencoders are artificial neural networks (ANNs) that can embed raw input data into a lower dimensional space from which the original data can be reconstructed^20^. Autoencoders have been used in several tasks in biology, including analyzing high dimensional data^21,22^, denoising single-cell RNA sequencing data^23–25^, deciphering the hierarchical structure of transcriptomic profiles^26,27^, and predicting gene expression caused by various stimuli^28–30^. One such model, DeepCellState^31^, focused on translating cellular states, can predict the transcriptional profile of a cell type after drug treatment based on the behavior of another cell type. However, similar to TransCompR, this approach depends on a 1-1 mapping of molecular features between cells to capture a global cell representation. Another recently proposed framework is the compositional perturbation autoencoder (CPA)^32^. It can construct a basal latent space devoid of covariate and perturbation-specific signals, capturing only the basal cell state in single-cell RNA sequence data. CPA can be used to generate *in-silico* transcriptional profiles at the single-cell level for different perturbations, cells, and species, although it still requires the mapping of orthologous genes. To overcome such limitations, an approach similar to those used in language translation autoencoder-based models, which create a global language representation^33,34^, may be useful and could aid biological inter-systems translation when 1-1 mappings between features do not exist.

In this study, we use ideas from language translation models^33,34^ and incorporate elements of the CPA approach to develop an ANN framework hence referred to as AutoTransOP, Autoencoders for Translating Omics Profiles, which utilizes separate autoencoders for each biological system, enabling the mapping of samples into a global cross-model space, while providing feature importance estimates for various phenotype-prediction tasks. It is important to note that the globality of the latent space is not the goal itself, but it serves as a way to achieve better performance in translating omics profiles. The basic model is trained to simultaneously minimize the reconstruction error of the input and the distance between samples coming from the same condition in the global latent space. Our framework is benchmarked, using the latest version of the L1000 dataset^12^, against the established approaches of TransCompR^18^, FIT^15^, and the ANN approach of DeepCellState^31^, which all require 1-1 feature mapping. We demonstrate that our approach outperforms FIT and DeepCellState, while there is no difference when comparing with TransCompR in cellular models. Additionally, we present several variations of the model and we illustrate the adaptability of our framework by applying it to data of varying omics type and sample size to answer different biological questions of interest. Furthermore, we demonstrate its biological interpretability, an aspect that deep learning models often struggle to attain, by using an integrated gradients approach^35^. To analyze the performance of the model in inter-species translation we performed mouse^36^ to human^37^ translation of single-cell transcriptomics of lung fibrosis, as well as non-human primate^38,39^ to human translation^40^ of smaller-scale serology datasets to predict HIV vaccine efficacy in humans. The latter serves as a case study of cross-species translation where no 1–1 mapping between features exists. It is worth noting that all three examples are different use cases where different models are trained separately, and in this study, different data modalities (e.g., bulk of single cell) are never combined in one model. After building the model, we identified serological features in non-human primates that are predictive of protection against HIV in humans, without analogous features necessarily being present in human data. These findings demonstrate that features derived from this approach can be predictive of the phenotypic profile of another biological model without requiring them to be homologs, allowing us to maximize the amount of information we can capture from different model systems to advance our understanding of complex human disease biology.

## Results

### A flexible framework for omics translation

**Fig. 1:**
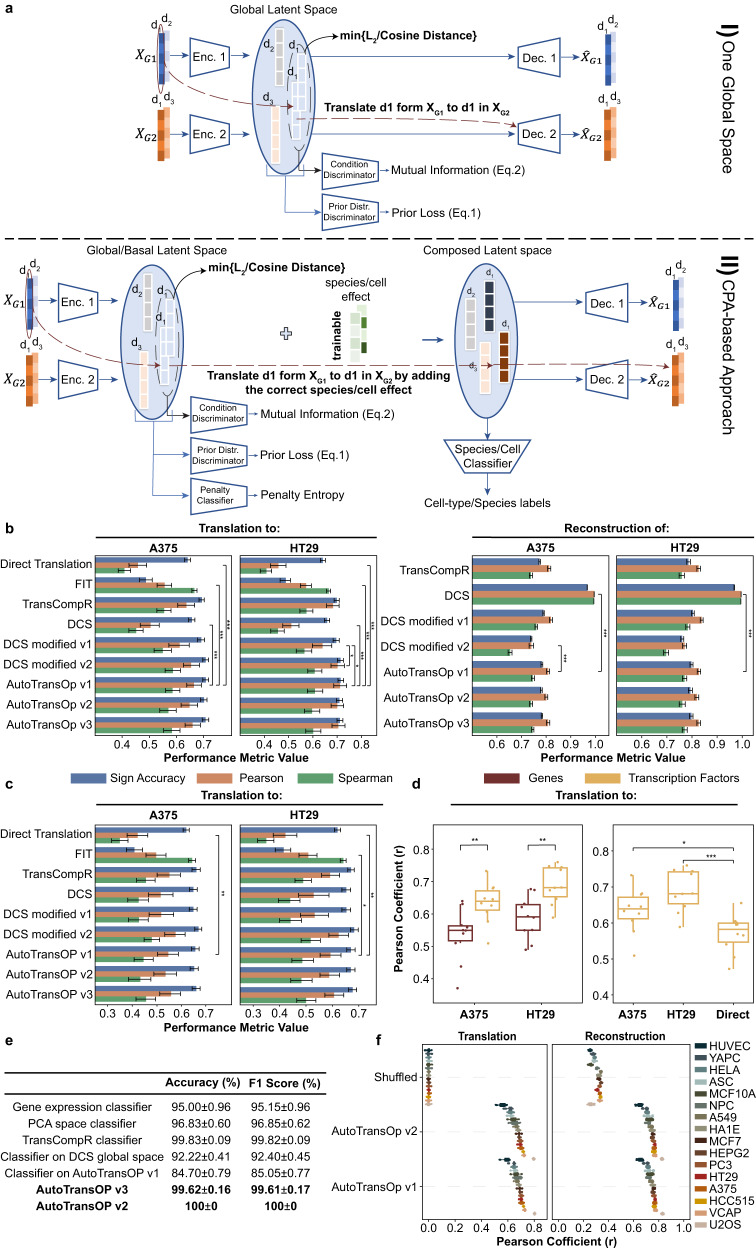
Model architecture and basic performance metrics. **a** Framework architecture main variations: I) *AutoTransOP v1*: One global space is constructed by mapping omic profiles in a space where the distance between embeddings coming from the same perturbation is minimized. II) *AutoTransOP v2*: Architecture combined with the CPA approach, where the latent space is separated into two, one global devoid of species/cell effect and a composed latent space. d_i_ signifies “drug perturbation i” and the illustrated vector corresponds to the vector of drug-induced gene expression values. **b** Model performance in reconstructing and translating gene expression profiles between the two cell lines with the most common perturbations in the L1000 dataset, A375 and HT29, by using only the 978 measured landmark genes. *AutoTransOP v3* is the one with a classifier simultaneously trained in one global latent space. For DCS modified v1–v2, see the corresponding methods sections. It is worth noting that DCS modified v2 has a distance term and a direct translation term in its training loss. **c** Model performance in reconstructing and translating gene expression profiles between A375 and HT29 by using all 10,086 genes that are either measured or belong to those that are well-inferred computationally. **d** Performance in inferring transcription factor activity by using the translated/predicted gene expression. **e** Performance in correctly classifying cell lines in different cases. Reported values are the mean ± standard error (SE). **f** Performance by using different inputs in the L1000. For all comparisons in this figure, a two-sided Wilcoxon test was used with *n* = 10 per group. The error bars in the bar plots (**b**, **c**) denote 95% Confidence Intervals (CI). In all boxplots, the centerline denotes the median, the bounds of the box denote the 1st and 3rd quantiles, and the whiskers denote points not being further from the median than 1.5 × interquartile range (IQR).

We developed a flexible artificial neural network framework (see methods) for omics translation across biological models. It consists of separate ANN encoders and decoders for each biological system, e.g., cell line or species, that share a global latent space (Fig. 1a), eliminating the need for a 1-1 mapping between the features between systems. The primary goal of the framework is not the construction of a latent space that captures all the information of the input signature, as in most autoencoder-based approaches, but, similarly to language translation tasks, to build a global space that captures mostly information about conditions and stimuli, while filtering out as much as possible system-specific information to enable translation of perturbations. This is achieved by minimizing the distance of embeddings coming from the same condition (drug + dose + time point) and also maximizing their mutual information, which we later empirically estimated to validate the success of this task (Supplementary Fig. 3). The model’s input are samples described by a vector of genes containing their expression values. We implement two main variations of the global latent space intending to remove the system-specific effect of perturbations. The first variation of the framework (AutoTransOP v1) consists of a single global latent space that is created by maximizing the similarity of embeddings derived from the same condition/perturbation in a different species or cell line. The second variation (AutoTransOP v2) incorporates the idea of the recently published compositional perturbation autoencoder (CPA)^32^, where there are two separate latent spaces: (1) a global/basal latent space and (2) a composed latent space. The global latent space expands on the first variation with an additional discriminator that attempts to remove the cell-line or species effect by penalizing models where the classifier can detect from which encoder the latent representation originates^32^. In the composed latent space, a cell/species classifier is simultaneously trained to ensure there is a cell/species effect, which is either added through a trainable covariate vector^32^ or can also be added through two intermediate ANNs, allowing for non-linearity (see example in Supplementary Fig. 4). We utilize integrated gradients^35^ to estimate feature importance for various predictive tasks. Lastly, we also introduce a variation (AutoTransOP v3), with one single global latent space, where a classifier is simultaneously trained on the global latent space (see methods). This is a contradictory learning task where the framework attempts to simultaneously remove the cell line or species effect globally but also hides cell or species information in a few of the latent variables.

### Benchmarking reconstruction and translation of gene expression profiles between two cell lines

First, we compared our ANN framework with state-of-the-art techniques in the context of translating homologous genes between in-vitro models within the same species. We use the L1000 transcriptomics dataset^12^ to benchmark different approaches to translate the effects of perturbations between different human cell lines. AutoTransOP v1, AutoTransOP v2, and AutoTransOP v3 are compared with three previously published approaches, DeepCellState^31^, FIT^15^, and TransCompR^18^ (see methods for all). As a baseline, the models are also compared to “direct translation”, i.e., directly using the gene expression profile in one cell line as a prediction for the effect in another cell line. We evaluate the models both on the task of translating the gene expression profile between cell lines as well as the task of accurately reconstructing the gene expression for the same cell line. We evaluate them using several different metrics: i) Pearson’s correlation between predicted gene expression and actual gene expression, ii) the per sample Spearman’s rank correlation, and iii) the accuracy in correctly predicting the sign of drug-induced gene expression.

When utilizing the 978 landmark genes measured in the L1000, all of our framework’s variations provide a statistically significant increase in performance compared to the direct translation across all metrics (Fig. 1b, *p* values in Supplementary Table 1, Supplementary Table 2, Supplementary Table 3, Supplementary Table 4). When translating from the HT29 cell line to A375, AutoTransOP v1 outperformed FIT^15^ and the basic DeepCellState^31^ (DCS) methods. When translating in the reverse direction, from A375 to HT29, our framework also outperforms the different modifications of DCS (Fig. 1b). It can be noted that the 2nd modification of DCS that enforces similarity in the latent space like our model, also outperforms the basic DCS, which may support the importance of enforcing similarity in the global latent space via some distance metric. For reconstruction of the input within a single cell line, the basic DCS approach outperforms the other approaches, at the expense of its translation performance. On this metric, our approach performs well and comparably with the other methods (Fig. 1b). The alternative variations of our framework also perform comparably well.

When using the L1000 dataset with the computationally imputed expression of 10,086 genes, the performance of all approaches drops, though still better than the baseline. There are generally no statistically significant differences between variations of our approach and the other state-of-the-art approaches (Fig. 1c). To investigate the potential to later extend the method in cases where no 1-1 mapping exists, we trained models in 16 different cell lines, where one of the autoencoders in the framework is used for half of the landmark genes and the other for the rest. The goal is to create artificially different cell lines where the genes used are different with no 1-1 mapping. We selected the landmark genes because of their low correlation to each other (Supplementary Fig. 5), which was also reported in the L1000 study^12^. Half of the genes are selected randomly five times and the models are evaluated using 5-fold cross-validation, where 80% of the data are used for training and 20% for testing. Interestingly, our approach achieves very high performance (Fig. 1f), even up to ~0.8 correlation for the translation task in the U2OS cell line, significantly better than randomized models trained by randomly shuffling genes. This not only provides evidence for the potential of translating omic profiles in cases with no 1-1 mapping for their features but also demonstrates the potential to be used in gene imputation. Finally, in the case of the A375-HT29 cell line pair, the model again performs better than direct translation when using different genes as input for each cell line, e.g., using only the 978 landmark genes for the A375 cell line and all the 10,086 genes for HT29 (Supplementary Fig. 6), and it is comparable with the performance of models using the same genes.

### Performance in using predicted gene expression to infer transcription factor activity

While the performance was worse in predicting the full set of 10,086 imputed genes, we reasoned that these imputed transcriptomic profiles may still be useful as input into different aggregation methods, e.g., to infer the activity of transcription factors (TFs). When we inferred transcription factor activity (see methods), model performance increased relative to using all 10,086 genes and was comparable to that in the case of the landmark genes (Fig. 1b, d). Our model was not as successful at predicting gene set enrichment (Supplementary Fig. 7). Autoencoders have been previously shown to be capable of capturing regulatory relationships between genes^26,31^ but, to our knowledge, not gene set enrichment, which might explain why we observed increased performance only when inferring TF activity.

### Creating cell-line-specific regions in the latent space enables robust cell classification

It is important to evaluate whether the cell line or species effect is successfully added to the composed latent space and whether the framework can retrieve it. To establish the ability of the model to capture cell-line-specific information, we evaluated the performance in classifying the cell line when using all 10,086 genes of the L1000 dataset (Fig. 1e). The performance of ANN classifiers trained directly on the L1000 gene expression data serves as the baseline. Classifiers built with pre-trained embeddings from DCS or our framework with one global latent space, are expected to have lower performance than the baseline as these approaches generate embeddings aiming to filter the cell-line effect as much as possible. Our framework, which aims to actively try to find a shared latent space that is independent of the cell line information and hence only contains information common across cell lines, seems to be better at “forgetting” the cell line of origin in the global space than DCS, thus generating more global embeddings (Fig. 1e). Other evidence of the higher globality of AutoTransOP’s latent space can be found by interrogating in detail the distributions of the latent variables between the two cell lines.

Examining the univariate differences of each latent variable, between embeddings of perturbations from the A375 cell line and the HT29 cell line, yielded 590 latent variables (out of 1024) in the case of the DCS approach^31^ and 275 for our approach (Supplementary Fig. 8), which is less than half. This means that while there are still cell line differences in our case and the space is not completely global, it is more global than DCS. Additionally, we examined the effect size (using Cohen’s *d*) between the two cell line distributions of each latent variable, in the case of our approach and DCS, and we observed that Cohen’s d for DCS is higher and the difference is statistically significant (Supplementary Fig. 9), meaning that not only more latent variables are different between the 2 cell lines but also with a larger difference. Finally, we compared the distribution of cosine distances of pairs of embeddings coming from the same cell line, between DCS and our approach and it seems the embeddings from the same cell line are closer together in DCS (Supplementary Fig. 10), again indicating a less global latent space.

Interestingly, when simultaneously training a classifier in the global latent space we can outperform the baseline while the cell-line effect is still partially filtered in the higher dimensions (Supplementary Fig. 11). AutoTransOP v2, with the CPA-based separation of latent spaces, in the composed latent space classifies cell lines with 100% accuracy, even though the similarity of input gene expression data between training and test sets, as well as the latent space embedding similarity, is generally low (Supplementary Fig. 12). AutoTransOP v2 can create very well-separated cell-line-specific regions (Supplementary Fig. 13) in the composed latent space, indicating the framework’s ability to shape the latent spaces with robust cell-line-specific regions and explaining the observed accuracy. AutoTransOP v2 was chosen for further analysis, even though it is not the best variation of the model performance-wise because the difference in the L1000 benchmark is not statistically significant and AutoTransOP v2 creates the composed latent space, containing all the information about the original perturbation.

### Analysis of the framework’s dependence on different aspects of the data

**Fig. 2:**
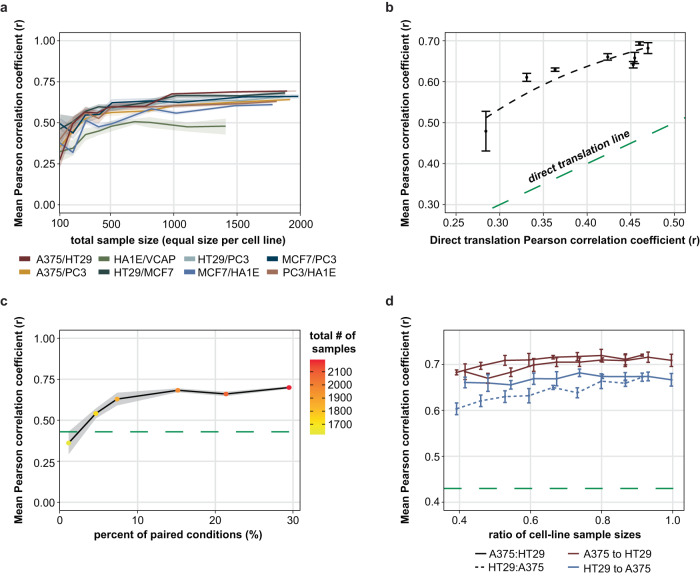
Analysis of framework’s performance and dependence on the data. **a** Performance in the translation task of AutoTransOP v2 (CPA-combined approach) across different cell-line pairs and different sizes of training data. **b** Model performance in translation as a function of the initial similarity of 2 cell lines. **c** Model performance in translation for different percentages of paired conditions. **d** Model performance in translation for low-to-medium cell-line imbalance in the conditions of the training samples. All error bars in this figure denote a deviation of one Standard Error (SE) from the mean. All the shaded areas in this figure represent a deviation of one SE from the mean.

We further investigated how the performance of the framework was influenced by different factors, focusing on the model incorporating elements of the CPA approach. The framework has similar behavior and performance across cell-line pairs (Fig. 2a). For all cell lines, where input is a vector of 978 genes, ~600 total training samples are sufficient to train a high-performance model. Some cell-line pairs perform slightly worse, as the original correlation between the same perturbations in the cell-line pair correlates with the model’s performance (Fig. 2b). Another important factor that requires investigation is the amount of paired conditions, meaning drug perturbations tested on both cell lines used to train a model, for the same dose and duration, which are used to enforce globality in the latent space, by minimizing the distance of the embeddings of such signatures in the latent space. On this front, we gradually increased the percentage of conditions that are paired in our training data, without a significant effect on the amount of data used to train the model. Interestingly, the amount of paired conditions required to successfully facilitate translation can be as low as ~10–15% of the samples being paired (Fig. 2c). Finally, it seems the model is not affected by a moderate imbalance in the number of conditions coming from each cell line (Fig. 2d). Similar trends are observed when using 10,086 genes (Supplementary Fig. 14).

### Evaluation of latent space embeddings

**Fig. 3:**
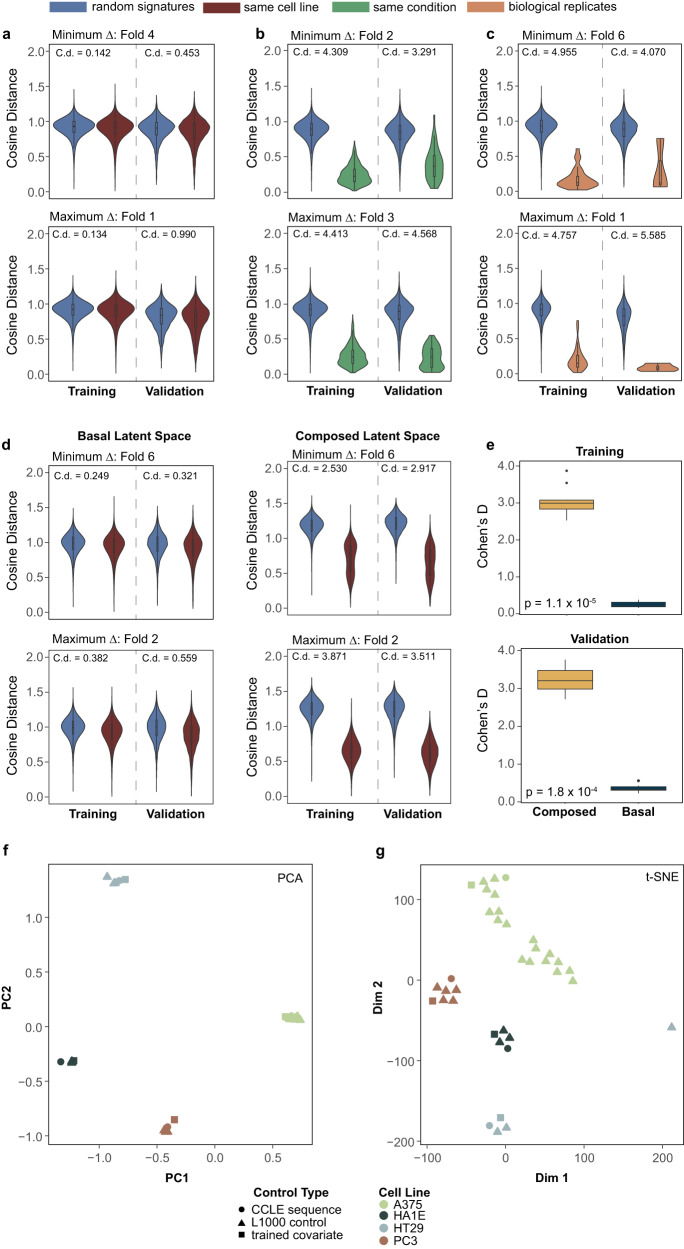
Properties of the latent space and model parameters interpretation. The two splits in tenfold cross-validation present each time here are the ones where the maximum and minimum difference between the two distributions is observed. For every other split, the difference is between these two extreme cases. Additionally, (**a**–**c**) come from *AutoTransOP v1*, with one global latent space, while the rest come from AutoTransOP v2. **a** Cosine distance between embeddings coming from random pairs of samples and pairs coming from the same cell line. **b** Cosine distance between embeddings coming from random pairs of samples and pairs coming from the same condition tested on a different cell line. **c** Cosine distance between embeddings coming from random pairs of samples and pairs being biological replicates. **d** Distance between embeddings coming from random pairs of samples and pairs coming from the same cell-line in the global and then the composed latent space in AutoTransOP v2. **e** Cohen’s *d* between distributions of cosine distances between random pairs of embeddings and embeddings coming from the same cell distribution. A two-sided Wilcoxon test was used with *n* = 10 per group. **f**, **g** 2D-Visualization of L1000 control conditions, untreated cell lines from the CCLE dataset, and the trainable vectors of AutoTransOP v2 containing the cell line basal effect added to perturbations. In all boxplots, the centerline denotes the median, the bounds of the box denote the 1st and 3rd quantiles, and the whiskers denote points not being further from the median than 1.5 × interquartile range (IQR).

A global latent space is expected to have several properties to be suitable for translation. We evaluate the embeddings produced from our framework based on three criteria (Fig. 3a–c): i) different cell lines should not occupy different subspaces, so embeddings of pairs coming from the same cell line should not be more similar to each other than embeddings from random pairs of samples (meaning randomly selecting two drug perturbation and calculating the distance of their embeddings), ii) pairs of embeddings coming from the same condition, regardless of cell line, should be similar, and iii) biological replicates should give similar embeddings, so pairs of embeddings from biological replicates should be similar to each other. We evaluated these criteria using the cosine distance in latent space. Only a small cell-line effect is observed in the global latent space, both for training and test embeddings (Fig. 3a, Supplementary Fig. 15). Embeddings coming from the same condition are closer to each other than embeddings coming from random pairs (Fig. 3b), while biological replicates are even closer (Fig. 3c), validating that indeed we have successfully constructed a stimuli-specific global latent space. Similar patterns can be observed in the global latent space when using the approach combined with elements of CPA (Fig. 3d), but with a cell-line effect visible in the composed latent space, as expected with this method. We use Cohen’s d to quantify the difference between the distributions of cosine distances across all folds in 10-fold cross-validation (Fig. 3e), proving that indeed, there is a much higher cell-line effect in the latent space than the effect in the global latent space.

### Interpreting the biological information captured in the parameters

Deep learning models are often criticized for their lack of interpretability, so we investigate the biological information captured by some of the model’s parameters. Since only the cell-line effect is minimized in the global latent space of the AutoTransOP v2, the trainable covariate (covariates such as species, cell type, etc.) vectors should only add a cell-specific effect. Intuitively, the global latent embeddings are expected to capture a “zero”/basal cell state corresponding to the expression of untreated cells (controls), and thus the trained covariate which is added to that global representation should be similar to the composed latent space vectors which now captures the cell line effect. To investigate this, we used control samples from the L1000 dataset not seen by the model during training, as well as samples coming from untreated cell lines from the Cancer Cell Line Encyclopedia^41^ (CCLE), using only the genes included in the L1000 landmark genes. Additionally, for this investigation two models were trained completely separately: the original benchmark model of A375/HT29 cell lines and another model using the PC3 prostate cancer cell line and the HA1E normal epithelial cell line. The latter pair was chosen because of high model performance (Fig. 2a) and because these two cell lines are significantly different in terms of biology. Each trained covariate, even though the models were trained separately, is observed to be closer to its respective cell-line control signatures, both when using PCA for dimensionality reduction (Fig. 3f), where clearly defined cell-line specific regions are observed, as well as when using t-SNE (Fig. 3g). This demonstrates that some parts of the model are biologically interpretable and capture specific information.

### Identification of features that are important for translation and cell classification

**Fig. 4:**
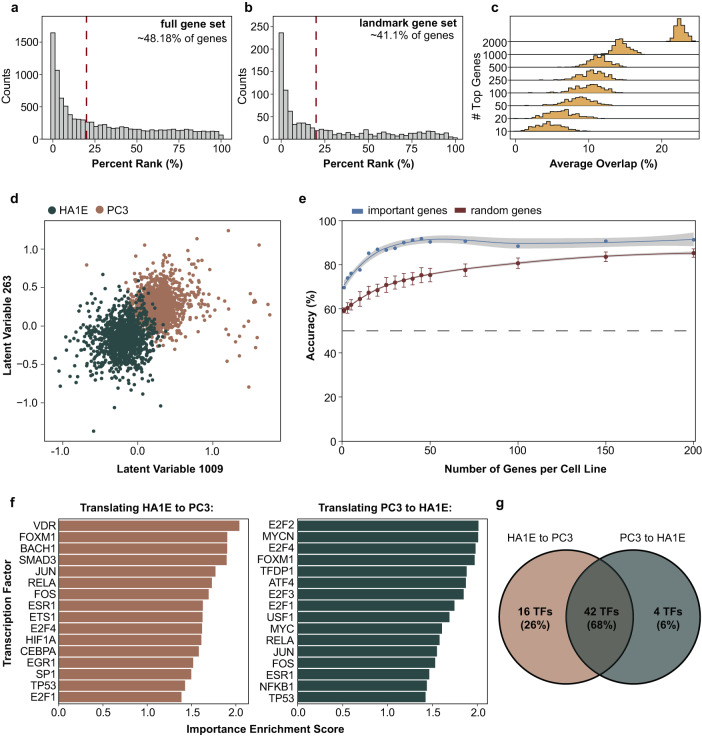
Feature importance investigation. **a**, **b** Distribution of percentage rank in terms of the importance of a gene to translate its expression to itself, using the 10,086 genes and the 978 landmark genes, respectively in the L1000 dataset. **c** Distribution of average overlap per sample, between top important and top regulated genes. **d** Separation of cell lines based on the top 2 most important latent variables according to the classifier. **e** Generalized Linear model (GLM) classification performance by using increasingly more important genes. The error bars denote the deviation of one Standard Error (SE) from the mean, and the shaded areas denote 95% Confidence Intervals (CI). **f** Top significantly enriched TFs, based on importance scores of genes, to translate between cell lines. The enrichment means that a lot of the genes that a TF controls are found to be important, thus these TFs might be important for translating a cell line to another. **g** Overlap of important TFs, derived from both directions of translation.

The framework can be used to identify latent variables and genes that can be of biological importance. As a case study, we selected the model of the PC3 and HA1E cell lines with a classifier trained simultaneously to classify the cell lines from which the samples were derived (contradictory learning tasks). To identify the importance of genes according to the model for a variety of tasks with respect to their output, an integrated gradient-based approach^35^ was utilized (Methods) that attributes an importance score to each variable of interest. Since the same genes are used for both cell lines, it can be interesting to identify which are important for the model to translate a gene expression profile from one cell line to another cell line. Interestingly, the model attributes more importance to many genes other than the gene of interest when translating across cell lines for the same condition (Fig. 4a). In the case of the landmark genes, that phenomenon is slightly less prominent (Fig. 4b). This is particularly interesting since one of the selected cell lines is cancerous and the other is non-cancerous, suggesting that the model may avert the fallacy of using the same gene as a proxy for its gene expression across disparate biological systems. Additionally, the model does not just attribute importance to genes that are highly expressed or under-expressed, as illustrated by the distributions of the average percentage overlap of top important genes (the average is derived from averaging the overlap across all possible genes into which the input gene can be translated) for translation and top regulated genes across samples (Fig. 4C, Supplementary Fig. 16b). The overlap is lower than ~20% even for considering up to 1000 top genes, but never zero, suggesting that some, even though trivial, relationships do exist between what our model considers important and what is highly regulated, as it is also suggested by the Spearman’s correlation between the absolute importance scores and the absolute gene expression when using our model and shuffled genes (Supplementary Fig. 16a).

The simultaneously trained classifier can also be used to identify subsets of latent variables in the global latent space that are important for classifying samples by cell type. Although the cell line effect is partially filtered and embeddings coming from the same condition are globally close to each other (Supplementary Fig. 11), there are still 11 latent variables that allow the classification of cell lines using a k-means-based approach (see Methods). These latent variables can separate the samples based on cell line (Fig. 4d), even though globally the cell line-specific effect in the latent space is still partially filtered out. These 11 latent variables capture cell line-specific information; however, the latent space contains more information about other covariates too, such as drugs (Supplementary Fig. 19), conditions (Supplementary Fig. 18), time (Supplementary Fig. 20), and other information (Supplementary Fig. 21), while reducing a lot the latent dimension decreases performance (Supplementary Fig. 22), explaining why a high dimensional space was used. Genes considered important by the encoders to control these latent variables should be either cell line-specific genes or a subset of genes that can easily distinguish between cell lines. The importance scores of the genes for each cell line-specific encoder do not correlate at all and are different between the two cell lines (Supplementary Fig. 17). It is possible to even train a very simple generalized linear model to classify cell lines based on gene expression, only using a subset of these important genes, achieving high performance with only a few genes from each cell line (Fig. 4e).

More interestingly, using the model to identify important genes for translation can provide insights into the biological mechanisms of translating cell lines. On this front, we estimated transcription factors (TFs) and KEGG pathways enrichment, using Gene Set Enrichment Analysis (GSEA), based on the gradient scores signifying the importance of a gene in one cell line to translate to genes in another cell line. It is expected that to translate PC3 to HA1E (and vice versa), since one is cancerous and the other one is not, we would observe predominantly TFs whose activity is known to be regulated in cancer, and similarly, KEGG pathways linked to cancerous or inflammatory signals. This would mean that to push one cell line closer to each other, TFs associated with cancer (either activated or inhibited) should be regulated or targeted, a sensible observation from a drug development perspective. Indeed, by looking at the top 16 TFs, when translating PC3 to HA1E, we can observe TFs such as E2F2, MYC, FOXM1, RELA, JUN, FOSM, even TP53 which is often a therapeutic target of anti-cancer therapeutics, and others (Fig. 4f). Moreover, we identify cancer-associated or inflammatory KEGG pathways such as DNA replication, Mismatch repair, Cell Cycle, TNF signaling, p53 signaling pathway, and others, which are again either upregulated or downregulated in cancer (Supplementary Fig. 23). Similar results we observed when translating from HA1E to PC3, with a big overlap (68%) in the important TFs identified (Fig. 4g). One of the 4 TFs not identified when translating from PC3 to HA1E, but is found as important and significantly enriched in the other direction, is VDR, which plays a role in renal transplantation outcome^42^, as vitamin D receptors (VDRs) are expressed in kidney^43,44^, meaning that this tells us that to push HA1E, a kidney cell line, closer to PC3, we need to regulate the activity of a kidney-critical TF. All in all, these suggest that the model can be used to suggest targets to be perturbed to make the transcriptional profile of one cellular model more similar to another, and perhaps result in a more similar phenotype. The type and direction of such perturbation, together with consideration of potential off-target effects, requires further analysis and is out of the scope of this work.

### Performance in inter-species translation for lung fibrosis

**Fig. 5:**
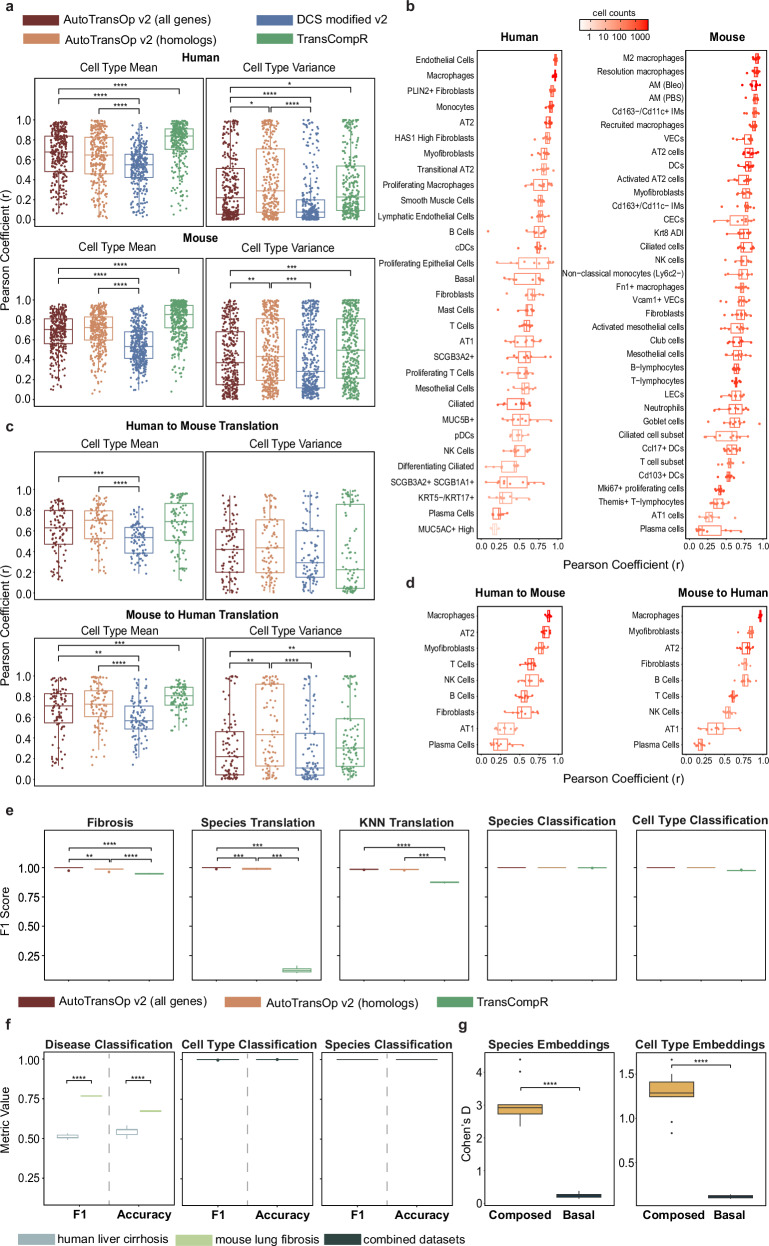
Evaluation of the framework in inter-species translation in fibrosis. **a** Performance (Pearson’s *r*) in predicting the per gene mean and variance of single-cell RNA-sequencing data for the tasks of reconstruction, across all cell types, and species translation, across common cell types, in the human-mouse lung fibrosis datasets. Only statistically significant comparisons are shown (TransCompR was not compared at all with DCS). **b** Performance of AutoTransOP v2 per specific cell type. **c** Performance comparisons for the tasks of translation in common cell types between the two species. **d** Per common cell type translation performance of AutoTransOP v2. **e** Classification performance comparison in different tasks. **f** Classification performance of the framework using all genes in external disease datasets. **g** Embeddings separation based on species and cell types in the global latent space versus the composed latent space. The effect size d is calculated as Cohen’s *d*. For all comparisons in this figure, a two-sided Wilcoxon test was used with *n* = 10 per group. In all boxplots, the centerline denotes the median, the bounds of the box denote the 1st and 3rd quantiles, and the whiskers denote points not being further from the median than 1.5 × interquartile range (IQR).

Animal models do not recapitulate human biology perfectly, so computational modeling can be used to improve the translation between human and animal models. We evaluate the ability of the framework to perform inter-species translation. We utilize the raw gene counts coming from single-cell RNA-sequencing of a mouse^36^ and human^37^ lung fibrosis dataset. The decoders predict the mean and the dispersion parameter for every gene, derived from a negative binomial distribution^45,46^. The variance is later calculated from the dispersion parameter. Furthermore, both a trainable species vector and another trainable cell type vector are added to the global space, in an attempt to minimize both species and cell type effects. We evaluate the performance in the reconstruction of gene expression profiles (per cell type) and the ability to translate between mouse and human, using common cell types, under tenfold cross-validation, in terms of Pearson’s r of the predicted per gene means and variances, where we would expect to observe a similar distribution in a successful translation, and thus mean and variance. Our framework outperforms DCS in terms of Pearson’s r of the means and variances, both in reconstruction and translation, while there is no difference using the model with ortholog genes or all genes (Fig. 5a, c). Meanwhile, TransCompR outperforms the model in reconstruction while it is not statistically better in translation, but in general, the model performs comparably (Fig. 5a, c). Possibly, TransCompR performs so much better in reconstruction because of the fewer parameters and the PCA-based space it builds. It is worth noting that based only on the human lung fibrosis dataset, three of the top ten genes contributing to the top principal components do not have homologs in mice (Supplementary Fig. 26), meaning that irrespective of performance, a method considering only homologs would exclude important genes for lung fibrosis. Finally, observing the performance per specific cell type (Fig. 5b, d), the model achieves remarkable performance, both for reconstruction and translation, for some cell types, such as Macrophages and AT2 cells, while it seems that there is a correlation between performance and the number of cells within a cell type in the whole dataset (Supplementary Fig. 27). This means that the model is performing poorly for rare cell types, but for cell types such as Macrophages, AT2 cells, and Myofibroblasts, which are associated with fibrosis or are lung-specific (AT2) and dominate the cellular population in the samples, it achieves even translating between mouse and human with a Pearson correlation greater than ~0.75.

We also evaluate the ability of each approach to classify fibrosis, species, and cell type and to classify correctly a signature as a different species when that is translated in the composed latent space, by adding a different species effect. In our framework, utilization of all genes outperforms the homolog genes approaches in predicting fibrosis and species-translation, though the performance of all approaches is high (Fig. 5e). Similar to what was observed for the L1000 dataset, species and cell type are perfectly predicted in our framework. Additionally, both the species and cell-type effects are relatively filtered (Fig. 5g, h, Supplementary Figs. 24, 25) in the global latent space, which is the space where the species and cell-type effect are filtered out from latent embedding representations, compared to the composed latent space, which is the space containing embeddings after adding again the species and cell type effect retrieved from the model using trainable vectors. This means the model succeeds in partially removing the cell type and species effect in the global latent space and then retrieving it again in the composed latent space.

### Generalization in other disease datasets

Models that are trained on a specific data set can often perform worse on external test sets, and it is, therefore, useful to investigate to which extent the model can predict disease, species, and cell types in other datasets, as well as different tissue and disease datasets. For this, we use an independent dataset on mouse lung fibrosis^47^ and a dataset on human liver cirrhosis^48^. In the mouse dataset, even though different genes were measured than those in our model, the performance is still decent in disease classification (Fig. 5d). For the human dataset, which is an extreme case of fibrosis in a different organ, the model has markedly lower performance although better than chance (Fig. 5d). Interestingly, in both cases, the model can still perfectly identify cell types and species (Fig. 5d), once again displaying the model’s ability to capture the general characteristics of the system.

### An inter-species model from serology data for predicting protection against HIV

**Fig. 6:**
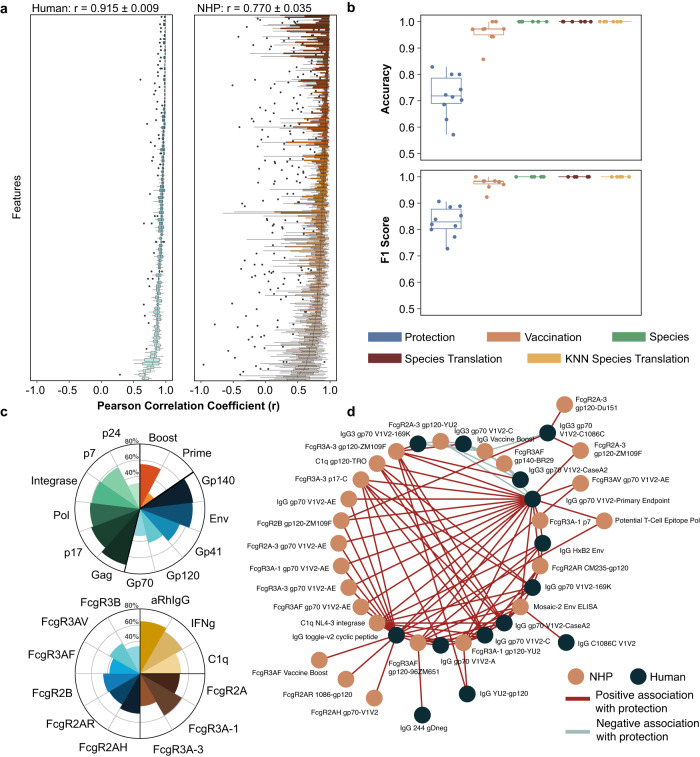
Inter-species translation of serology data. **a** Per feature Pearson correlation in ten fold cross-validation for human features and for non-human primate (NHP) features. **b** Classifiers’ performance in various tasks (*n* = 10 per task). In all boxplots, the centerline denotes the median, the bounds of the box denote the 1st and 3rd quantiles, and the whiskers denote points not being further from the median than 1.5 × interquartile range (IQR). **c** Functional grouping of NHP features predictive of protection-associated human features. In the top nightingale rose plot, NHP features are categorized by antigenic target. Blue features are antigens found on the surface of the HIV virus, green features are internal viral components, and orange features are specific to the antigens present in the primary vaccine (Prime) and booster vaccine (Boost). In the bottom nightingale rose plot, NHP features are categorized by serological feature type. Brown features are binding affinities specific to the human Fc-receptors, blue features are binding affinities specific to the NHP Fc-receptors, and yellow features are either anti-Rhesus IgG titers or functional assays (IFNg Elispots; C1q complement assays). In both plots, median percentile rank is plotted for all features belonging to each category. **d** Network visualization of the associations between specific NHP and human serological features, related to human protection. Brown nodes represent NHP features and dark blue nodes represent human features. Red and blue edges are connected to NHP features that are positively or negatively associated with protection in humans, respectively. Only the strongest NHP-human feature pairs are visualized here (see the methods for selection criteria).

As a final case study, we developed a model for cross-species translation of serology data, where there is no 1-1 mapping of features, to predict vaccine-induced protection from HIV in humans. Previous failed HIV vaccine trials have suggested that neutralizing antibody titers, the primary outcome for most vaccine trials, do not consistently correlate with vaccine efficacy^49^. Moreover, recent research suggests that deeper characterization of the antibody response, including antibody subtype prevalence and Fc-receptor binding affinity, may be necessary to predict the quality of the vaccine response^50^. Notably, a crucial difficulty in comparing pre-clinical animal models and human clinical trial data in this context is that antibodies and Fc-receptors with similar names across species can be categorically distinct, disparate in both structure and functions between species, such that for numerous proteomic features orthologous features do not exist. Our ANN approach has the potential to advance our understanding of which preclinical features might best predict the efficacy of an HIV vaccine. Here, we utilize serology data from non-human primate (NHP) and human datasets^38–40^ following vaccination against SHIV and HIV, respectively. In line with other models constructed using this framework, the model was trained so that protected individuals are close to each other in the global latent space, regardless of species. We utilize AutoTransOP v2, with a small modification, where two separate classifiers try to predict vaccination status and protection in the global space, and a third classifier predicts species in the composed latent space. For the human serology features, the model has high performance when reconstructing each feature (Fig. 6a, *r* = 0.92 ± 0.01). Ιn NHPs, while some features are not predicted well and there is a big variation in performance between folds, the overall performance is still good (Fig. 6a, *r* = 0.77 ± 0.04). Finally, the performance across all classification tasks is exceptionally high (Fig. 6b), including 100% accuracy in species classification and translation, which is evaluated by how well the species classifier predicts species label when translating a signature to another species in the latent space.

Using the model, we aimed to identify features from both species that are predictive of human protection. For this, we performed the integrated gradient approach in parallel to likelihood ratio tests (LRT) on each latent variable (see methods). Latent variables are denoted as important in predicting human protection only if there is an agreement between the likelihood ratio test results and the integrated gradients (Supplementary Fig. 33). The human features identified indeed have a statistically significant difference between protected and non-protected individuals (Supplementary Fig. 34a). Finally, we identified NHP features that have a high gradient score when translating to human signatures, meaning these NHP features are predictive of human features linked with viral protection (Fig. 6c, d). These features are not necessarily associated with NHP protection (Supplementary Fig. 34b) but they could be predictive of human protection. Notably, while the top human features identified are generally related to V1V2-specific IgG titers, the top NHP features include a wide range of feature types, including Fc receptor binding, interferon gamma (IFNg) elispots, and IgG titers. Classification of NHP features by antigenic target revealed some surface antigens (Gp140, Env) and intracellular antigens (Gag, p17) to be similarly important in predicting the top human features (Fig. 6c). When classified by feature type (IgG titer, Fc gamma receptor (FcgR) subtype, etc), IgG titers, interferon-gamma (IFNg) elispots, and human FcgR3A-1 show higher median importance for human prediction relative to the NHP FcgRs. Certain NHP features are consistently highly associated with specific top human serological features, and in some cases (e.g., IgG gp70-V1V2) we observed multiple NHP features mapping to one human feature while in other cases only one NHP feature is identified as strongly associated (e.g., IgG 244 gDneg) (Fig. 6d, Supplementary Fig. 35). Notably, there is only one pair of homologous features in this network of important NHP and human features (IgG gp70 V1V2), highlighting the utility of our model’s ability to identify relationships between unmatched features from different species. Our analysis is both consistent with previous studies and identifies novel feature associations across species. An especially important result is that we can ascertain particular NHP immune system features as indicative of non-orthologous human immune system features providing important contributions to protection, which can aid in identifying serological biomarkers in NHPs that are highly predictive of human HIV vaccine efficacy.

## Discussion

Here, we develop AutoTransOP, an ANN framework that facilitates the translation of omics profiles between different biological systems. The framework combines ideas from the CPA approach^32^ and other species and cellular translation methods^13,15,18,31^, together with ideas from language translation models^33^. The explicit goal is to align omics signatures between systems, rather than identifying what information inherent in the signature of one system is most germane for understanding phenotype characteristics in the other, which has been the objective in many previous studies^16–19^. The framework performs as well as (or even better than) other state-of-the-art translation techniques, when using homolog features between systems, and performs similarly without a 1-1 mapping between features. Notably, the framework constructs a relatively global latent space with stimuli-specific regions, for which classifiers can be jointly trained to make predictions for various tasks, such as the diagnosis of diseases.

Most current approaches to translating between systems require homolog features and utilize linear transformations to facilitate translation^13–18^, and are thus restricted to represent linear inter-species relationships. Also, the non-linear ANN-based approach DeepCellState^31^ requires homology of the molecular features used to describe the biological systems. In contrast, our framework can represent non-linear relationships between different biological systems, without requiring any kind of homology, and achieves high performance using only a small percentage of paired conditions. This enabled us to train a translation model on serology datasets for which a 1-1 mapping of the features between the two biological models did not exist. Through interpretation of this model, relationships between very different molecular profiles that correlate with specific phenotypes can be identified, e.g., protection against infection.

Interpretability of deep learning models in biology remains a challenge. These models have been criticized for providing a poor understanding of which biological relationships they capture^51,52^. On this front, we demonstrate in our framework how integrated gradient approaches^35^ can be used to estimate the importance of features used by different parts of the framework for various tasks, enabling some biological interpretation of the model. Based on this, we could propose serological features predictive of human protection against HIV, including non-human primate-specific features that can be observed in the preclinical stages of vaccine development. Finally, elements of the framework can be used to interpret and successfully retrieve the effects of species or cell types, filtered from the global latent space. This can explain the ability of the framework to predict cell types and species with high performance also in independent disease datasets, derived from different organs/tissues. However, there are still limitations in the generalization of in the models to external datasets. In particular, the performance on such datasets drops significantly as samples from different pathologies and tissues are considered. Even within the same disease, the inclusion of different features can lead to reduced performance in predicting disease diagnosis.

Despite our framework being trained successfully on datasets with relatively small sample sizes, the model still contains many parameters, especially when using a larger number of features, which inevitably leads to overfitting. Some of these shortcomings could likely be alleviated by applying our framework to larger datasets, such as ARCHS4^10^, which contains hundreds of thousands of publicly available RNA-sequencing data from humans and mice. Training with more data and more diverse unique conditions may enable higher generalization and higher granularity in modeling different biological covariates. Another approach would be to adapt from the Cross-Domain Structural Preserving Projection (CDSPP)^53^ method, but in a non-linear manner, machine learning approaches that require fewer samples, such as modifications of the recently proposed Species-Agnostic Transfer Learning (SATL)^54^, where the model learns a linear projection matrix for a domain-invariant feature subspace, in order to build the global latent space. Additionally, with the advent of Natural Language Processing (NLP) models^55^ and attention-based models^56^, our encoder modules could potentially be modified with NLP-like representations. Recently, Geneformer^57^, an attention-based model, was pre-trained on a corpus of 30 million single-cell transcriptomic profiles and was proven to be context-aware of the system it encodes. Although it still requires some level of homology, it paves the way to utilize NLP approaches for transfer learning in biology, and ultimately translation.

The flexibility of our framework allows the modeling of many different biological systems. This could lead to the computational optimization of biological systems and assays aiming to model human pathology. Using our framework, we can both explore potential transcriptional modifications to design better disease models and identify features predictive of human biology without requiring homology between systems, ultimately reducing resources spent during experimental modeling and potentially expediting the translation of in-vitro and preclinical findings to human therapeutic advancement.

## Methods

### Preprocessing of in-vitro transcriptomics benchmark dataset

The L1000 CMap resource^12^ contains bulk gene expression data from drug perturbations across different cell lines and provides a benchmark dataset with diverse conditions and a large sample size (for a total of 720,216 samples of drug perturbations of varying quality). Additionally, several equivalent perturbations across different biological systems are available (406 *paired conditions* for the case of A375 and HT29 cell lines after filtering and pre-processing, explained below) to evaluate the performance in translating omics profiles. We selected high-quality drug perturbations from the latest version of the L1000 dataset (accessed via clue.io). The level 5 z-score transformed and pre-processed differential gene expression data of 978 landmark genes, measured with the L1000 assay, and additionally, 9196 computationally inferred genes in the CMap resource that were marked as well-inferred, were considered in the subsequent analysis. We consider perturbations as high-quality if they consist of signatures with more than three replicates, where at least half of them passed the standard quality control protocols in the assay, as provided in the dataset, and were not identified as statistical outliers (as considered by the L1000). Additionally, where multiple-signature perturbagens, i.e., technical replicates, only the signature with the highest transcriptional activity score (TAS) across these technical replicates was retained in the dataset, these signatures are labeled “exemplars” by the CLUE platform and are specifically designated for further analysis by the platform^58^. The TAS metric is provided along with the L1000 dataset and quantifies signal strength and reproducibility. Finally, the ability to distinguish between random pairs of signatures and true biological replicates, meaning the same perturbagen tested on the same cell line for the same duration and dosage, was evaluated for different parts of the dataset, split using varying TAS thresholds (Supplementary Fig. 38) and samples with a TAS ≥ 0.3 were retained. After filtering 13,699 samples remained, with 1107 conditions available in total for the HT29 cell line and 1213 for the A375 cell line. In the case of control signatures, we followed the same procedure but without filtering based on TAS.

### Preprocessing single-cell RNA sequencing interspecies datasets

For the human and mouse single-cell RNA-sequencing datasets, we first re-annotated manually each annotated cell into one of the four classes: i) immune cells, ii) mesenchymal cells, iii) epithelial cells, iv) endothelial cells, and iv) stem cells. These high-level labels were later used to remove cell effect from the global latent space and were also used in the subsequent cell-type classification. Finally, the gene expression count data were log-transformed (${x}_{input}={\log }_{10}(count+1)$) to rescale and reduce the dynamic range and skewness of the data, also avoiding this way extreme values which could potentially lead to extreme values of the weights of the models. Moreover, this process attempts to stabilize the variance of the data. We have to note, however that this transformation is not statistically better in terms of performance, even though it has slightly better performance (Supplementary Fig. 39).

### Preprocessing of the serology datasets

For all serology data, we aimed to construct a model using only antibody and receptor measurements. The human data were retrieved from Chung et al.^40^ upon request, the avidity molecular features were dropped and the data were z-scored per feature. The non-human primates’ data were retrieved from Barouch et al.^38^ upon request, the samples taken in week 28 were used, and antibody-dependent cellular function features and mass spectrometry data were dropped. The data were log-transformed ($x={\log }_{10}(MFI+1)$), the median per feature from controls was subtracted from each feature to standardize the data. Finally, the data are z-scored per feature.

### The general framework and the training procedure

In this implementation (also described with pseudo-code in Supplementary Note 1), the framework always models pairs of systems for translation, species, or cell lines. Each is modeled with separate encoders and decoders for each of the species or cell lines in the pair attempting translation, while inside a latent module, the global latent space is shaped (Fig. 1a). Both the encoders and the decoders are multi-layered neural networks, with each layer consisting of, sequentially: a fully-connected layer, a batch normalization layer^59^, an ELU activation function^60^, and a dropout layer^61^. The final output layer of the encoder and the decoder consists of only one fully connected layer without a trainable bias term.

For the construction of the global latent space (pseudo-code in Supplementary Note 1) several metrics are optimized: the distance (${{\boldsymbol{L}}}_{{\boldsymbol{distance}}}$) between embeddings of profiles coming from different systems undergoing the same perturbation is minimized and their cosine similarity (${{\boldsymbol{L}}}_{{\boldsymbol{cosine}}}$) and mutual information (${{\boldsymbol{L}}}_{{\boldsymbol{MI}}}$, see details below) is maximized; and the divergence of the distribution of the latent variables from a random uniform distribution is also maximized (${{\boldsymbol{L}}}_{{\boldsymbol{prior}}}$). Even though in the literature^62–64^, at least, when only having encoders part to create embeddings of an input structure, it is suggested to minimize divergence from a uniform distribution, we decided to do the opposite due to evidence that the embeddings tend to resist enforcing a uniform distribution without a significant loss of performance (Supplementary Fig. 36). Both cosine similarity and Euclidean distance losses were added to enforce the strongest possible filtering of species and cell type effect, while the cosine similarity also enforces normalization of the latent embeddings. Mutual Information (MI) maximization is achieved using two different ANN discriminators, as previously proposed in the MINE^62^, Deep InfoMax^63^ and InfoGraph^64^ studies, where the Jensen-Shannon Mutual Information between embeddings coming from the same perturbation is estimated and the extra prior loss is calculated and added in the final loss, according to the following equations with the implementation taken from the deepSNEM model^65^ and the GitHub repository of Deep InfoMax^63^ https://github.com/rdevon/DIM/tree/master/cortex_DIM/functions:

1 $${L}_{prior}=\frac{1}{N}{\sum }_{i=1}^{N}[\log (Disc{r}_{2}({v}_{i}))+\,\log (1-Disc{r}_{2}({z}_{{g}_{i}}))],$$

where ${v}_{i}$ is a randomly sampled embedding from a prior random uniform distribution ranging from 0 to 1 and ${z}_{{g}_{i}}$ is a global latent space embedding. N is the number of samples in a batch during training.

2 $${L}_{MI}=-({E}_{p}-{E}_{q}),$$

3 $${E}_{p}=\frac{1}{\sum (mask)}[ln(2)-softplus(-Dis{cr}_{1}({z}_{g})\odot mask)]\odot mask,$$

4 $$Eq=\frac{1}{\sum (1-mask)}[softplus(-Dis{cr}_{1}({z}_{g})\odot (1-mask))+Dis{cr}_{1}({z}_{g})\odot (1-mask)-\,\mathrm{ln}(2)]\odot (1-mask),$$

where *E*_p_ and *E*_q_ are respectively the mutual information estimates, adapted from the GitHub of Deep InfoMax^63^ (https://github.com/rdevon/DIM/tree/master/cortex_DIM/functions), between pairs derived from the same conditions and pairs coming from different conditions, averaged for every possible pair in a batch during training. *z*_*g*_ are global latent space embeddings, whose pairwise mutual information is estimated using the Discr_1_ discriminator and mask is the mask of positives (similar conditions) created in each batch of training procedure. ${{\boldsymbol{L}}}_{{\boldsymbol{MI}}}$ serves as an estimate for maximizing the difference, between similar and different conditions, of a lower bound of mutual information. The actual calculation of MI is not the goal here. Indeed, this maximization was achieved (Supplementary Fig. 3).

Both Discr_1_ and Discr_2_ utilize non-KL-divergence approaches as suggested in the literature^62–64^. Discr1 is the discriminator that generates a probability score between every sample in the batch and all others, during training and is used to estimate the mutual information between two embeddings from the global latent space^63^. It takes as input two global latent space embeddings and passes them through the same three fully-connected layers, each of them followed by a ReLU activation function^60^ and one fully-connected skip connection. Then, the product of the result of this non-linear transformation of the two embeddings is the output of Discr1 and is passed through the softplus activation function ($g(x)=\,\log (1+{e}^{x})$), to ultimately be used approximate the Jensen-Shannon lower bound of their Mutual Information, as proposed originally in MINE^62^ and Deep InfoMax^63^. Discr_2_ is the second discriminator which takes as input an embedding vector and calculates the probability a point in this embedding space is sampled from a specific distribution. This way ${L}_{prior}$ forces each feature of the learned embeddings to be sampled from a distribution which is not the random uniform distribution ranging from 0 to 1, as a small analysis showed that it is difficult to enforce a uniform distribution without taking a hit in performance (Supplementary Fig. 36) and the latent embeddings tend to assume a normal distribution (Supplementary Fig. 36b), not necessarily with a mean of 0 and a standard deviation of 1. However, it is important to note that using a prior loss is not necessary as it makes not real difference in the distribution and performance of the model (Supplementary Fig. 37). It has three similar fully-connected layers and the final scalar output is passed through a sigmoid activation function^60^. These regularization loss terms (${{\boldsymbol{L}}}_{{\boldsymbol{distance}}}$, ${{\boldsymbol{L}}}_{{\boldsymbol{cosine}}}$, ${{\boldsymbol{L}}}_{{\boldsymbol{MI}}}$) are calculated and averaged across every pair of global embeddings (${z}_{{g}_{i}},\,{z}_{{g}_{j}}$) that are coming from the same condition. The ${{\boldsymbol{L}}}_{{\boldsymbol{prior}}}$ is calculated for every sample in the dataset, meaning every global latent embedding and averaged across samples. For the case of the L1000 dataset, we consider similar perturbations those that are coming from experiments of the same drug, tested on the same cell line, with the same dose and time duration. For the lung fibrosis dataset, similar profiles are considered those coming from samples that have the same diagnosis (fibrosis or not). For the serology datasets, we train the framework so that embeddings coming from protected individuals against HIV are close to each other regardless of species (and even vaccination status)

The basic task of this autoencoder framework is reconstruction, which is achieved by minimizing some kind of reconstruction loss (${{\boldsymbol{L}}}_{{\boldsymbol{recon}}}$). In the case of z-scored profiles from bulk data, this is done by minimizing the mean sum of squared errors between the input of the encoders and the output of the decoders. The sum of squares error is averaged across samples. For only the case of single-cell RNA-sequencing data, based on the implementation proposed in the CPA manuscript^32^ (found here https://github.com/facebookresearch/CPA), the negative binomial negative log-likelihood is used to optimize the reconstruction, by assuming that the data are derived from a negative-binomial distribution characterized by the mean and the dispersion parameter that are both predicted, while the goal is to reconstruct the original count matrix^45,46^. The negative binomial negative log-likelihood loss is calculated for every sample and the average across all samples in the batch is minimized. Finally, while the raw gene counts are used for reconstruction from the decoder in the loss function, the encoders take as input the log-transformed counts, and initially perform an element-wise multiplication between genes and a set of trainable weights, before feeding the data into the feedforward neural network layers of the encoders.

Classifiers are used for different classification tasks. These consist of multiple fully connected layers and a final SoftMax activation function before the output. The average entropy loss across samples for every classification task in the latent space is minimized:

5 $$entrop{y}_{i}=\frac{1}{N}{\sum }_{j=1}^{N}CrossEntropy(Classifie{r}_{i}({z}_{j}),\,labe{l}_{j}),$$

where $entrop{y}_{i}$ is the average cross entropy between every *j*th prediction of a classifier taking a latent vector as input and the true label for that sample.

L2-regularization of the weights and bias of the encoders (${\boldsymbol{L}}{2}_{{\boldsymbol{encoder}},{\boldsymbol{i}}}$), decoders (${\boldsymbol{L}}{2}_{{\boldsymbol{decoder}},{\boldsymbol{i}}}$), and classifiers (${\boldsymbol{L}}{2}_{{\boldsymbol{classifier}},{\boldsymbol{i}}}$) is also enforced by minimizing the sum of squares for the aforementioned trainable parameters.

Taken together, for the basic variation (AutoTransOP v1, described in pseudo-code in Supplementary note 1), the following loss function is optimized:

6 $${\boldsymbol{Los}}{{\boldsymbol{s}}}_{{\boldsymbol{basic}}}={\lambda }_{recon}\ast {L}_{recon}+{\lambda }_{distance}\ast {L}_{distance}+{\lambda }_{MI}\ast {L}_{MI}+{\lambda }_{prior}\ast {L}_{prior}+{\sum }_{i=1}^{2}({\lambda }_{enc,i}\ast L{2}_{encoder,i})+{\sum }_{i=1}^{2}({\lambda }_{dec,i}\ast L{2}_{decoder,i})+{\sum }_{i=1}^{M}({\lambda }_{L2class,i}\ast L{2}_{classifier,i})+{\sum }_{i=1}^{M}({\lambda }_{class,i}\ast entrop{y}_{i})-{\lambda }_{cosine}\ast {L}_{cosine},$$

where *M* is the number of classifiers and thus individual classification tasks, and the rest of the terms, together with how they are calculated, have already been described in the previous paragraphs of this section. For values for each of the *λ* used in the loss function, see Supplementary Table 5.

### Variation of the global latent space with a simultaneously and competitively trained classifier

For AutoTransOP v3, the variation of the global latent space with a simultaneously and competitively trained classifier, the aim is to embed some species or cell line information in some of the latent variables. A simple classifier for correctly predicting the cell line label is trained simultaneously on the global latent space with the rest of the framework and an entropy loss is added to the original description of the framework. The construction of a global latent space and the training of the classifier are competing tasks, where the framework is trained to achieve a stable trade-off.

### Variation of the framework, including elements of the CPA approach

For AutoTransOP v2, the variation of the framework, which incorporates the elements of the CPA approach, the global latent space is expanded by augmenting the loss function with some additional terms.

An adverse classifier of species and cell types is added. As described in the original CPA manuscript^32^, during training we iterate between training the classifier (updating only its parameters) on the global latent space, and training the rest of the framework with the addition of a penalty (${\boldsymbol{entrop}}{{\boldsymbol{y}}}_{{\boldsymbol{adverse}}}$) if the classifier correctly classifies species and cell types. To improve the robustness of the discriminator it is initially pre-trained only with encoders and discriminators, without other classifiers and the decoders, so that it can already distinguish cell types and species in the global space.

Furthermore, species and cell type effects are added to the latent space via trainable vectors. In the newly composed latent space, from which the decoders are sampling embeddings, classifiers are jointly trained to correctly classify cell types, and species (or even disease diagnosis). Additionally, the trainable vectors are regularized by the L2 norm (${\boldsymbol{L}}{2}_{{\boldsymbol{traine}}{{\boldsymbol{d}}}_{{\boldsymbol{effect}}}}$). All the above can be summarized in this new loss function:

7 $$Loss=Los{s}_{basic}-{\lambda }_{adverse}\ast entrop{y}_{adverse}+{\lambda }_{traine{d}_{effect}}\ast L{2}_{traine{d}_{effect}}$$

### Framework for the serology datasets

In the serology dataset, we use AutoTransOP v2, where now is aimed to later identify features predictive of protection or vaccination status regardless of species. For this purpose, we train two classifiers predicting vaccination and protection status in the global latent space. We care more about protection and thus, as described previously, we aim to create similar embeddings and minimize their distance in the global latent space just by looking at protection status.

### Framework’s basic hyperparameters

Here, we present the basic parameters used to train the model (Table 1). No thorough hyperparameter tuning was performed, and values were selected based on empirical values and tuned so that there is convergence in the training loss and the training reconstruction performance (Pearson’s *r*). Additionally, these values were also tuned so that the performance in training (not validation/test) is sufficiently high, meaning that the model is at least able to fit the given data. This empirical tuning was done only based on the 1st training set in tenfold cross-validation. We performed tenfold cross-validation (where the validation sets are used as test sets to only evaluate the models) where 10% of the data were hidden for validation each time and 90% for testing. The 10% changes each time, so all data have at some point been put in the set for evaluating the models. All the parts of the framework are trained simultaneously.

**Table 1:** Framework’s basic hyperparameters

Hyperparameter	L1000: 978 genes	L1000: 10,086 genes	Lung fibrosis	Serology
Latent dimension	292	1024	512	32
Hidden encoder layers dimensions	[640,384]	[4096,2048,1024,512]	[4096,2048,1024,512]	[64]
Hidden decoder layers dimensions	[384,640]	[512,1024,2048, 4096]	[512,768,2048, 4096]	[64]
Cell type classifier hidden layer dimensions	[256,128,64]	[512,256,128]	[256,128,64,32]	-
Species classifier hidden layer dimensions	-	-	[256,128,64,32]	[32,16,8]
Fibrosis classifier hidden layer dimensions	-	-	[256,128,64,32]	-
Serology phenotype classifiers classifier hidden layer dimensions	-	-	-	[32,16,8]
Adverse classifiers hidden layers dimensions	[256,128,64]	[512,256,128]	[512,256,128,64]	[32,16,8]
Total batch size	512	512	1024	50
Number of epochs	1000	1000	200	2000
Learning rate	0.001	0.001	0.001	0.001

The latent space dimension was chosen to be as small as possible until the model’s performance dropped in both training and validation of only the 1st fold. Based on this latent dimension and the original input dimension of the data the sizes of hidden layers of the encoders were chosen to be in-between, gradually reducing the input dimension to that of the latent space. The actual size and number were constrained by practical memory limits. With the exception of the lung fibrosis models, the decoders had the same number and sizes of hidden layers as those of the encoders, but now they increase the size of the embeddings from the latent dimension to the original input dimension.

### Evaluation procedure and metrics

The model performance was evaluated using tenfold cross-validation. One fold (10%) of the data was hidden during training and used to evaluate performance in unseen data, and 90% of the data from each system (species or cell line in the case of L1000) were used for training. For the L1000 dataset, for evaluating the translation of the whole omics profile, we made sure that for the case of paired conditions, the perturbation in both cell lines was hidden during training.

The classification tasks were evaluated by total accuracy and F1-score (or micro F1 for multiple categories):

8 $${\rm{Accuracy}}=\frac{{\sum }_{{\rm{i}}=1}^{{\rm{K}}}{{\rm{TP}}}_{{\rm{i}}}+{\sum }_{{\rm{i}}=1}^{{\rm{K}}}{{\rm{TN}}}_{{\rm{i}}}}{{\sum }_{{\rm{i}}=1}^{{\rm{K}}}{{\rm{TP}}}_{{\rm{i}}}+{\sum }_{{\rm{i}}=1}^{{\rm{K}}}{{\rm{TN}}}_{{\rm{i}}}+{\sum }_{{\rm{i}}=1}^{{\rm{K}}}{{\rm{FP}}}_{{\rm{i}}}+{\sum }_{{\rm{i}}=1}^{{\rm{K}}}{{\rm{FN}}}_{{\rm{i}}}},$$

9 $${\rm{F}}{1}_{{\rm{micro}}}=\frac{{\sum }_{{\rm{i}}=1}^{{\rm{K}}}{{\rm{TP}}}_{{\rm{i}}}}{{\sum }_{{\rm{i}}=1}^{{\rm{K}}}{{\rm{TP}}}_{{\rm{i}}}+\frac{1}{2}\ast ({\sum }_{{\rm{i}}=1}^{{\rm{K}}}{{\rm{FP}}}_{{\rm{i}}}+{\sum }_{{\rm{i}}=1}^{{\rm{K}}}{{\rm{FN}}}_{{\rm{i}}})},$$

where *K* is the total number of classes in multi-class classification, TP and FP symbolize true and false positives, and TN and FN symbolize true and false negatives. For the case of multiple classes, we define as positives the samples belonging to that specific class while everything else is a negative sample. Using this definition of positives and negatives we further calculate the TP, FP, TN, and FN per class. In the case of cell-type classification in lung fibrosis *K* = 5.

For the cell line classification in L1000, species classification both in lung fibrosis and the serology datasets, and vaccination and protection status in the serology dataset, we use the F1 score and accuracy for binary classification

10 $${\rm{Accuracy}}=\frac{{\rm{TP}}+{\rm{T}}N}{TP+TN+FP+FN},$$

11 $$F1=\frac{TP}{TP+\frac{1}{2}(FP+FN)}.$$

To evaluate the validity of the predictions ($\hat{{\rm{y}}}$) of whole signatures in translation and reconstruction, compared to the ground truth (*y*), we utilized:

i. the global Pearson’s correlation
  12 $${\rm{r}}(\hat{y},{\rm{y}})=\frac{\sum ({{\rm{y}}}_{{\rm{i}}}-\overline{{\rm{y}}})({\hat{{\rm{y}}}}_{{\rm{i}}}-\overline{\hat{{\rm{y}}}})}{\sqrt{\sum {({{\rm{y}}}_{{\rm{i}}}-\bar{{\rm{y}}})}^{2}\sum {({\hat{{\rm{y}}}}_{{\rm{i}}}-\overline{\hat{{\rm{y}}}})}^{2}}},$$
  where $\hat{{\rm{y}}}$ and y are flattened and the *i*th element is the *i*th point in these flattened vectors.
ii. the average per sample Spearman’s correlation
  13 $${{\rm{r}}}_{{\rm{s}}}=\frac{{\sum }_{{\rm{i}}=1}^{{\rm{N}}}{\rm{r}}{({\rm{Rank}}(\hat{{\rm{y}}}),{\rm{Rank}}({\rm{y}}))}_{{\rm{i}}}}{{\rm{N}}},$$
  where *N* is the number of samples and Rank() means ranking the gene based on their differential gene expression and using these ranks to calculate Spearman’s correlation.
iii. the average per sample

14 $${\rm{sign}}\,{\rm{accuracy}}=\frac{{\rm{TP}}+{\rm{TN}}+{\rm{TrueZeros}}}{{\rm{total}}\,{\rm{predictions}}},$$

where TP signifies the genes that have a positive sign regulation both in the actual data and predictions, TN signifies the genes in the sample that have a negative sign regulation both in the actual data and predictions, and TrueZeros are the genes that have an absolute expression ≤10^−6^ both in the actual data and predictions (a small tolerance rather than strictly zero was chosen for numerical reasons).

For the single-cell RNA-sequencing data where we predict the per gene mean and variance, we calculate the coefficient of determination (*R*^2^) per gene mean and variance, similar to the CPA manuscript^32^. In general, *R*^2^ is calculated as:

15 $${R}^{2}=1-\frac{RSS}{TSS},\,where\,RSS=\sum {({\hat{{\rm{y}}}}_{{\rm{i}}}-{y}_{i})}^{2}\,and\,TSS=\sum {({{\rm{y}}}_{{\rm{i}}}-\overline{{\rm{y}}})}^{2}$$

### Separation of latent space embeddings

To evaluate the similarity of embeddings for different signatures, and whether there is separation based on cell, species, or conditions in the latent space, we utilize cosine distance, ranging from 0 (the same) to 2 (completely) different:

16 $$cosine\,distance=1-cosine\,similarity=1-\frac{{\sum }_{{\rm{i}}=1}^{{\rm{d}}}{{\rm{z}}}_{1,{\rm{i}}}{z}_{2,i}}{\sqrt{{\sum }_{{\rm{i}}=1}^{{\rm{d}}}{{\rm{z}}}_{1,{\rm{i}}}^{2}}\sqrt{{\sum }_{{\rm{i}}=1}^{{\rm{d}}}{{\rm{z}}}_{2,{\rm{i}}}^{2}}},$$

where z_1_ and z_2_ are two latent space vectors to be compared and *d* is the total number of elements in the vector, i.e., the latent dimension.

To estimate if there is a cell, species, or condition effect, and compare it between the composed and global latent space we utilize Cohen’s *d* to estimate the effect size between the distributions of cosine distances, derived from random pairs of embeddings and pairs coming from the same cell, species, or condition. The effect size is thus calculated using the mean and standard deviations of two cosine distance (cos) distributions as:

17 $$d=\frac{{\overline{cos}}_{1}-{\overline{cos}}_{2}}{\sqrt{\frac{(({n}_{1}-1){s}_{1}^{2}+({n}_{2}-1){s}_{2}^{2})}{{n}_{1}+{n}_{2}-2}}},$$

where *n*_1_, *n*_2_ is the number of samples of each of the two distance distributions, ${\overline{cos}}_{1},\,{\overline{cos}}_{2}$ are the means of the cosine distance distributions and *s*_1_, *s*_2_ are the standard deviations of the cosine distance distributions. A Cohen’s *d* around 0.8 is a large effect size (around two is considered a huge effect size) while around 0.5 is a medium effect size, and around 0.2 and below is considered small or very small^66,67^.

### Feature importance using integrated gradients

To estimate the importance of features, we utilize integrated gradients^35^ from the Captum library^68^.

18 $$InterGra{d}_{i}(x)=({x}_{i}-{x}_{i}^{{\prime} }){\int }_{a=0}^{1}\frac{dF(x^{\prime} +a(x-x^{\prime} ))}{d{x}_{i}}da,x^{\prime} =baseline=0$$

The importance scores are calculated based on the gradient with respect to the input of the model, and thus, the higher the absolute integrated gradient the higher the importance of that input feature to control the output. A negative score means the variable has a negative effect pushing the prediction to the other class, while a positive score has a positive effect.

For example, if we want to identify important latent variables to classify a sample as one coming from a particular cell line, we calculate the integrated gradient of every latent variable to make the classification and take the average across all samples. Similarly, if for example, we are aiming to calculate the importance of genes to control latent variables in the global latent space, we can calculate the integrated gradient score of every gene for every variable in every sample, and then take the average across samples.

### K-means-based separation of important latent variables

Latent variables can be separated into important and unimportant ones using k-means, inspired by an approach that was used to identify important connections between latent components and genes in microbial organisms by using the weights derived from independent component analysis^69,70^. We assume that only two main clusters of latent variables exist, one containing important variables and one containing unimportant ones. On this front, the latent variables are clustered based on their absolute gradient scores into three clusters, where 3rd cluster is assumed to be a very small cluster of outliers. The midpoint between the variable with the highest score in the unimportant cluster and the variable with the lowest score in the important cluster is used as a threshold to distinguish between significantly important and unimportant latent variables. As a sanity check the important variables are also compared with the top-ranked variables based on their score.

### Likelihood ratio tests for the identification of important latent variables

To identify which latent embeddings correlate with viral protection after accounting for vaccination status and species, a LRT was performed on each individual latent variable. Here, the likelihood (L) of the alternative model $({H}_{A}):\,latent\,variabl{e}_{i}\,embeddings \sim protection+vaccination+species$ was compared to the likelihood of the nested model, or null hypothesis,

19 $$({H}_{0}):\,latent\,variabl{e}_{i}\,embeddings \sim vaccination+species\,{\rm{in}}\,LRT=-2\,\mathrm{ln}\left(\frac{L({H}_{0})}{L({H}_{A})}\right).$$

We rejected *H*_0_ for $latent\,variabl{e}_{i}$ when the FDR-adjusted *p* value of the chi-square test was less than 0.05, concluding that the model including protection has a statistically significant better fit than the model without protection. In the volcano plots, the $-\,\log (pvalue)$ is plotted against the *t* value for the protection term in the alternative model. This method assumes that the relationship between the latent variable embeddings and protection is linear. R package *lmtest*^71^ (version 0.9.40) was used to perform these statistical tests. Finally, the intersection of these latent variables with significant latent variables (average percentage importance score across folds ≥10%), based on their gradient score from the trained protection classifier, is used for the final identification of robust latent variables associated with viral protection. We keep latent variables that the sign of correlation with protection agrees in both approaches.

### Identification of protection-associated serological features

The importance of the serological features is calculated as previously described with the integrated gradient score of every feature for every latent variable that was identified to be statistically significant for predicting viral protection of humans, averaged across samples coming from the respective species. Serological features with high scores (and at least ≥20%) can control latent variables in the global latent space associated with human viral protection, and thus they are predictive of human protection. For human features, we also validate that the univariate differences between protected and unprotected individuals are indeed significant, by using a non-parametric Wilcoxon test, with Bonferroni correction for multiple hypothesis testing.

Finally, we calculate the integrated gradient score for translating each non-human primate serological profile to a human profile. The non-human features with high scores in association with the top human features can be considered serological NHP predictive of human viral protection. The Nightingale Rose plots were constructed by categorizing each NHP feature by antigenic target or feature type and subsequently calculating the median percentile rank of each NHP feature per category. Percentile rank was calculated from the mean importance values of each NHP feature for translating to each protection-associated human feature and averaged across these human features. To take a more granular look at the relationship between each NHP feature and human feature, we constructed a network of the top NHP-human feature pairs that pass the following criteria: (1) the NHP feature reconstruction Pearson correlation coefficient was greater than or equal to 0.75, (2) the standard deviation of the NHP feature importance across folds was in the bottom quartile of NHP-human feature pairs, (3) the NHP feature had an importance score with magnitude greater than 10 in relation to at least one of the top human features, and (4) the NHP feature importance score was in the top quartile of scores that passed criteria (1–3). Using these criteria, 88 NHP-human feature pairs were identified as the most consistently important features for predicting human protection. The network representation of these 88 NHP-human feature pairs was created using Cytoscape version 3.10.1.

### DeepCellState method and variations

The original model, as developed by Umarov et al.^31^, is an autoencoder neural network framework, which consists of one common encoder and two separate decoders, one for each cell line or species in our case. The model aims to encode every gene expression profile into a common cell line space. The input gene expression is first passed through a dropout layer with a dropout rate of 0.5 and then the encoder consists of fully-connected feedforward neural network layers. The decoders are similar and consist of fully-connected feedforward neural network layers that reconstruct the input gene expression using the latent space representation. The output layer has a direct connection to the dropout layer in the input and combines the two representations to make the final prediction. The authors utilized L1 regularization for the latent layer, enforcing sparsity on the activity of the latent representations. The Activation function used is leaky relu for all layers except the output layer, which uses tanh activation.

The first variation of this model (**DCS modified v1**) is identical to the originally proposed model, with the only modification of removing the direct connection with the dropout layer in the input. In the second model variation (**DCS modified v2**), we also removed the direct connection with the dropout layer in the input. An important modification is made in the training loss of this model. We include a distance term in the loss, to minimize the distance of latent embeddings coming from the same condition, regardless of the cell or species they are derived from. Finally, for these paired conditions we also minimize the mean squared error of predicted translated gene expression and the ground truth. The final variation (**DCS modified v3**) is identical to version 2, with the only difference of not calculating the mean squared error of direct translation when using the model. We still use a distance term in the loss function.

### TransCompR-based method

“Translatable Components Regression”^72^ (TransCompR) is a method, developed by Brubaker et al., that can map human data into the principal component space of another species to identify translatable animal features that can predict human disease processes and phenotypes. For translating molecular profiles, we use this framework for projecting the molecular profile of a biological system or species into the principal component space of another system or species. This principal component space is now equivalent to the latent space which can be used by a neural network (like the decoder) or a simple multi-linear regression model to predict the translated molecular profile (Supplementary Fig. 2).

### FIT-based method

FIT^15^ is a machine learning method that fits a linear regression model between homolog genes coming from the same perturbation tested on two different species (or it can be used with cell lines). During fitting a regularization penalty is added to force the slope of the fitted line to be 1 and the intercept 0. This trained framework can be used then to translate molecular profiles.

### Gene set enrichment analysis (GSEA)

Gene Set Enrichment Analysis (GSEA) was performed on multiple gene sets (Supplementary Fig. 3) using the FGSEA library^73,74^ from the Bioconductor resource^75^. Thus, the gene-level feature vector of each perturbation was transformed into a gene set-level feature vector of Normalized Enrichment Scores (NES).

### GSEA-based distance of transcriptomic profiles

The pairwise distance between gene expression feature vectors was calculated using the R package Gene Expression Signature in Bioconductor^76^, similar to Iorio et al. ^7^ Given two gene expression vectors ranked by their z-scored expression, A and B, GSEA is used to calculate the ES of the top and bottom genes of A in B and vice versa. The distance between the gene expression profiles is computed as

20 $$1-\frac{E{S}_{AinB}+E{S}_{BinA}}{2}$$

and ranges from 0 to 2. A GSEA distance equal to 0 means that the most upregulated and downregulated genes are the same in the two vectors A and B, while a distance equal to 2 means they are reversed. The GSEA distance is calculated for multiple thresholds as to how many top and bottom genes to consider and the average distance is taken for further analysis.

### Inference of transcription factor activity

To infer the transcription factor activity, we utilized the VIPER algorithm^77^ together with the Dorothea Regulon^78^. The VIPER algorithm calculates the enrichment of gene expression signatures of regulons, that are based on transcription regulatory networks. This way the activity of a transcription factor (TF) is inferred based on the expression of downstream genes known to be regulated by this specific TF. The Dorothea regulon contains known regulatory interactions, annotated based on the confidence that this interaction exists. Here, interactions are restricted to confidence levels A and B.

### Hardware and software specifications

All models were expressed in and trained using the PyTorch framework^79^ (version 1.12) in Python (version 3.8.8). When using the 978 landmark genes and for the serology case study, the models were trained in an NVIDIA GeForce RTX 3060 Laptop GPU with 6 GB of memory. The larger models (using 10,086 genes and the single-cell lung fibrosis data) were trained on the MIT Satori GPU cluster using NVIDIA V100 32GB memory GPU cards. Pre-processing and statistical analysis of the results were done in the R programming language (version 4.1.2). Visualization of results was done mainly using *ggplot2*^80^. More information about the versions of each library used can be found in the GitHub provided in the Data and Code availability sections.

### Reporting summary

Further information on research design is available in the Nature Research Reporting Summary linked to this article.

## Supplementary information

Supplementary Information

Reporting summary

## Data Availability

The study did not produce any new experimental data. The L1000 dataset was accessed via clue.io. The single-cell RNA-sequence data from Strunz et al. ^36^ and Habermann et al. ^37^ can be found in the Gene Expression Omnibus under their respective accession numbers GSE141259 and GSE135893. The serology datasets were retrieved from Chung et al. ^40^, upon request, and Barouch et al. ^38^, upon request. All analyzed data that were used to train our models and produce all tables and figures are available at https://github.com/Lauffenburger-Lab/OmicTranslationBenchmark (corresponding Zenodo: https://zenodo.org/doi/10.5281/zenodo.10475298).

## Code availability

The code to generate and pre-process data, figures, and tables, as well as train models, is available at https://github.com/Lauffenburger-Lab/OmicTranslationBenchmark (corresponding Zenodo: https://zenodo.org/doi/10.5281/zenodo.10475298).

## References

[1] Mak, I. W., Evaniew, N. & Ghert, M. Lost in translation: animal models and clinical trials in cancer treatment. *Am. J. Transl. Res.* **6**, 114–118 (2014). 24489990 PMC3902221

[2] Brubaker, D. K. & Lauffenburger, D. A. Translating preclinical models to humans. *Science* **367**, 742–743 (2020). 32054749 10.1126/science.aay8086

[3] Rhrissorrakrai, K. et al. Understanding the limits of animal models as predictors of human biology: lessons learned from the sbv IMPROVER species translation challenge. *Bioinformatics* **31**, 471–483 (2015). 25236459 10.1093/bioinformatics/btu611 PMC4325540

[4] Shay, T. et al. Conservation and divergence in the transcriptional programs of the human and mouse immune systems. *Proc. Natl Acad. Sci.* **110**, 2946–2951 (2013). 23382184 10.1073/pnas.1222738110 PMC3581886

[5] Gharib, W. H. & Robinson-Rechavi, M. When orthologs diverge between human and mouse. *Brief. Bioinform.* **12**, 436–441 (2011). 21677033 10.1093/bib/bbr031 PMC3178054

[6] Niepel, M. et al. Common and cell-type specific responses to anti-cancer drugs revealed by high throughput transcript profiling. *Nat. Commun.* **8**, 1186 (2017). 29084964 10.1038/s41467-017-01383-w PMC5662764

[7] Iorio, F. et al. Discovery of drug mode of action and drug repositioning from transcriptional responses. *PNAS* **107**, 14621–14626 (2010). 20679242 10.1073/pnas.1000138107 PMC2930479

[8] Iwata, M., Sawada, R., Iwata, H., Kotera, M. & Yamanishi, Y. Elucidating the modes of action for bioactive compounds in a cell-specific manner by large-scale chemically-induced transcriptomics. *Sci. Rep.* **7**, 40164 (2017). 28071740 10.1038/srep40164 PMC5223214

[9] Fotis, C., Meimetis, N., Sardis, A. & G. Alexopoulos, L. DeepSIBA: chemical structure-based inference of biological alterations using deep learning. *Mol. Omics* **17**, 108–120 (2021). 33188379 10.1039/d0mo00129e

[10] Lachmann, A. et al. Massive mining of publicly available RNA-seq data from human and mouse. *Nat. Commun.* **9**, 1366 (2018). 29636450 10.1038/s41467-018-03751-6 PMC5893633

[11] Wilks, C. et al. recount3: summaries and queries for large-scale RNA-seq expression and splicing. *Genome Biol.* **22**, 323 (2021). 34844637 10.1186/s13059-021-02533-6 PMC8628444

[12] Subramanian, A. et al. A next-generation connectivity map: L1000 platform and the first 1,000,000 profiles. *Cell* **171**, 1437–1452.e17 (2017). 29195078 10.1016/j.cell.2017.10.049 PMC5990023

[13] Poussin, C. et al. The species translation challenge—a systems biology perspective on human and rat bronchial epithelial cells. *Sci. Data* **1**, 140009 (2014). 25977767 10.1038/sdata.2014.9 PMC4322580

[14] Seok, J. Evidence-based translation for the genomic responses of murine models for the study of human immunity. *PLOS One* **10**, e0118017 (2015). 25680113 10.1371/journal.pone.0118017 PMC4332676

[15] Normand, R. et al. Found In Translation: a machine learning model for mouse-to-human inference. *Nat. Methods* **15**, 1067–1073 (2018). 30478323 10.1038/s41592-018-0214-9 PMC12396630

[16] Brubaker, D. K., Proctor, E. A., Haigis, K. M. & Lauffenburger, D. A. Computational translation of genomic responses from experimental model systems to humans. *PLOS Comput. Biol.* **15**, e1006286 (2019). 30629591 10.1371/journal.pcbi.1006286 PMC6343937

[17] Brubaker, D. K. et al. Proteogenomic network analysis of context-specific KRAS signaling in mouse-to-human cross-species translation. *Cell Syst.* **9**, 258–270.e6 (2019). 31521603 10.1016/j.cels.2019.07.006 PMC6816257

[18] Brubaker, D. K. et al. An interspecies translation model implicates integrin signaling in infliximab-resistant inflammatory bowel disease. *Sci. Signal.* **13**, eaay3258 (2020). 32753478 10.1126/scisignal.aay3258 PMC7459361

[19] Lee, M. J. et al. Computational interspecies translation between Alzheimer’s disease mouse models and human subjects identifies innate immune complement, TYROBP, and TAM receptor agonist signatures, distinct from influences of aging. *Front. Neurosci.* **15**, 727784 (2021). 34658769 10.3389/fnins.2021.727784 PMC8515135

[20] Schmidhuber, J. Deep learning in neural networks: an overview. *Neural Netw.* **61**, 85–117 (2015). 25462637 10.1016/j.neunet.2014.09.003

[21] Tan, J., Hammond, J. H., Hogan, D. A. & Greene, C. S. ADAGE-based integration of publicly available Pseudomonas aeruginosa gene expression data with denoising autoencoders illuminates microbe-host interactions. *mSystems* **1**, e00025–15 (2016). 27822512 10.1128/mSystems.00025-15 PMC5069748

[22] Wang, D. & Gu, J. VASC: dimension reduction and visualization of single-cell RNA-seq data by deep variational autoencoder. *Genom. Proteom. Bioinform.* **16**, 320–331 (2018). 10.1016/j.gpb.2018.08.003 PMC6364131 30576740

[23] Lopez, R., Regier, J., Cole, M. B., Jordan, M. I. & Yosef, N. Deep generative modeling for single-cell transcriptomics. *Nat. Methods* **15**, 1053–1058 (2018). 30504886 10.1038/s41592-018-0229-2 PMC6289068

[24] Eraslan, G., Simon, L. M., Mircea, M., Mueller, N. S. & Theis, F. J. Single-cell RNA-seq denoising using a deep count autoencoder. *Nat. Commun.* **10**, 390 (2019). 30674886 10.1038/s41467-018-07931-2 PMC6344535

[25] Lotfollahi, M., Wolf, F. A. & Theis, F. J. scGen predicts single-cell perturbation responses. *Nat. Methods* **16**, 715–721 (2019). 31363220 10.1038/s41592-019-0494-8

[26] Chen, L., Cai, C., Chen, V. & Lu, X. Learning a hierarchical representation of the yeast transcriptomic machinery using an autoencoder model. *BMC Bioinform.* **17**, S9 (2016). 10.1186/s12859-015-0852-1 PMC4895523 26818848

[27] Lotfollahi, M. et al. Biologically informed deep learning to query gene programs in single-cell atlases. *Nat. Cell Biol.* **25**, 337–350 (2023). 36732632 10.1038/s41556-022-01072-x PMC9928587

[28] Rampášek, L. et al. improving drug response prediction via modeling of drug perturbation effects. *Bioinformatics* **35**, 3743–3751 (2019). 30850846 10.1093/bioinformatics/btz158 PMC6761940

[29] Way, G. P. & Greene, C. S. Extracting a biologically relevant latent space from cancer transcriptomes with variational autoencoders. Pac. Symp. Biocomput. **2018**, 80–91 (World Scientific, 2017). 10.1142/9789813235533_0008. PMC5728678 29218871

[30] Xie, R., Wen, J., Quitadamo, A., Cheng, J. & Shi, X. A deep auto-encoder model for gene expression prediction. *BMC Genom.* **18**, 845 (2017). 10.1186/s12864-017-4226-0 PMC5773895 29219072

[31] Umarov, R., Li, Y. & Arner, E. DeepCellState: an autoencoder-based framework for predicting cell type specific transcriptional states induced by drug treatment. *PLOS Comput. Biol.* **17**, e1009465 (2021). 34610009 10.1371/journal.pcbi.1009465 PMC8519465

[32] Lotfollahi, M. et al. Predicting cellular responses to complex perturbations in high-throughput screens. *Mol. Syst. Biol.* **n/a**, e11517 (2023). 10.15252/msb.202211517 PMC10258562 37154091

[33] Escolano, C., Costa-jussà, M. R. & Fonollosa, J. A. R. (Self-Attentive) Autoencoder-based universal language representation for machine translation. Preprint at 10.48550/arXiv.1810.06351 (2018).

[34] Mohiuddin, T. & Joty, S. Unsupervised word translation with adversarial autoencoder. *Comput. Linguist.* **46**, 257–288 (2020).

[35] Sundararajan, M., Taly, A. & Yan, Q. Axiomatic attribution for deep networks. In *Proc. 34th International Conference on Machine Learning* 3319–3328 (PMLR, 2017).

[36] Strunz, M. et al. Alveolar regeneration through a Krt8+ transitional stem cell state that persists in human lung fibrosis. *Nat. Commun.* **11**, 3559 (2020). 32678092 10.1038/s41467-020-17358-3 PMC7366678

[37] Habermann, A. C. et al. Single-cell RNA sequencing reveals profibrotic roles of distinct epithelial and mesenchymal lineages in pulmonary fibrosis. *Sci. Adv.* **6**, eaba1972 (2020). 32832598 10.1126/sciadv.aba1972 PMC7439444

[38] Barouch, D. H. et al. Evaluation of a mosaic HIV-1 vaccine in a multicentre, randomised, double-blind, placebo-controlled, phase 1/2a clinical trial (APPROACH) and in rhesus monkeys (NHP 13-19). *Lancet* **392**, 232–243 (2018). 30047376 10.1016/S0140-6736(18)31364-3 PMC6192527

[39] Alter, G. et al. Passive transfer of vaccine-elicited antibodies protects against SIV in Rhesus Macaques. *Cell* **183**, 185–196.e14 (2020). 33007262 10.1016/j.cell.2020.08.033 PMC7534693

[40] Chung, A. W. et al. Dissecting polyclonal vaccine-induced humoral immunity against HIV using systems serology. *Cell* **163**, 988–998 (2015). 26544943 10.1016/j.cell.2015.10.027 PMC5490491

[41] Barretina, J. et al. The Cancer Cell Line Encyclopedia enables predictive modelling of anticancer drug sensitivity. *Nature* **483**, 603–607 (2012). 22460905 10.1038/nature11003 PMC3320027

[42] Courbebaisse, M. et al. VITamin D supplementation in renAL transplant recipients (VITALE): a prospective, multicentre, double-blind, randomized trial of vitamin D estimating the benefit and safety of vitamin D3 treatment at a dose of 100,000 UI compared with a dose of 12,000 UI in renal transplant recipients: study protocol for a double-blind, randomized, controlled trial. *Trials* **15**, 430 (2014). 25376735 10.1186/1745-6215-15-430 PMC4233037

[43] Wang, Y., Borchert, M. L. & DeLuca, H. F. Identification of the vitamin D receptor in various cells of the mouse kidney. *Kidney Int.* **81**, 993–1001 (2012). 22278022 10.1038/ki.2011.463 PMC3343313

[44] Yang, S. et al. Vitamin D receptor: a novel therapeutic target for kidney diseases. *Curr. Med. Chem.* **25**, 3256–3271 (2018). 29446731 10.2174/0929867325666180214122352 PMC6142412

[45] Grønbech, C. H. et al. scVAE: variational auto-encoders for single-cell gene expression data. *Bioinformatics* **36**, 4415–4422 (2020). 32415966 10.1093/bioinformatics/btaa293

[46] Tangherloni, A., Ricciuti, F., Besozzi, D., Liò, P. & Cvejic, A. Analysis of single-cell RNA sequencing data based on autoencoders. *BMC Bioinform.* **22**, 309 (2021). 10.1186/s12859-021-04150-3 PMC8186186 34103004

[47] Aran, D. et al. Reference-based analysis of lung single-cell sequencing reveals a transitional profibrotic macrophage. *Nat. Immunol.* **20**, 163–172 (2019). 30643263 10.1038/s41590-018-0276-y PMC6340744

[48] Ramachandran, P. et al. Resolving the fibrotic niche of human liver cirrhosis at single-cell level. *Nature* **575**, 512–518 (2019). 31597160 10.1038/s41586-019-1631-3 PMC6876711

[49] Haynes, B. F. et al. Immune-correlates analysis of an HIV-1 vaccine efficacy trial. *N. Engl. J. Med.* **366**, 1275–1286 (2012). 22475592 10.1056/NEJMoa1113425 PMC3371689

[50] Chung, A. W. & Alter, G. Systems serology: profiling vaccine-induced humoral immunity against HIV. *Retrovirology* **14**, 57 (2017). 29268769 10.1186/s12977-017-0380-3 PMC5740944

[51] Ching, T. et al. Opportunities and obstacles for deep learning in biology and medicine. *J. R. Soc. Interface* **15**, 20170387 (2018). 29618526 10.1098/rsif.2017.0387 PMC5938574

[52] Wysocka, M., Wysocki, O., Zufferey, M., Landers, D. & Freitas, A. A systematic review of biologically-informed deep learning models for cancer: fundamental trends for encoding and interpreting oncology data. *BMC Bioinform.* **24**, 198 (2023). 10.1186/s12859-023-05262-8 PMC10186658 37189058

[53] Wang, Q. & Breckon, T. P. Cross-domain structure preserving projection for heterogeneous domain adaptation. *Pattern Recognit.* **123**, 108362 (2022).

[54] Park, Y., Muttray, N. P. & Hauschild, A.-C. Species-agnostic transfer learning for cross-species transcriptomics data integration without gene orthology. 2023.08.11.552752 Preprint at 10.1101/2023.08.11.552752 (2023). 10.1093/bib/bbae004 PMC10835749 38305455

[55] Kenton, J. D. M. W. C. & Toutanova, L. K. Bert: Pre-training of deep bidirectional transformers for language understanding. *Proceedings of naacL-HLT* **1**, 2 (2019).

[56] Vaswani, A. et al. Attention is all you need. in *Advances in neural information processing systems* 5998–6008 (2017).

[57] Theodoris, C. V. et al. Transfer learning enables predictions in network biology. *Nature* 1–9 (2023) 10.1038/s41586-023-06139-9. 10.1038/s41586-023-06139-9 PMC10949956 37258680

[58] [clue.io]. https://clue.io/.

[59] Ioffe, S. & Szegedy, C. Batch normalization: accelerating deep network training by reducing internal covariate shift. In: *Proc. 32nd International Conference on Machine Learning* 448–456 (PMLR, 2015).

[60] Rasamoelina, A. D., Adjailia, F. & Sinčák, P. A Review of Activation Function for Artificial Neural Network. In: *Proc. IEEE 18th World Symposium on Applied Machine Intelligence and Informatics (SAMI)* 281–286 (2020). 10.1109/SAMI48414.2020.9108717.

[61] Srivastava, N., Hinton, G., Krizhevsky, A., Sutskever, I. & Salakhutdinov, R. Dropout: a simple way to prevent neural networks from overfitting. *J. Mach. Learn. Res.* **15**, 1929–1958 (2014).

[62] Belghazi, M. I., Baratin, A., Rajeshwar, S., Ozair, S., Bengio, Y., Courville, A., & Hjelm, D. Mutual information neural estimation. International Conference on Machine Learning. *PMLR* **80**, 531–540 (2018)

[63] Hjelm, R. D., Fedorov, A., Lavoie-Marchildon, S., Grewal, K., Bachman, P., Trischler, A., & Bengio, Y. Learning deep representations by mutual information estimation and maximization. International Conference on Learning Representations (2019)

[64] Sun, F.Y., Hoffman, J., Verma, V. and Tang, J. InfoGraph: Unsupervised and Semi-supervised Graph-Level Representation Learning via Mutual Information Maximization. International Conference on Learning Representations. OpenReview. net (2020)

[65] Fotis, C. et al. DeepSNEM: Deep Signaling Network Embeddings for compound mechanism of action identification. 2021.11.29.470365. https://www.biorxiv.org/content/10.1101/2021.11.29.470365v1 (2021). 10.1101/2021.11.29.470365.

[66] Sawilowsky, S. New effect size rules of thumb. *J. Mod. Appl. Stat. Methods* **8**, 597–599 (2009).

[67] Cohen, J. Statistical Power Analysis for the Behavioral Sciences. (Routledge, 2013).

[68] Kokhlikyan, N. et al. Captum: a unified and generic model interpretability library for PyTorch. Preprint at 10.48550/arXiv.2009.07896 (2020).

[69] McConn, J. L. et al. Optimal dimensionality selection for independent component analysis of transcriptomic data. *BMC Bioinform.* **22**, 584 (2021). 10.1186/s12859-021-04497-7 PMC8653613 34879815

[70] Sastry, A. V. et al. Mining all publicly available expression data to compute dynamic microbial transcriptional regulatory networks. 2021.07.01.450581 Preprint at 10.1101/2021.07.01.450581 (2021).

[71] Torsten Hothorn, A. Z. Diagnostic checking in regression relationships. *R. N.* **2**, 7–10 (2002).

[72] An interspecies translation model implicates integrin signaling in infliximab-resistant inflammatory bowel disease | Science Signaling. https://www.science.org/doi/full/10.1126/scisignal.aay3258. 10.1126/scisignal.aay3258 PMC7459361 32753478

[73] Sergushichev, A. A. An algorithm for fast preranked gene set enrichment analysis using cumulative statistic calculation. 060012 Preprint at 10.1101/060012 (2016).

[74] Korotkevich, G. et al. Fast gene set enrichment analysis. 060012 Preprint at 10.1101/060012 (2021).

[75] Gentleman, R. C. et al. Bioconductor: open software development for computational biology and bioinformatics. *Genome Biol.* **5**, R80 (2004). 15461798 10.1186/gb-2004-5-10-r80 PMC545600

[76] Li, F. et al. GeneExpressionSignature: an R package for discovering functional connections using gene expression signatures. *OMICS J. Integr. Biol.* **17**, 116–118 (2013). 10.1089/omi.2012.0087 23374109

[77] Alvarez, M. J. et al. Functional characterization of somatic mutations in cancer using network-based inference of protein activity. *Nat. Genet.* **48**, 838–847 (2016). 27322546 10.1038/ng.3593 PMC5040167

[78] Garcia-Alonso, L., Holland, C. H., Ibrahim, M. M., Turei, D. & Saez-Rodriguez, J. Benchmark and integration of resources for the estimation of human transcription factor activities. *Genome Res.* **29**, 1363–1375 (2019). 31340985 10.1101/gr.240663.118 PMC6673718

[79] Paszke, A., Gross, S., Massa, F., Lerer, A., Bradbury, J., Chanan, G., Killeen, T., Lin, Z., Gimelshein, N., Antiga, L. & Desmaison, A. Pytorch: An imperative style, high-performance deep learning library. *Advances in Neural Information Processing Systems* **32**, (2019).

[80] Villanueva, R. A. M. & Chen, Z. J. ggplot2: elegant graphics for data analysis (2nd ed.). *Meas. Interdiscip. Res. Perspect.* **17**, 160–167 (2019).

