# Author Correction: AutoTransOP: translating omics signatures without orthologue requirements using deep learning

**DOI:** 10.1038/s41540-024-00456-z

**Published:** 2024-12-13

**Authors:** Nikolaos Meimetis, Krista M. Pullen, Daniel Y. Zhu, Avlant Nilsson, Trong Nghia Hoang, Sara Magliacane, Douglas A. Lauffenburger

**Affiliations:** 1 Department of Biological Engineering, Massachusetts Institute of Technology, Cambridge, MA 02139 USA; 2 Department of Biology and Biological Engineering, Chalmers University of Technology, Gothenburg, SE 41296 Sweden; 3 School of Electrical Engineering and Computer Science, Washington State University, Pullman, WA 99164-236 USA; 4 Institute of Informatics, University of Amsterdam, Amsterdam, The Netherlands; 5 MIT-IBM Watson AI Lab, Cambridge, MA 02139 USA

**Keywords:** Computational biology and bioinformatics, Computer modelling

Correction to: *npj Systems Biology and Applications* 10.1038/s41540-024-00341-9, published online 29 January 2024

In the original version of this Article, the values of the reported Pearson’s correlations (regarding only comparing predicted and true variances) in the boxplots in panels a and c of Figure 5, as well as Supplementary Figures 27 and 39, were incorrect. This is due to an error identified in the script evaluating the performance of the model in predicting the variance of the raw counts per gene in the lung fibrosis case study. The comparisons of the different approaches result in the same conclusions, and thus no changes occur in the main text. Panels a and c in Figure 5, and Supplementary Figures 27 and 39 have now been corrected. The corresponding figure legends are not affected. The code in the corresponding repository, previously provided, have also been corrected.

