# Supplementary material for: AutoTransOP: translating omics signatures without orthologue requirements using deep learning
Source: NPJ Syst Biol Appl. 2024 Jan 29;10:13. doi: 10.1038/s41540-024-00341-9 (PMC10825146; doi:10.1038/s41540-024-00341-9)
Supplement: Supplementary file 1 — Supplementary Information [file 41540_2024_341_MOESM1_ESM.pdf]

Supplementary Information

AutoTransOP: Translating Omics Signatures without Orthologue Requirements using Deep Learning

Nikolaos Meimetis[1], Krista M. Pullen[1], Daniel Y. Zhu[1], Avlant Nilsson[1,2], Trong Nghia Hoang[3], Sara Magliacane[4,5] and Douglas A. Lauffenburger[1]*
1) Department of Biological Engineering, Massachusetts Institute of Technology, Cambridge, MA 02139, USA
2) Department of Biology and Biological Engineering, Chalmers University of Technology, Gothenburg, SE 41296, Sweden
3) School of Electrical Engineering and Computer Science, Washington State University, Pullman, WA, 99164-4236, USA
4) Institute of Informatics, University of Amsterdam, Amsterdam, The Netherlands
5) MIT-IBM Watson AI Lab, Cambridge, MA 02139, USA
* Corresponding author, lauffen@mit.edu

Supplementary Figures

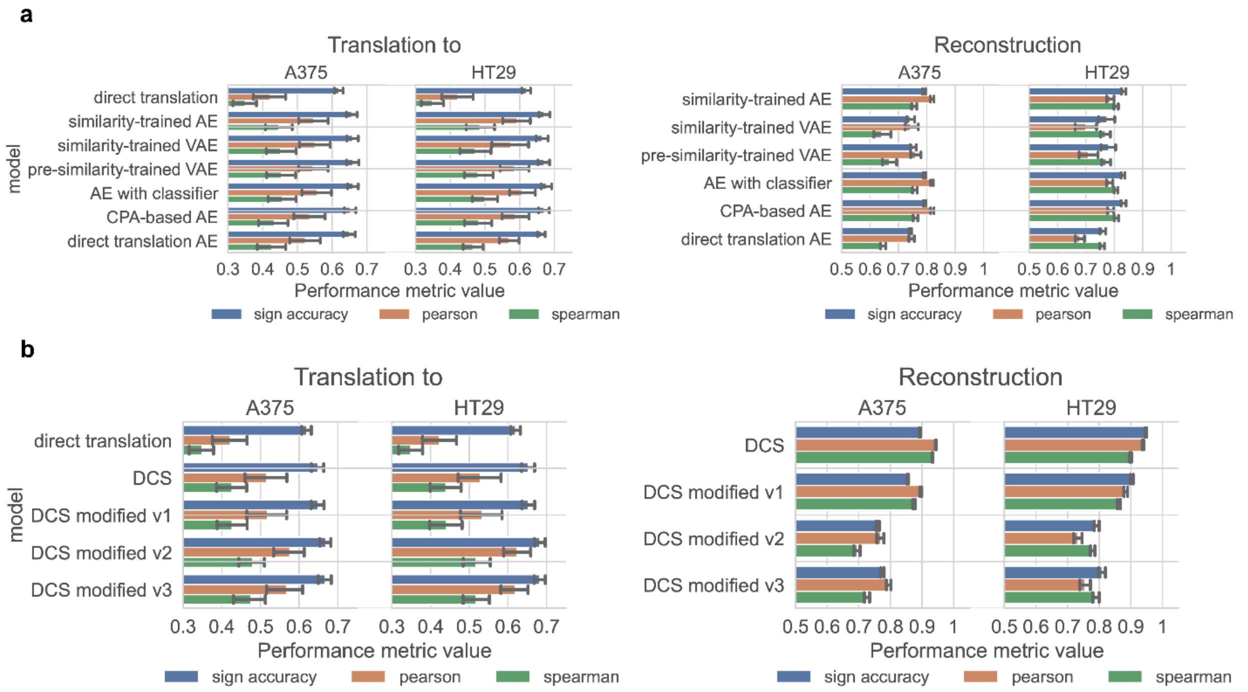

Supplementary Figure 1: **a)** Comparisons of variations of our framework when using the 10,086 genes of the L1000 dataset[1] **b)** Comparisons of variations of the DeepCellState[2] model when using the 10,086 genes of the L1000 dataset[1]. The error bars in the bar plots b-c denote 95% Confidence Intervals (CI).

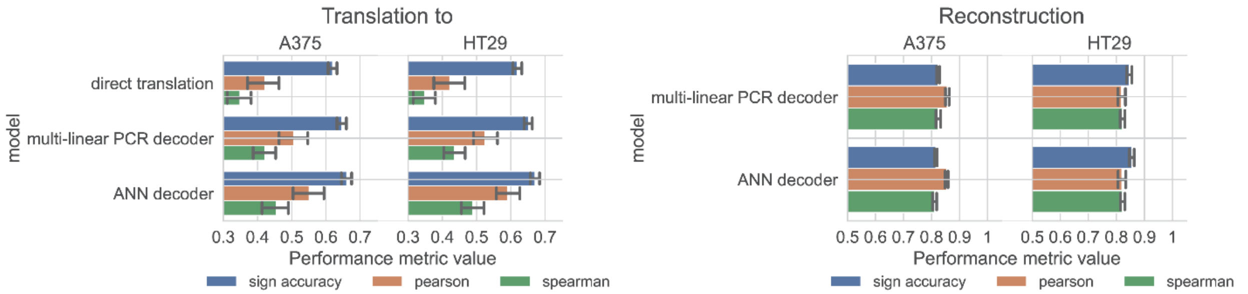

Supplementary Figure 2: Comparisons of variations of the TransCompR[3]-based model when using the 10,086 genes of the L1000 dataset[1]. The error bars in the bar plots b-c denote 95% Confidence Intervals (CI).

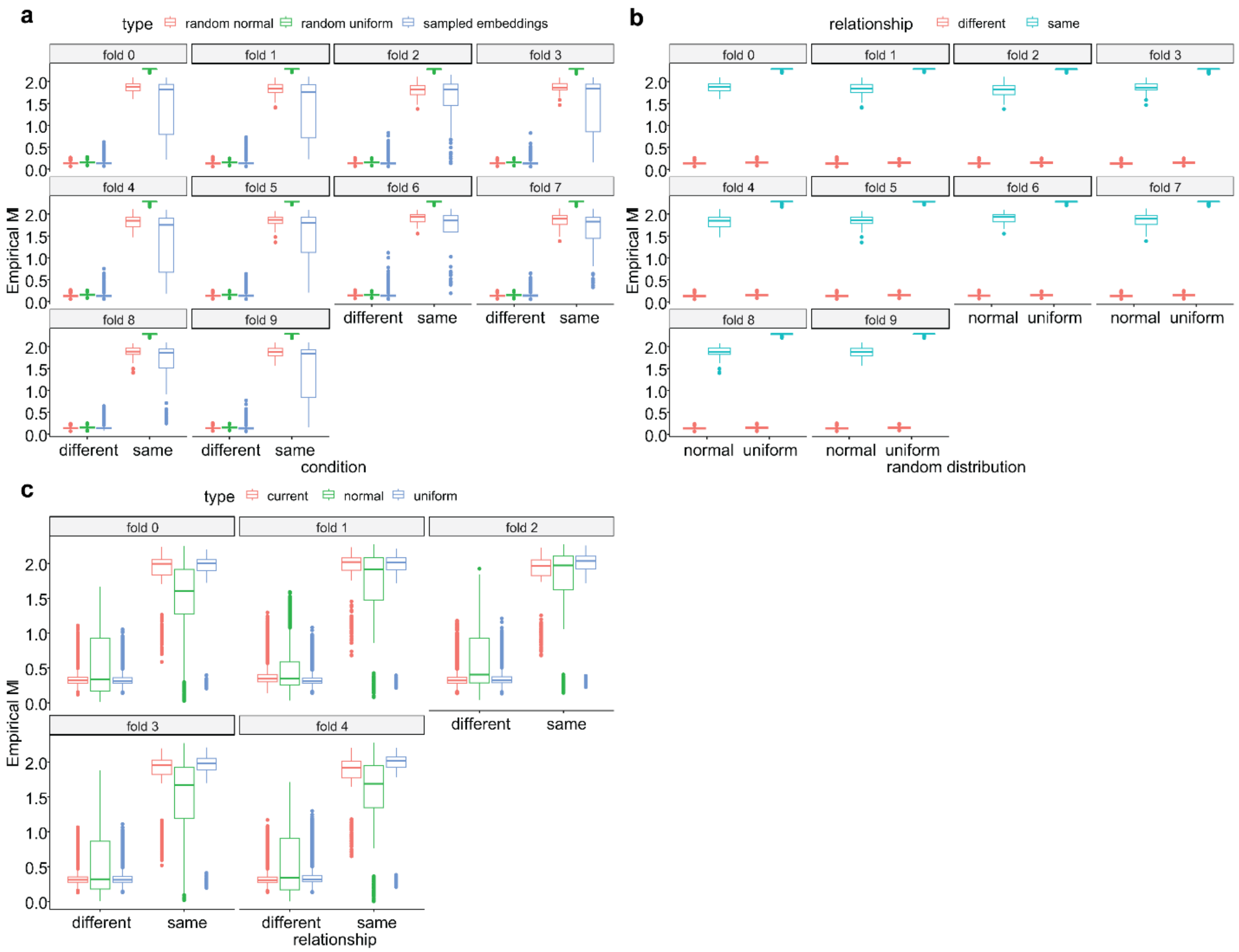

<u>Supplementary Figure 3:</u> a) Empirical mutual information (MI) distributions for synthetic random normal, random uniform, and our models' embeddings, in the cases of similar conditions and different conditions. **b)** Empirical MI distributions for synthetic random normal and random uniform embeddings, in the cases of similar conditions and different conditions. **c)** Empirical MI distributions model's embeddings that a different prior was enforced, in the cases of similar conditions and different conditions. In all boxplots, the centerline denotes the median, the bounds of the box denote the 1st and 3rd quantiles, and the whiskers denote points not being further from the median than 1.5 x interquartile range (IQR).

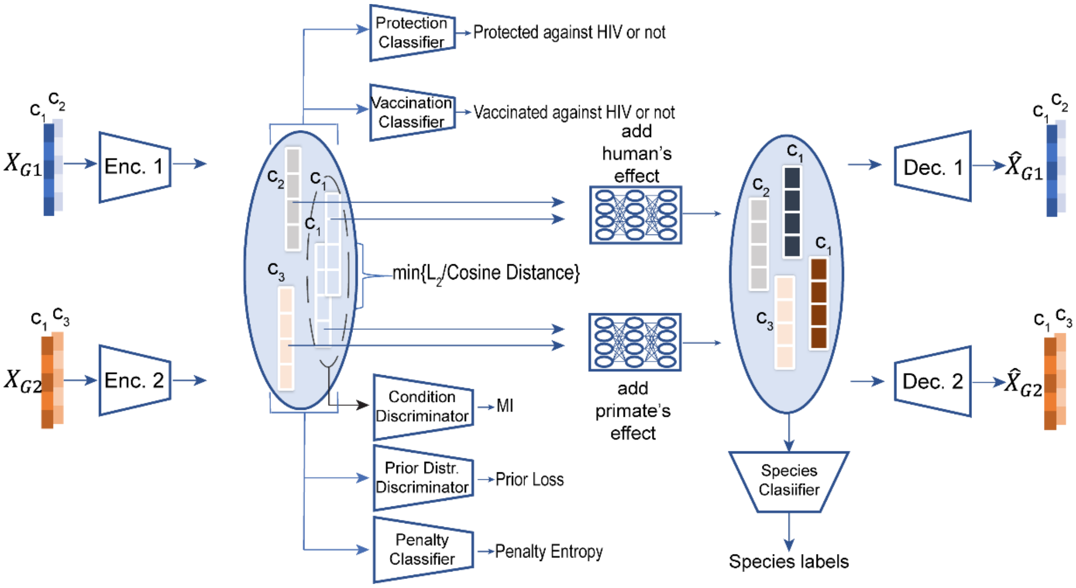

Supplementary Figure 4: Example of AutoTransOP v2 where the species effect is added with 2 small intermediate feedforward neural networks, instead of a trainable vector. The example is based on the serology case study where c_i, denotes a specific condition (e.g. human vaccinated and protected). The model is trained so that protected individuals are close, in the global latent space, regardless of species. While two separate classifiers try to predict vaccination status and protection in the global space, a third classifier predicts species in the composed latent space.

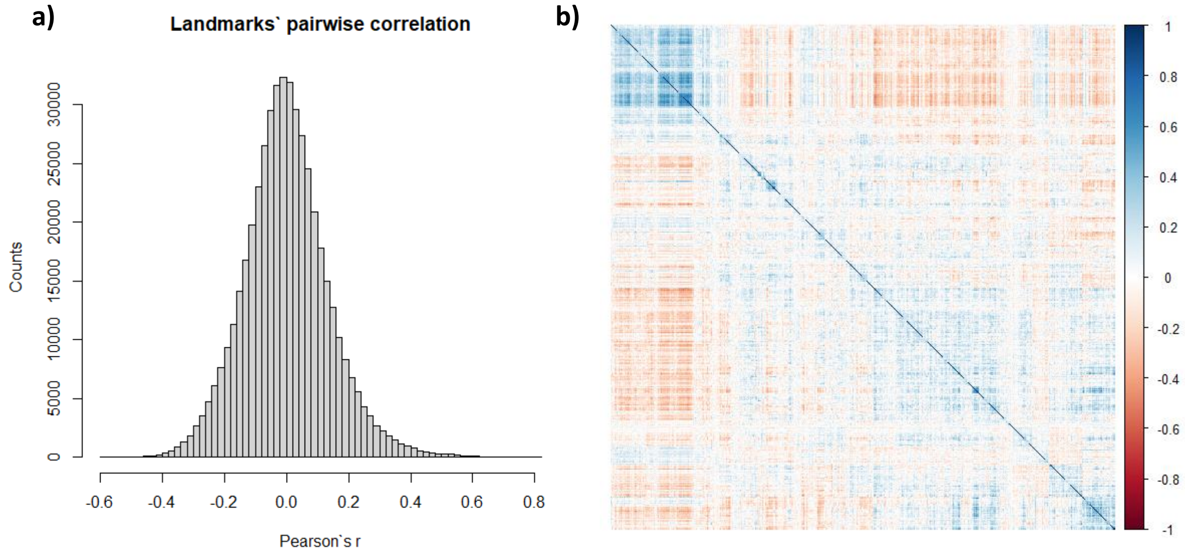

Supplementary Figure 5: a) Histogram of pairwise Pearson's correlation between landmark genes in the L1000 dataset[1]. **b)** Cluster map based on these correlations.

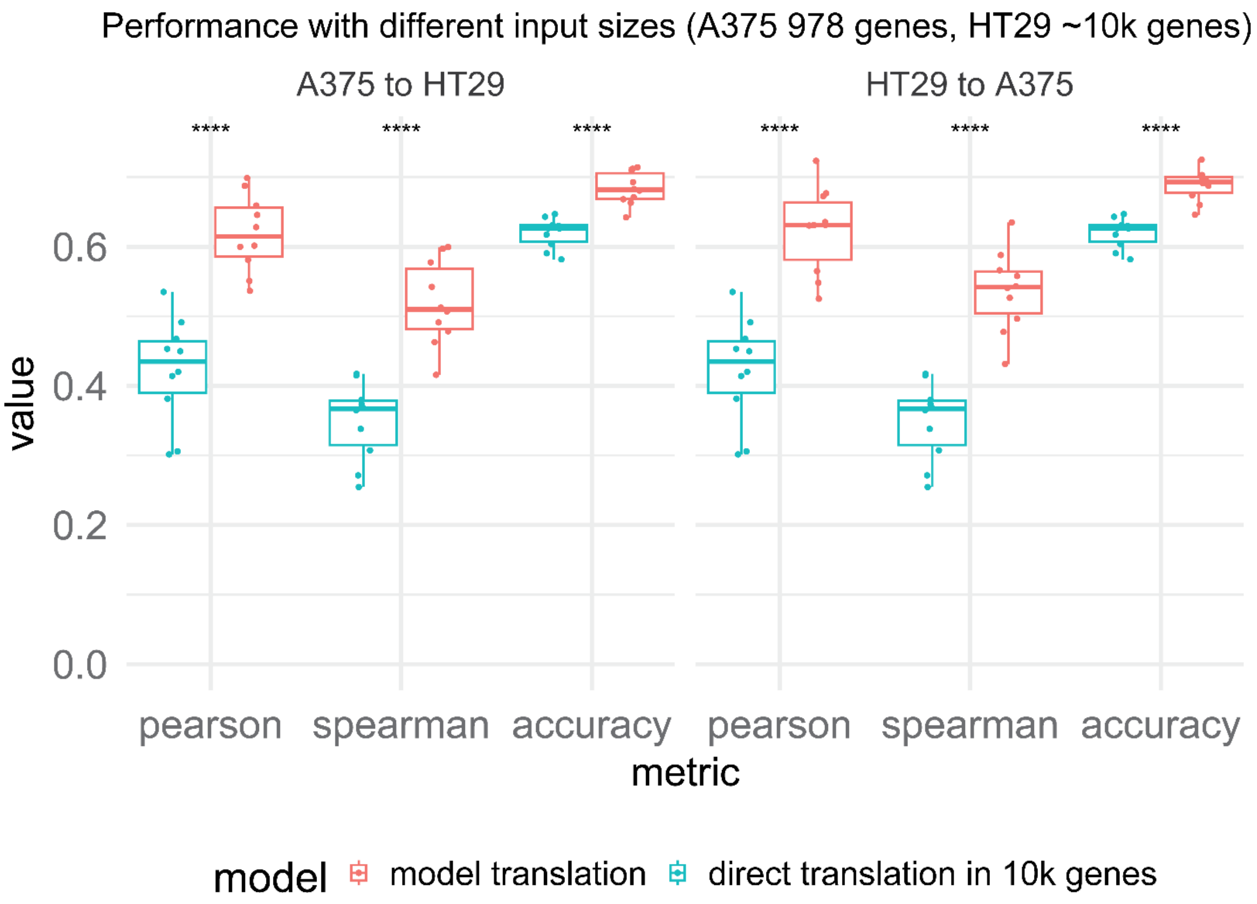

Supplementary Figure 6: Performance by using different inputs in the L1000 dataset[1]. In all boxplots, the centerline denotes the median, the bounds of the box denote the 1st and 3rd quantiles, and the whiskers denote points not being further from the median than 1.5 x interquartile range (IQR).

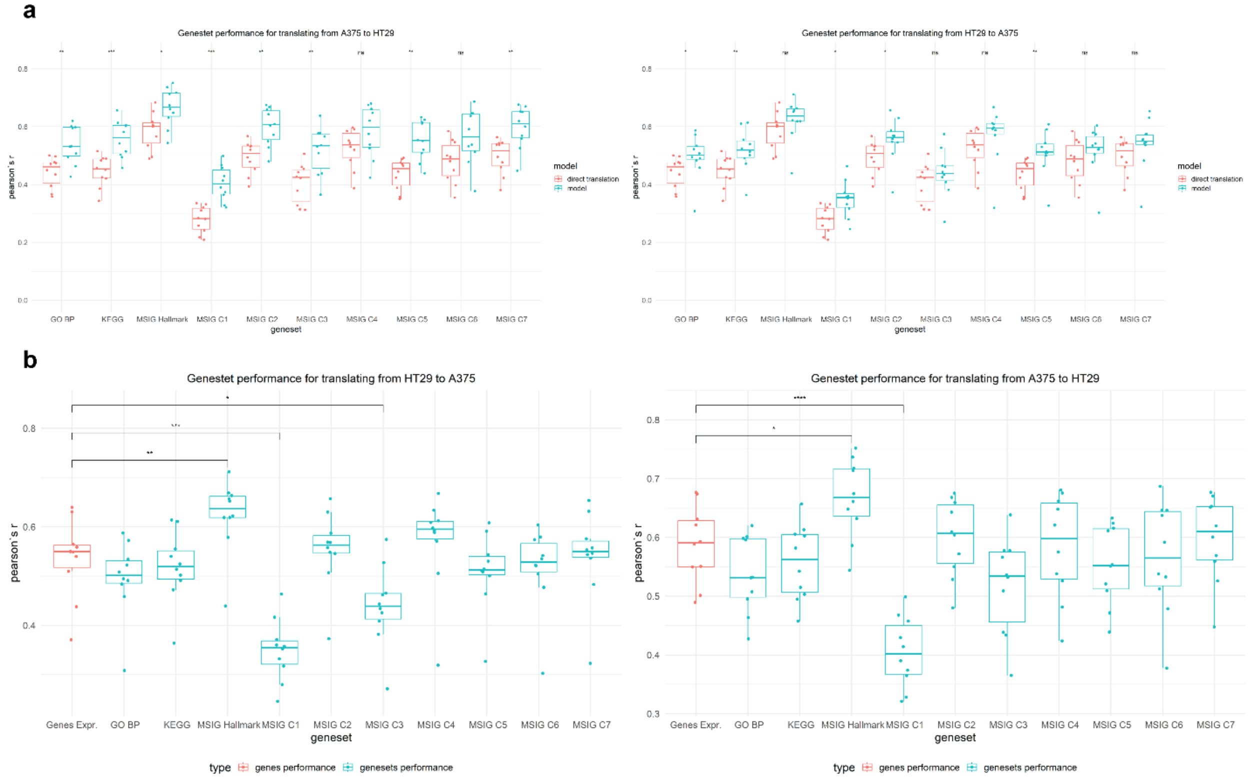

<u>Supplementary Figure 7:</u> Performance in inferring enrichment scores for different types of gene sets by using the predicted gene expression. **a)** Comparing the performance of predicted GSEA scores from translated signatures and the performance of direct translation of GSEA scores into another cell line. **b)** Comparison of GSEA scores performance and gene expression translation. For all comparisons in this figure, a two-sided Wilcoxon test was used with n=10 per group. In all boxplots, the centerline denotes the median, the bounds of the box denote the 1st and 3rd quantiles, and the whiskers denote points not being further from the median than 1.5 x interquartile range (IQR).

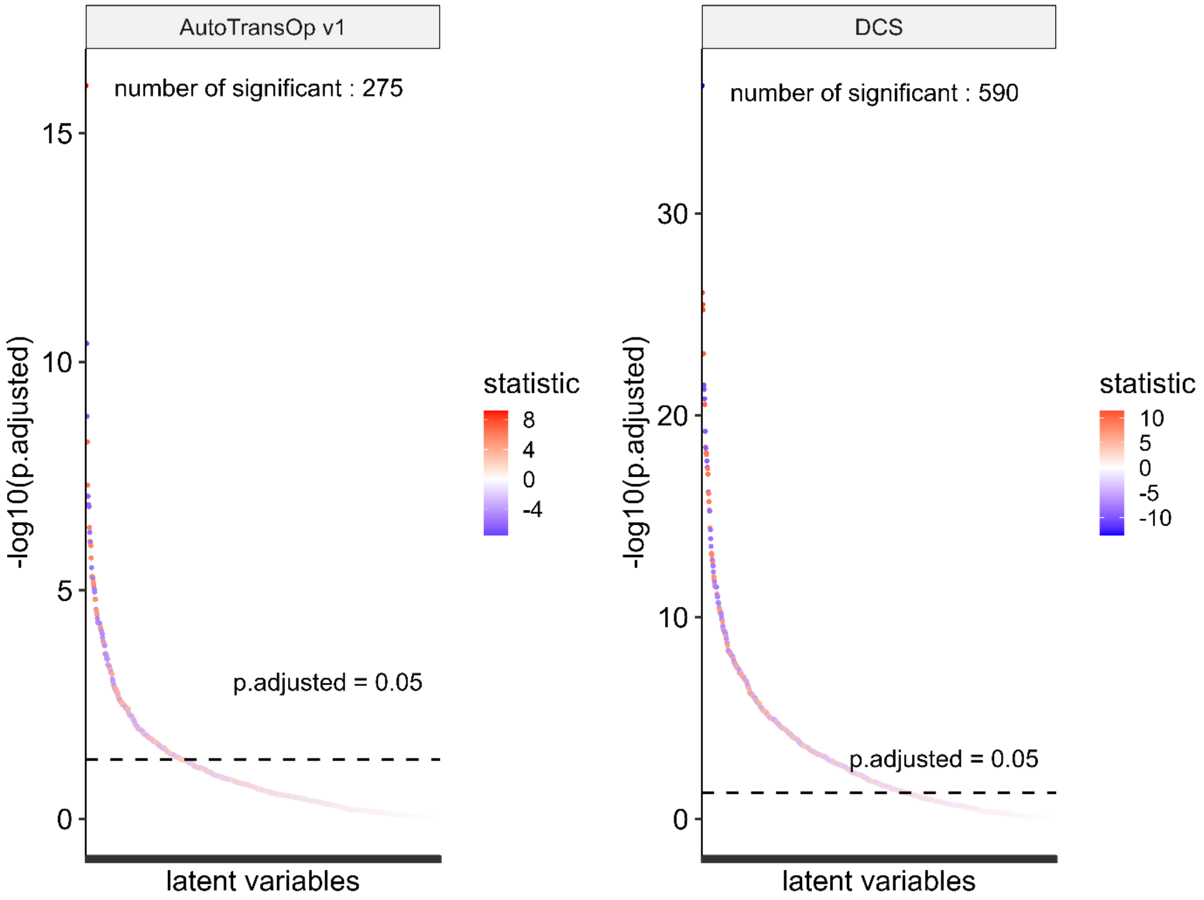

<u>**Supplementary Figure 8:**</u> Univariately significant latent variables for distinguishing between samples coming from A375 and HT29 cell lines. The value of the statistic metric is derived from a two-sided Student's t-test, while the p-value was adjusted for multiple hypothesis testing using Bonferroni correction.

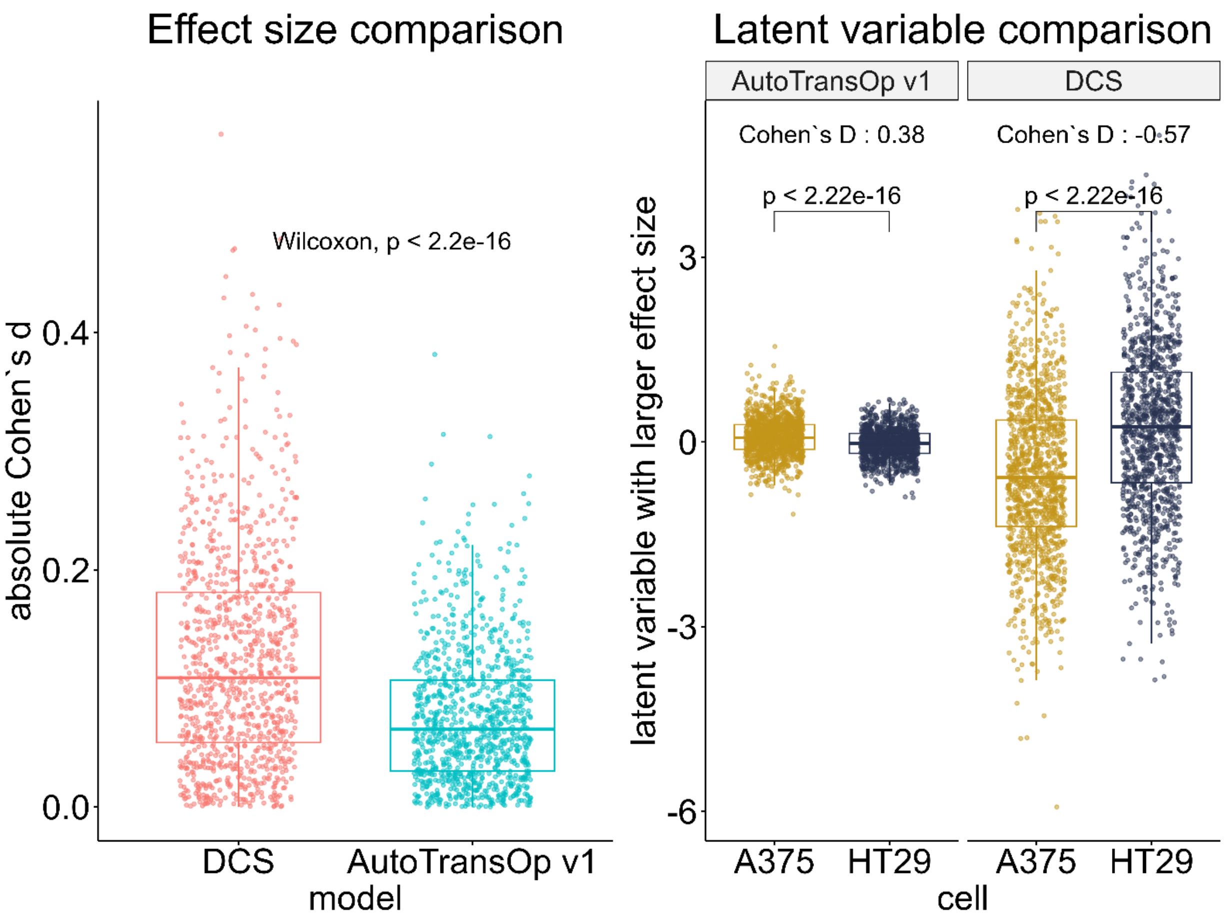

Supplementary Figure 9: Comparison between DeepCellState[2] and AutoTransOP v1, of the effect size between distributions of latent variables between A375 and HT29 cell lines. The effect size is calculated in terms of Cohen's d. In all boxplots, the centerline denotes the median, the bounds of the box denote the 1st and 3rd quantiles, and the whiskers denote points not being further from the median than 1.5 x interquartile range (IQR).

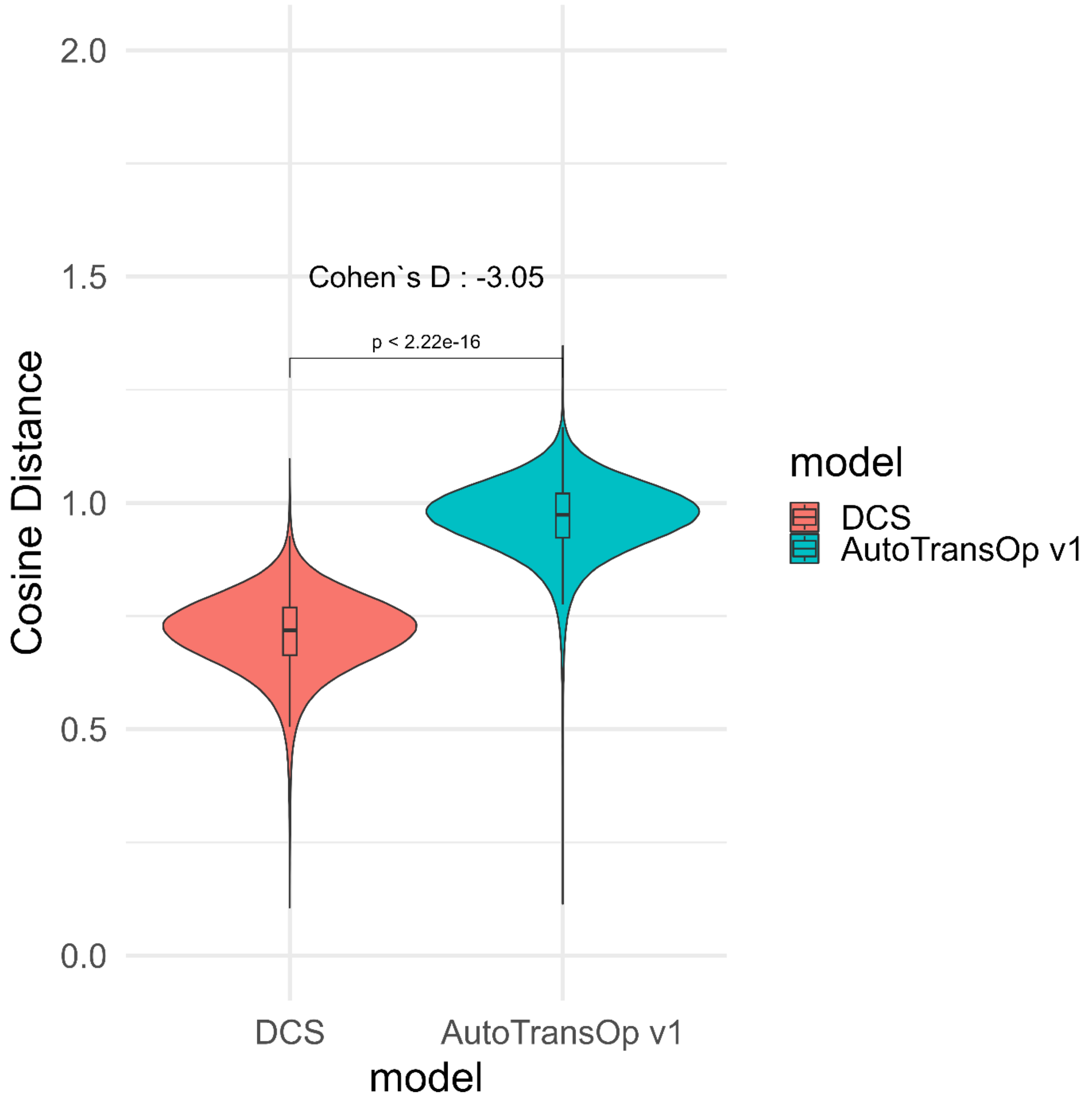

<u>Supplementary Figure 10:</u> Comparison of cosine distances, derived from pairs of embeddings coming from the same cell lines (A375 or HA1E), between DeepCellState[2] and AutoTransOP v1. In all boxplots, the centerline denotes the median, the bounds of the box denote the 1st and 3rd quantiles, and the whiskers denote points not being further from the median than 1.5 x interquartile range (IQR).

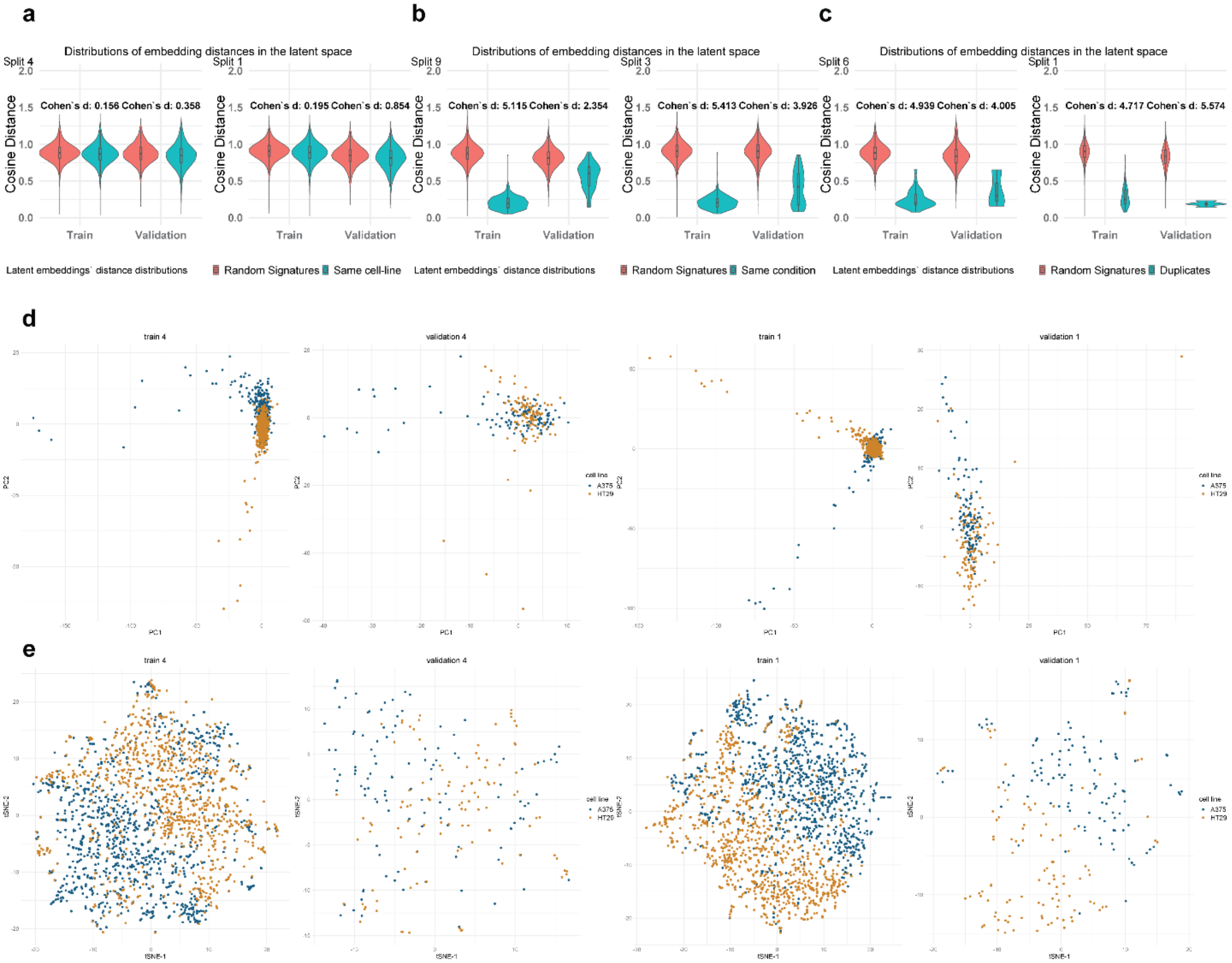

<u>Supplementary Figure 11:</u> The embeddings used here are derived from using all the 10,086 L1000 genes with one global latent space, with a jointly trained classifier. **a)** Cosine distance between embeddings coming from random pairs of signatures and pairs coming from the same cell line. **b)** Cosine distance between embeddings coming from random pairs of signatures and pairs coming from the same condition **c)** Cosine distance between embeddings coming from random pairs of signatures and pairs being biological duplicates **d)** PCA visualization for the splits with maximum and minimum separation **e)** t-SNE visualization for the splits with the maximum and minimum separation. In all boxplots, the centerline denotes the median, the bounds of the box denote the 1st and 3rd quantiles, and the whiskers denote points not being further from the median than 1.5 x interquartile range (IQR).

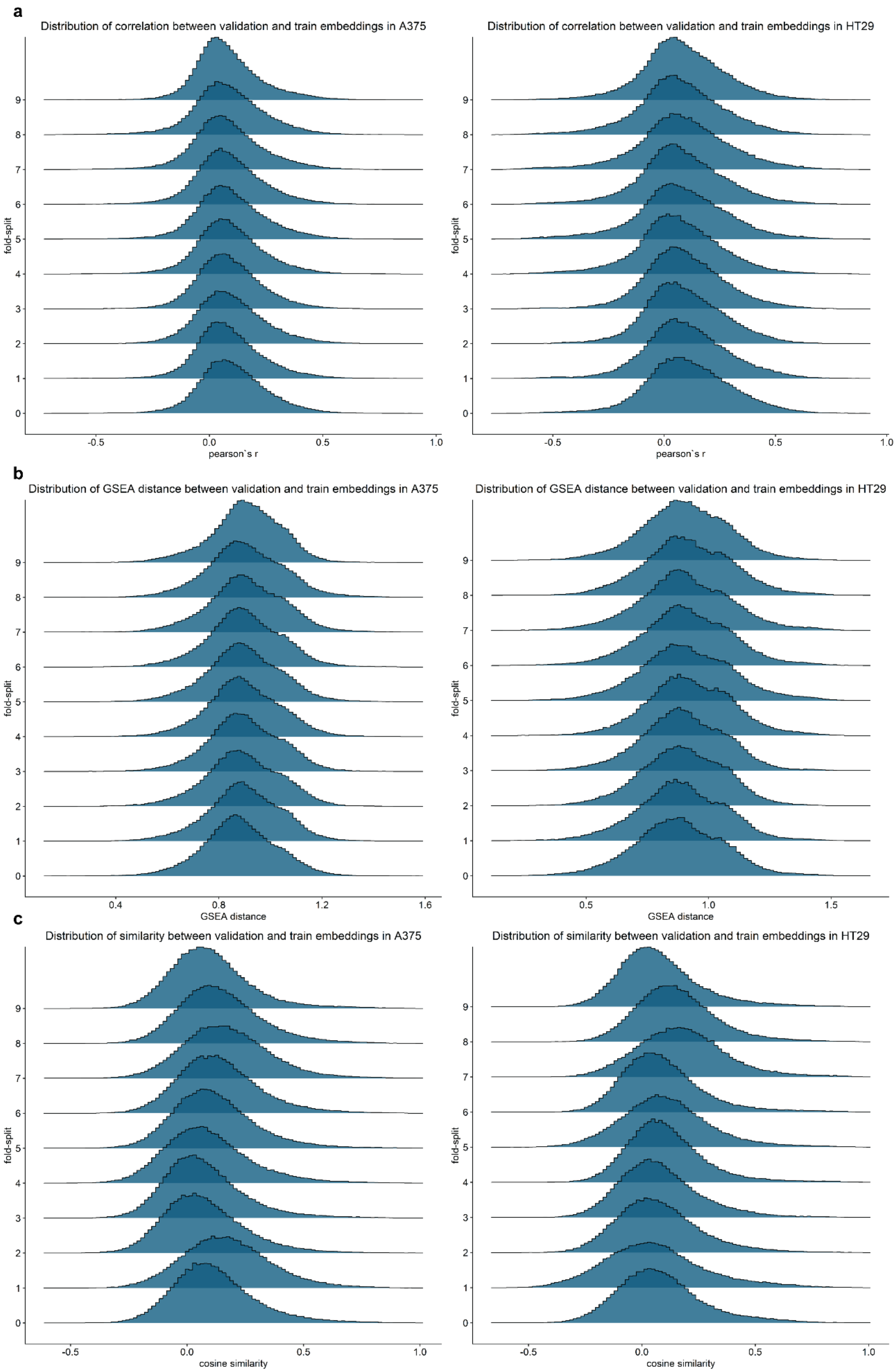

<u>Supplementary Figure 12:</u> Similarity of validation samples and training samples in 10-fold cross-validation. **a-b)** Gene expression correlation distribution, between every validation sample and training

sample across folds, in A375 and HT29. **c-d)** GSEA-based distance (see Supplementary Method 5) distribution between gene expression profiles of validation and train samples in A375 and HT29. **e-f)** Cosine similarity between latent space embeddings of validation and train samples in A375 and HT29

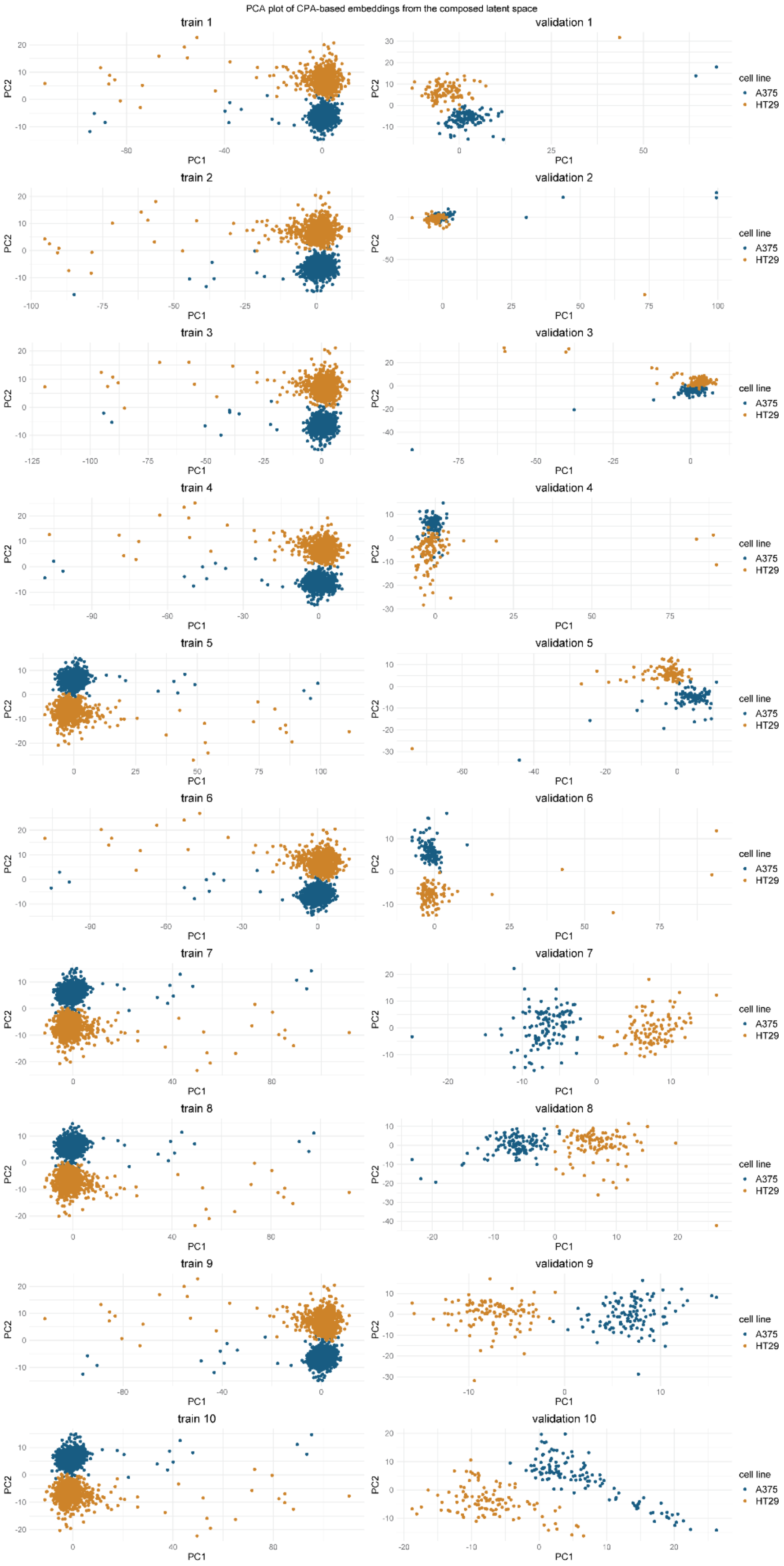

Supplementary Figure 13: Composed latent space PCA visualization for embeddings derived from the CPA-combined approach using 10,086 L1000 genes.

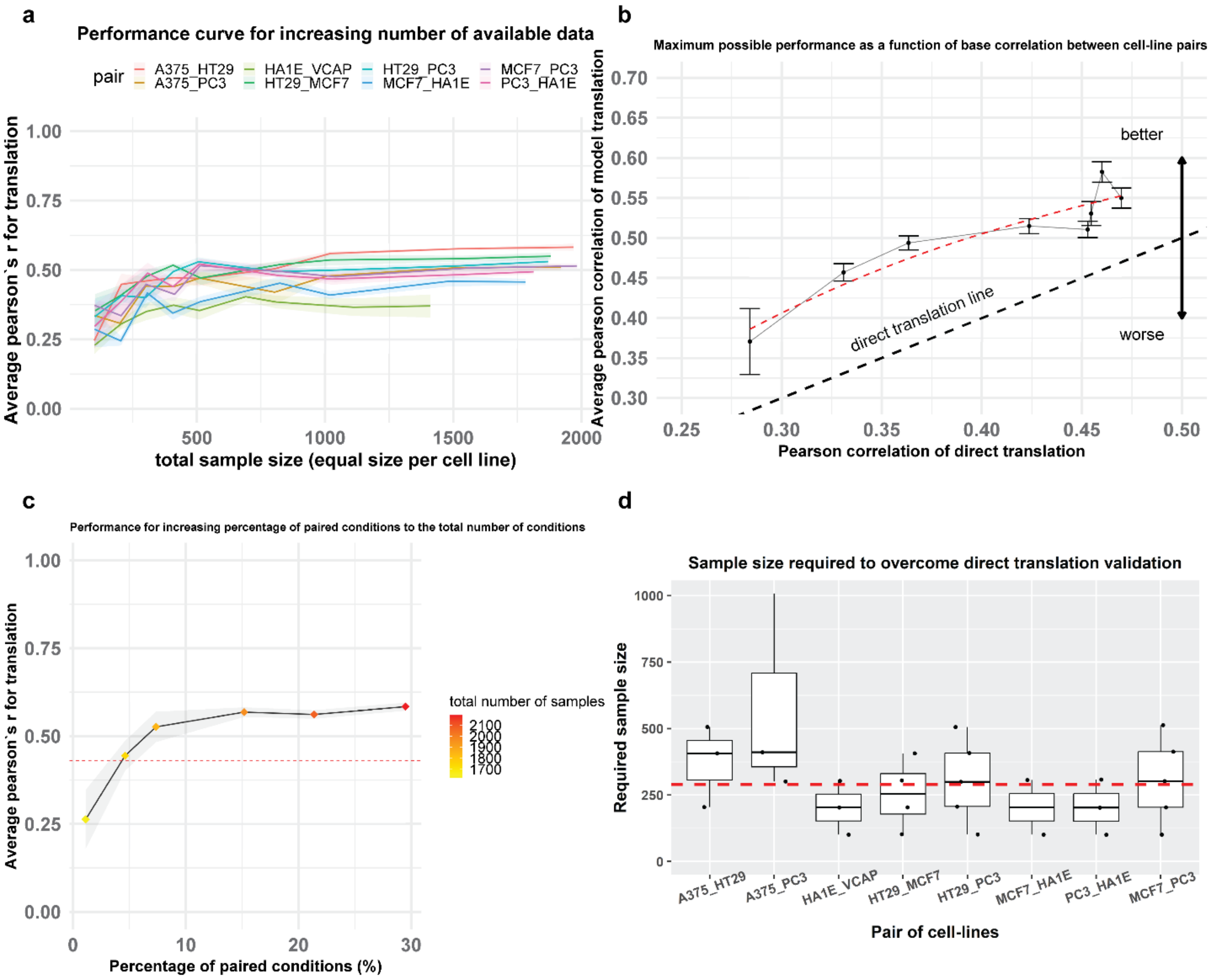

Supplementary Figure 14: Performance analysis using all the 10,086 genes for the L1000 **a)** Performance in the translation task of the CPA-combined approach across different cell-line pairs and different sizes of training data. **b)** Model performance in translation as a function of the initial similarity of 2 cell lines. All error bars in this figure denote a deviation of one Standard Error (SE) from the mean. **c)** Model performance in translation for different percentages of paired conditions. **d)** Required sample size to overcome direct translation's performance. In all boxplots, the centerline denotes the median, the bounds of the box denote the 1st and 3rd quantiles, and the whiskers denote points not being further from the median than 1.5 x interquartile range (IQR). All the shaded areas in this figure represent a deviation of one SE from the mean.

a

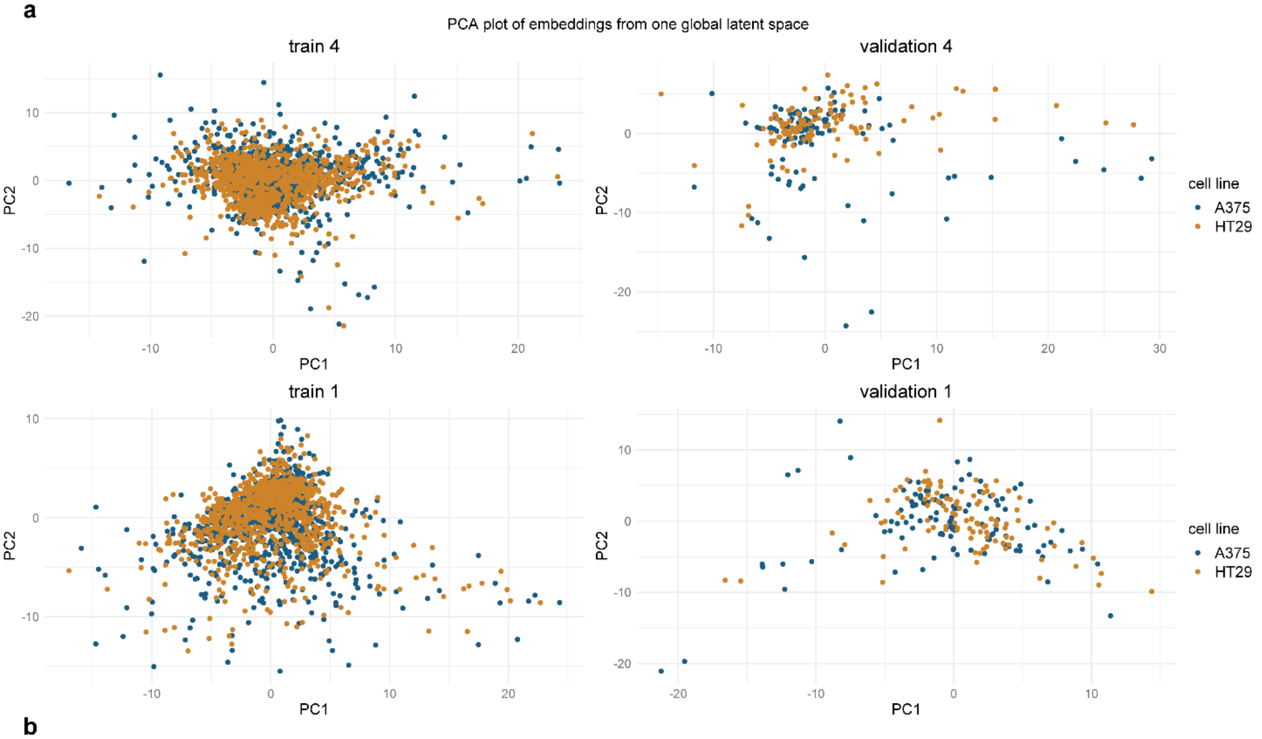

b

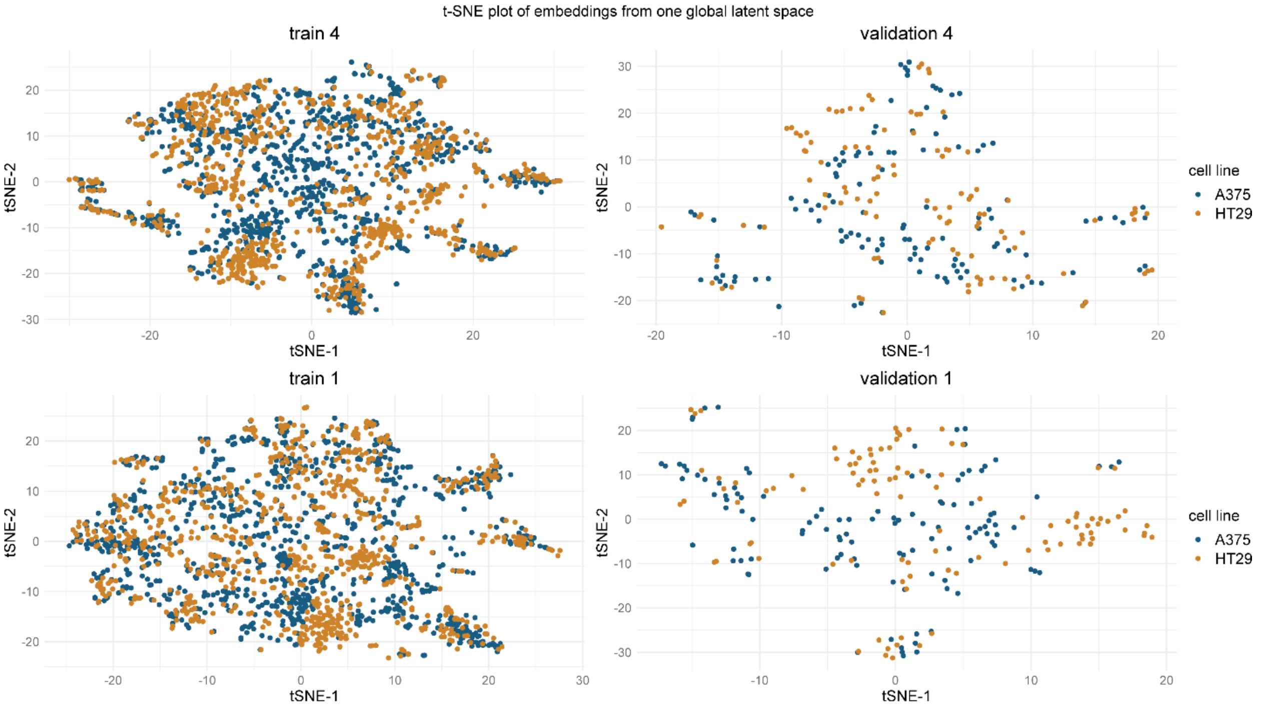

<u>Supplementary Figure 15:</u> The two splits in 10-fold cross-validation that are shown each time here are the ones where the maximum and minimum difference between the two distributions is observed. For every other split, the difference is between these two extreme cases. Latent space visualization for embeddings derived from the approach with one global latent space using the 978 landmark genes. **a)** PCA plot of global latent space embeddings **b)** t-SNE plot of global latent space embeddings.

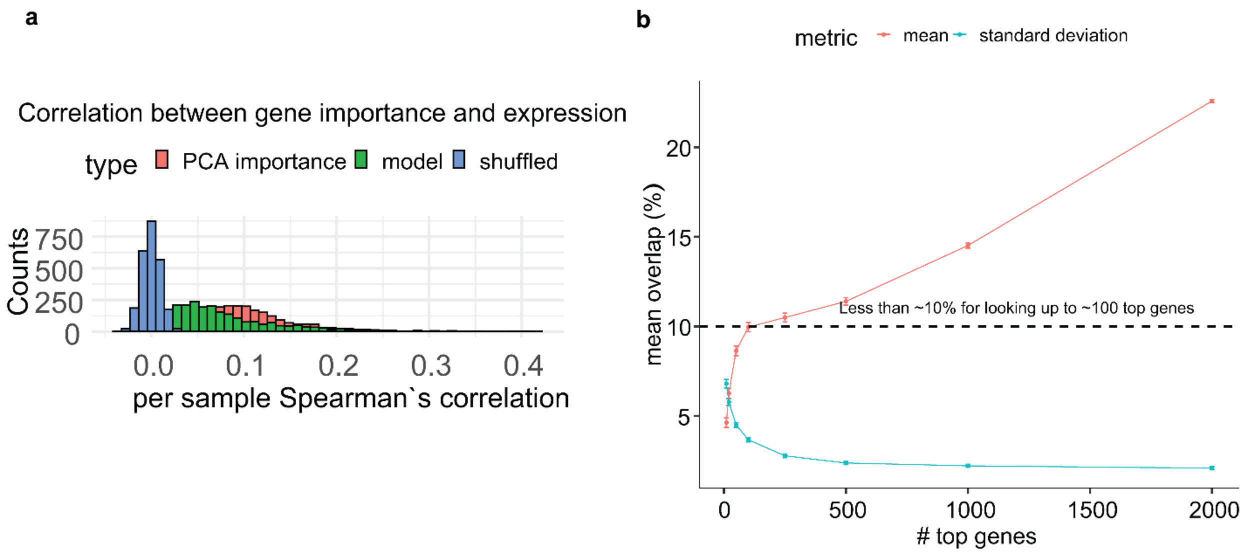

Supplementary Figure 16: **a)** Per sample Spearman's correlation between absolute gene importance scores and absolute gene expression. **b)** Mean and standard deviation of the overlap of top important and top regulated genes. The error bars denote the 99% Confidence Intervals (CI).

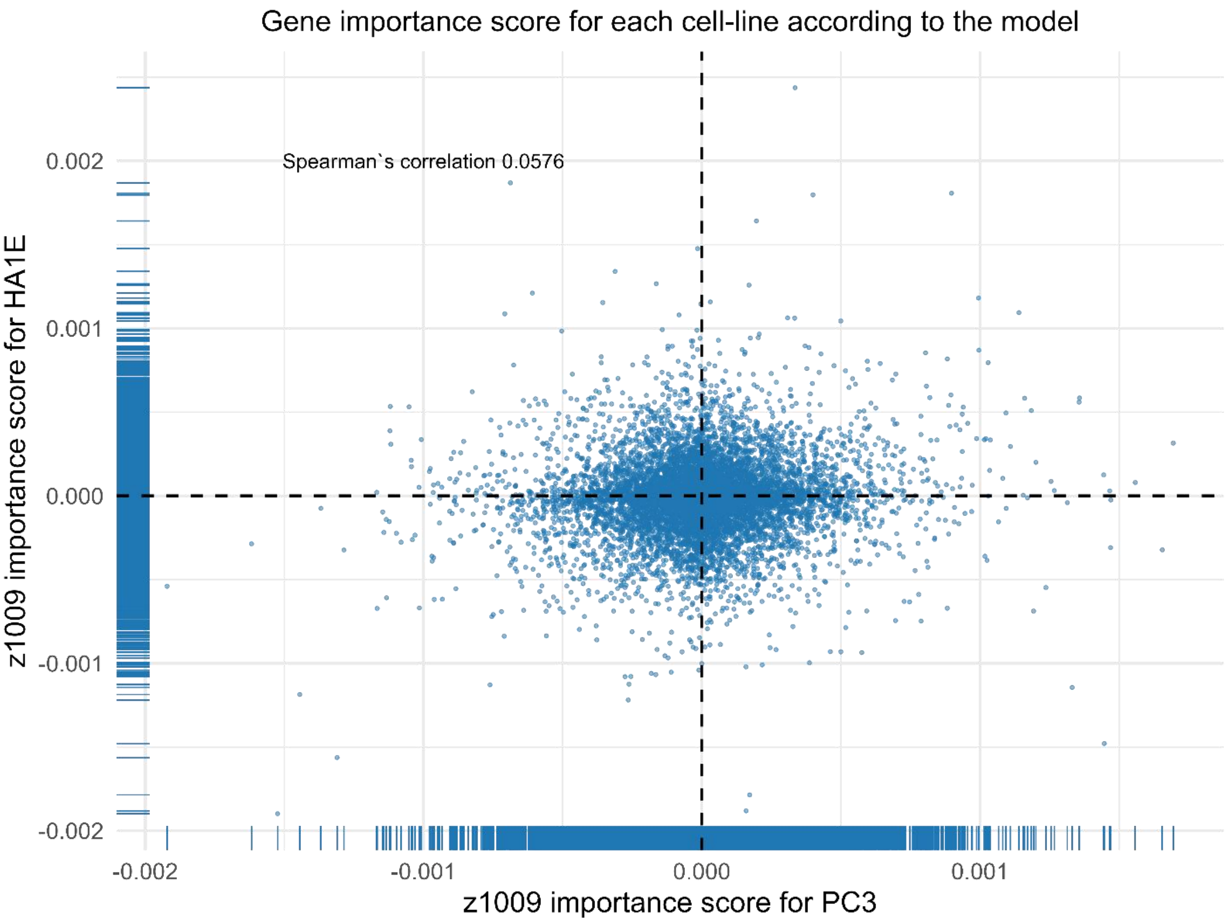

Supplementary Figure 17: Average importance scores of genes from PC3 to control cell-specific latent variables versus the importance scores from HA1E, according to the individual encoders.

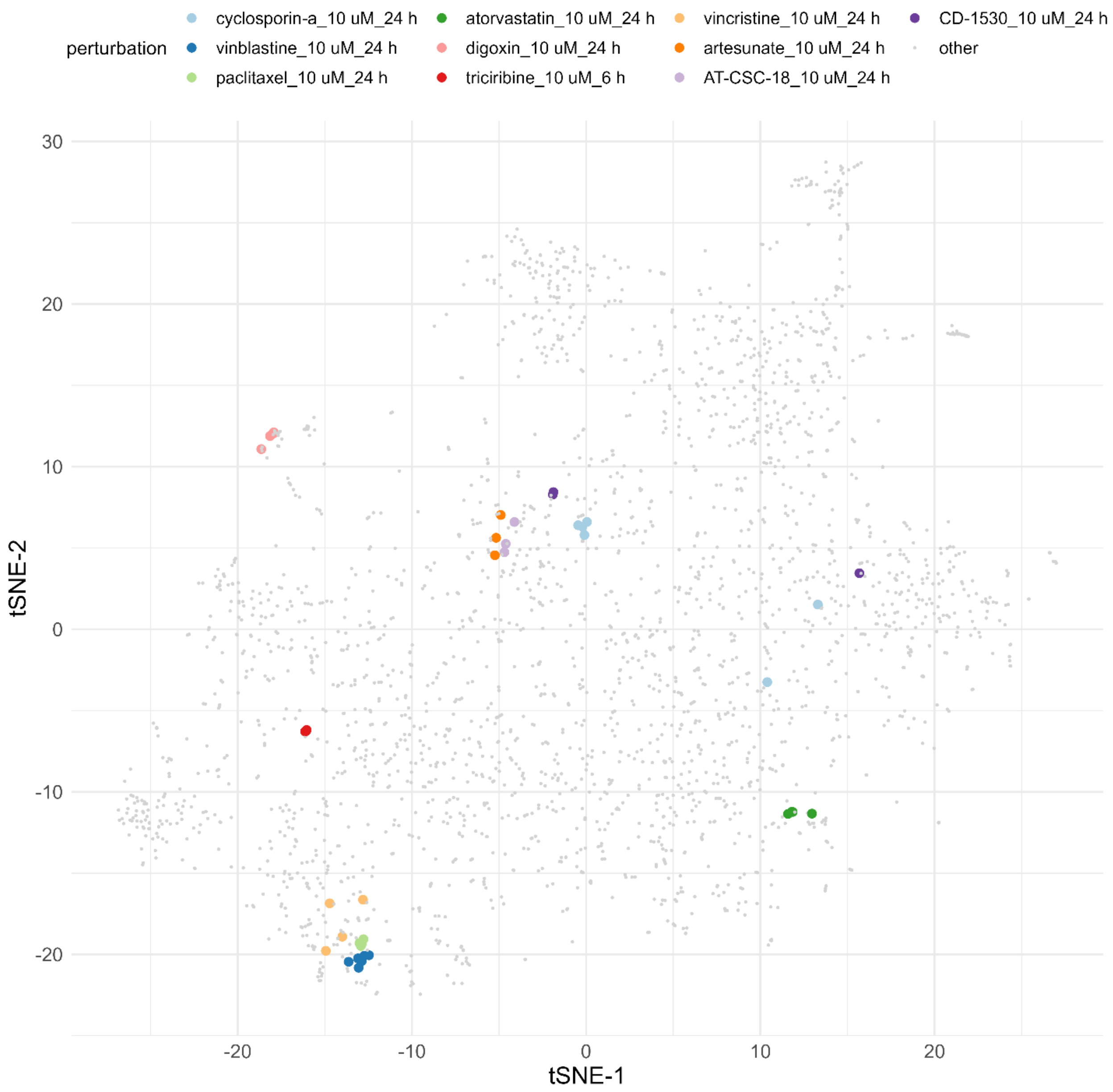

<u>Supplementary Figure 18:</u> t-SNE plot, visualizing embeddings coming from the same condition (drug + dose + time point), for the top 10 conditions in terms of number of samples, in the dataset used for translation between PC3 and HA1E cell lines.

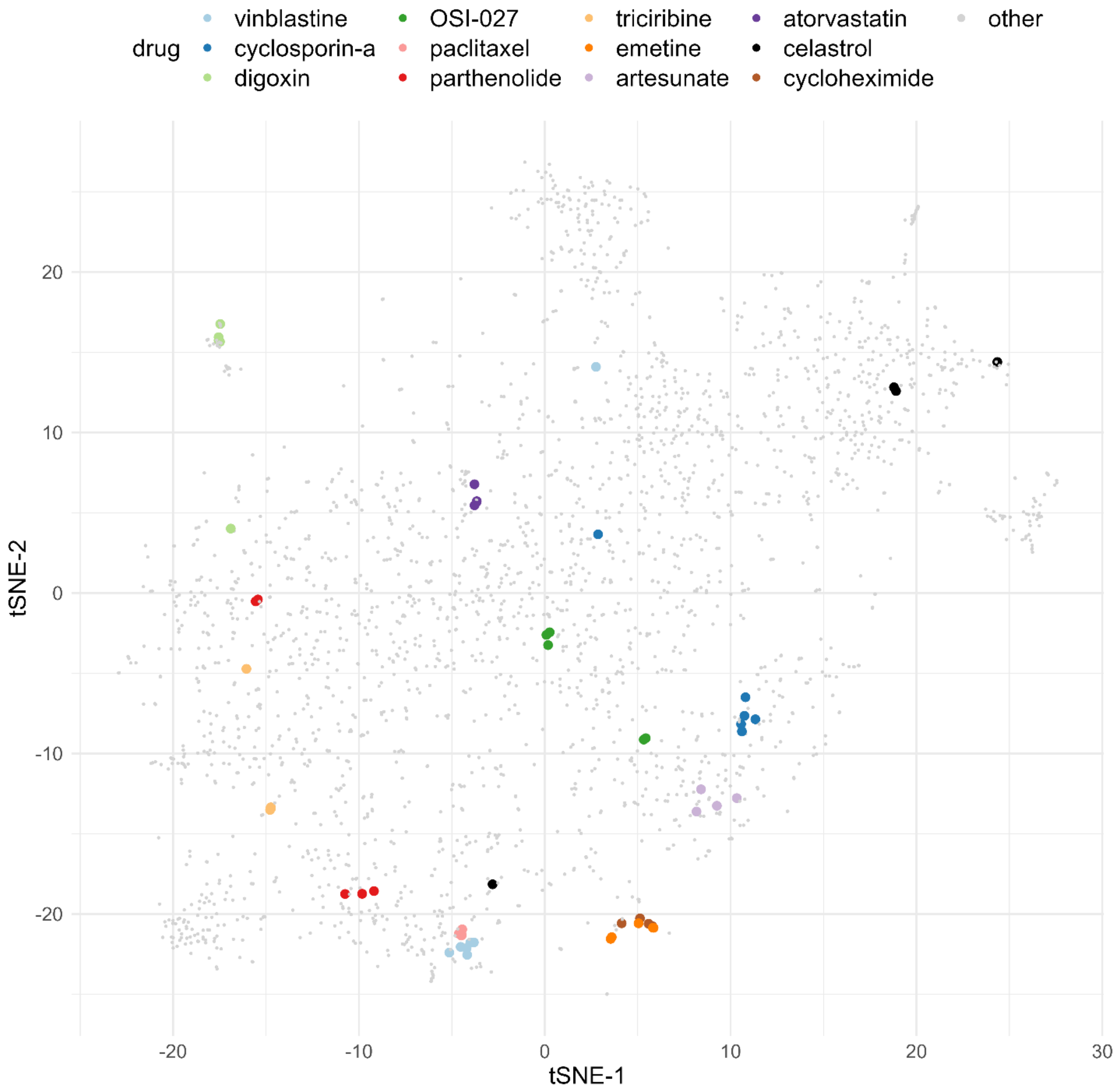

<u>Supplementary Figure 19:</u> t-SNE plot, visualizing embeddings coming from the same drug, for the top 13 drugs in terms of number of samples, in the dataset used for translation between PC3 and HA1E cell lines.

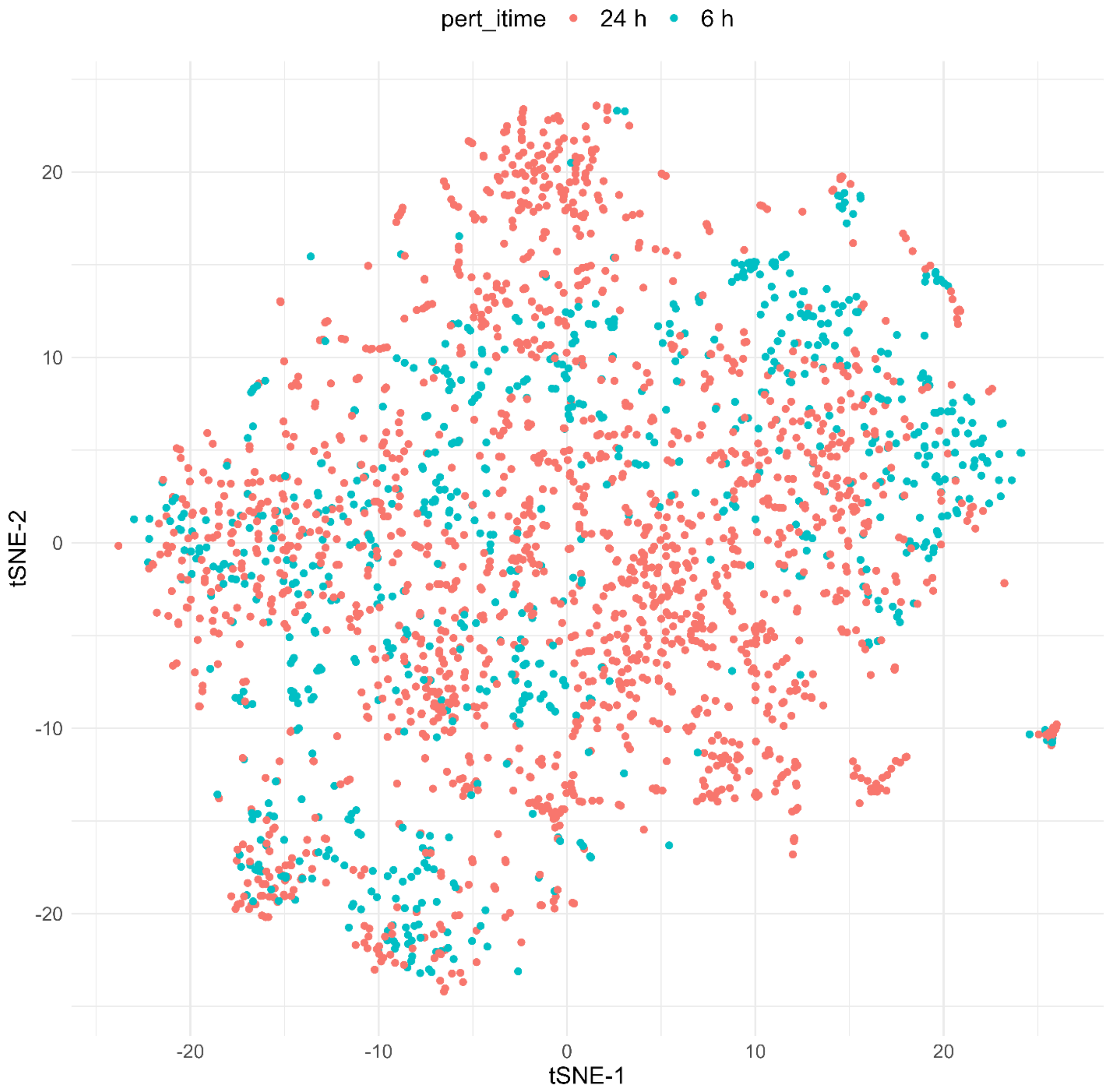

<u>Supplementary Figure 20:</u> t-SNE plot, visualizing embeddings coming from the time point, in the dataset used for translation between PC3 and HA1E cell lines.

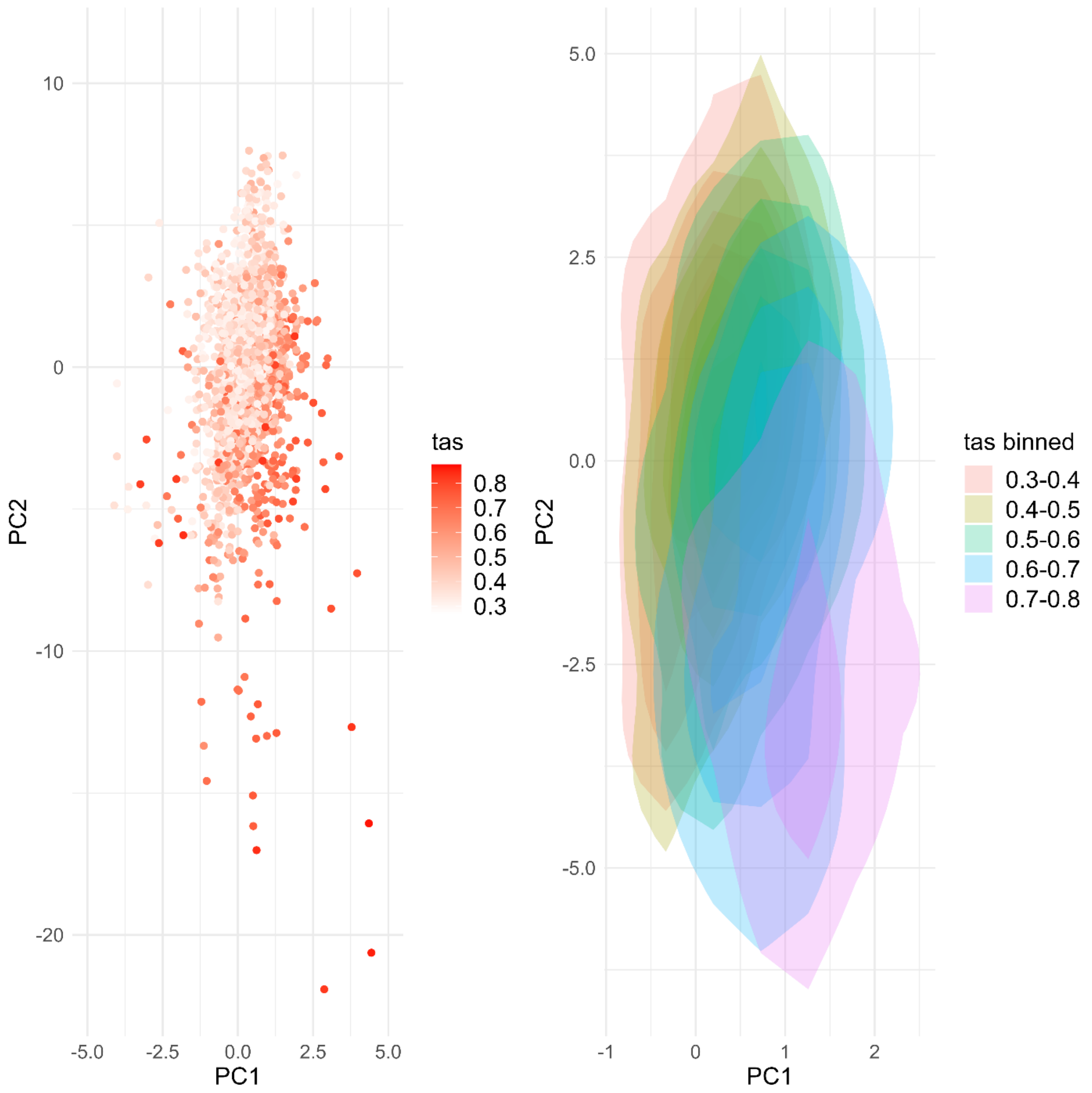

Supplementary Figure 21: PCA plot, visualizing the TAS distribution in the latent space for embeddings coming from the dataset used for translation between PC3 and HA1E cell lines.

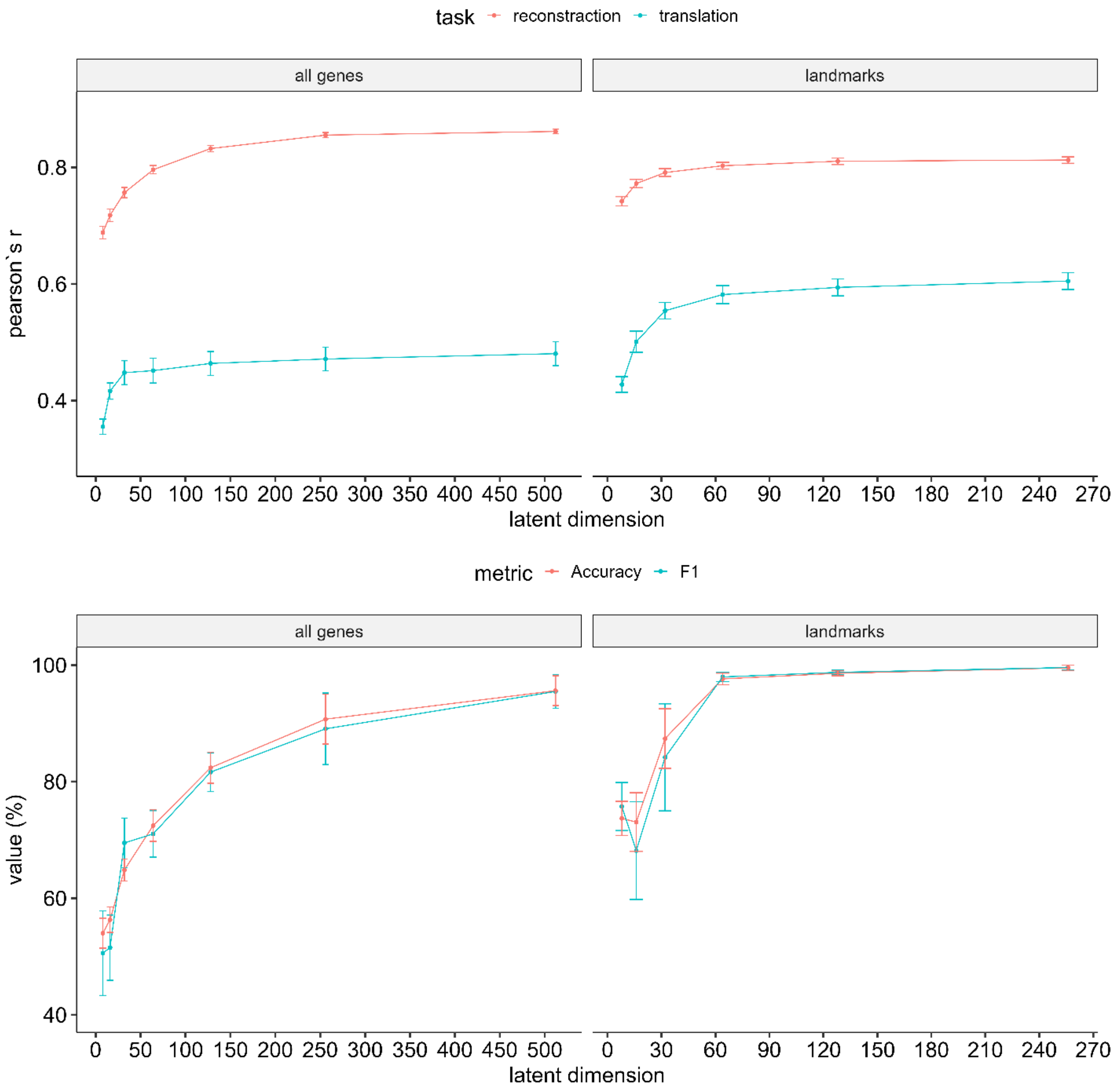

Supplementary Figure 22: Performance of the model in reconstruction and translation for training with different numbers of latent dimensions, using the dataset used for translation between PC3 and HA1E cell lines. The error bars denote a deviation of one Standard Error (SE) from the mean.

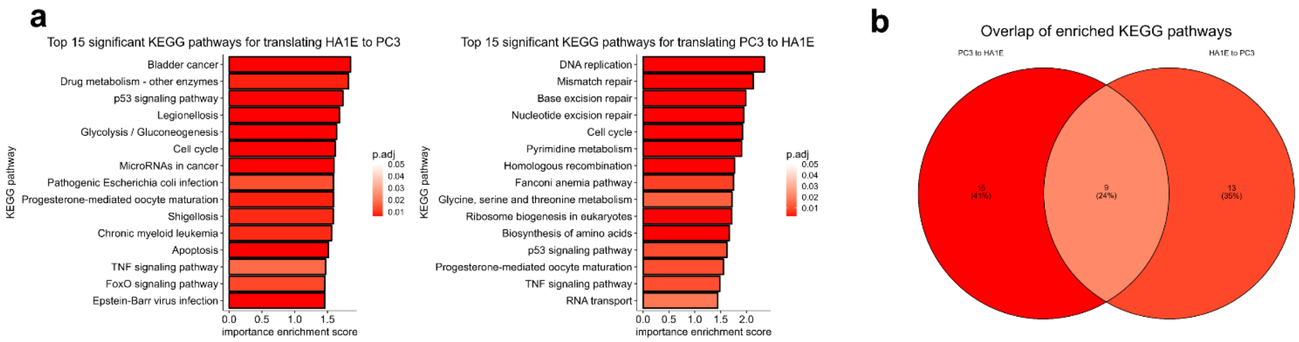

<u>Supplementary Figure 23:</u> a) Top significantly enriched KEGG pathways, based on importance scores of genes, to translate between cell lines. The enrichment means that a lot of the genes that belong to a pathway are found to be important, thus these KEGG pathways might be important for translating a cell line to another. **b)** Overlap of important KEGG pathways, derived from both directions of translation.

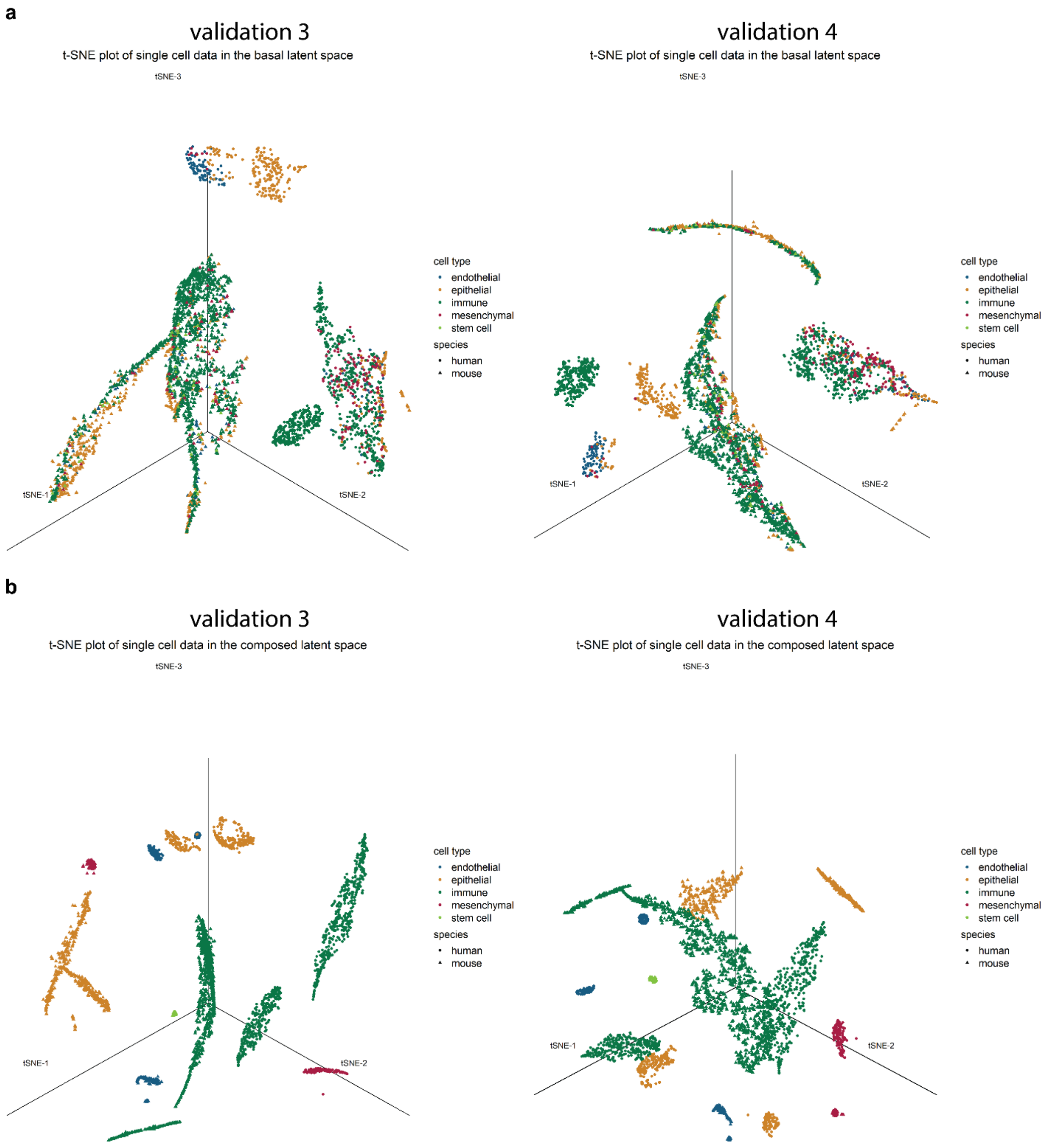

<u>Supplementary Figure 24:</u> The two splits in 10-fold cross-validation that are shown each time here are the ones where the maximum and minimum difference between the two distributions is observed. For every other split, the difference is between these two extreme cases. Latent space t-SNE visualization for embeddings derived from lung fibrosis datasets **a)** t-SNE plot of global latent space embeddings **b)** t-SNE plot of composed latent space embeddings

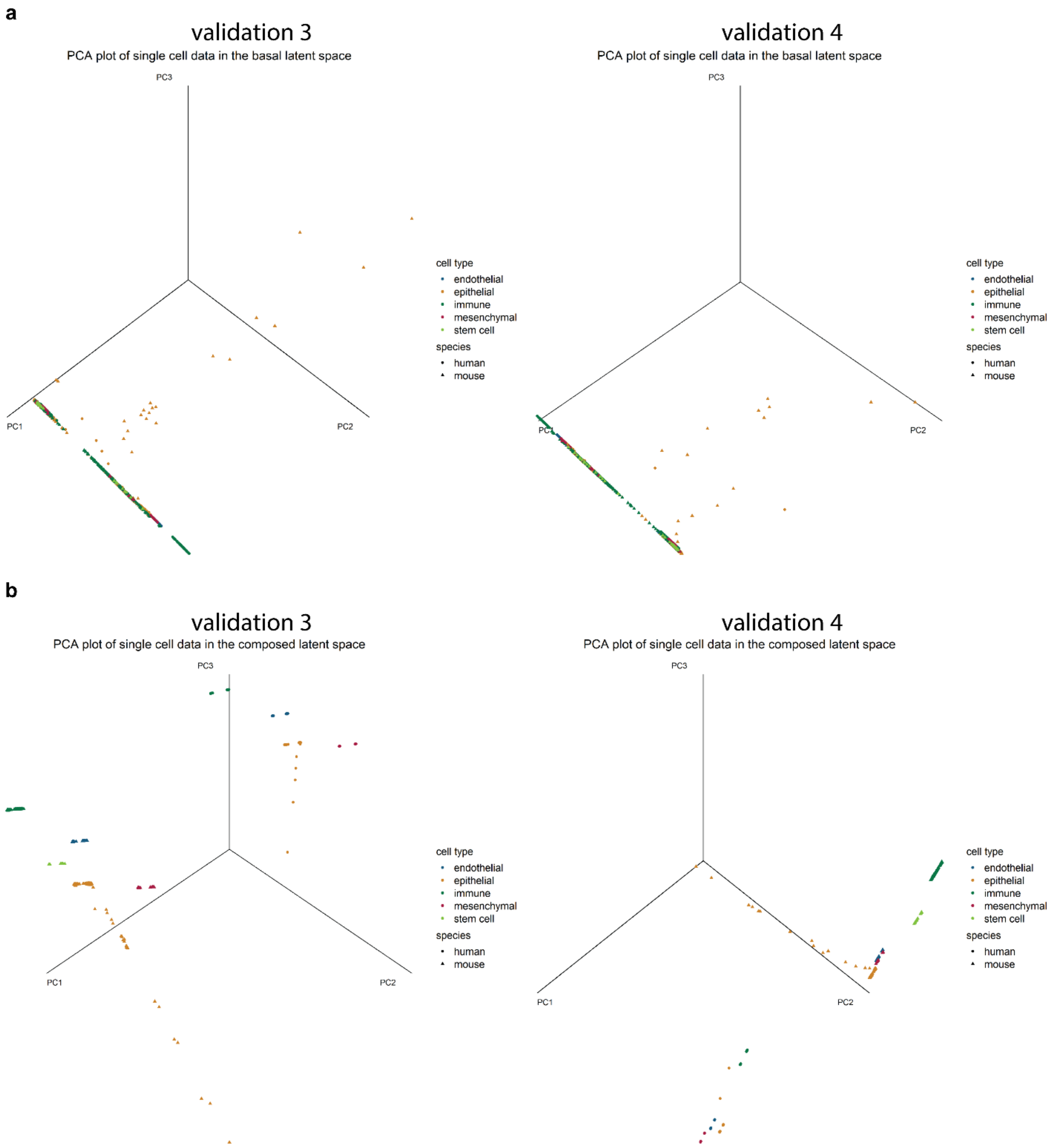

<u>Supplementary Figure 25:</u> The two splits in 10-fold cross-validation that are shown each time here are the ones where the maximum and minimum difference between the two distributions is observed. For every other split, the difference is between these two extreme cases. Latent space PCA visualization for embeddings derived from lung fibrosis datasets **a)** PCA plot of global latent space embeddings **b)** PCA plot of composed latent space embeddings

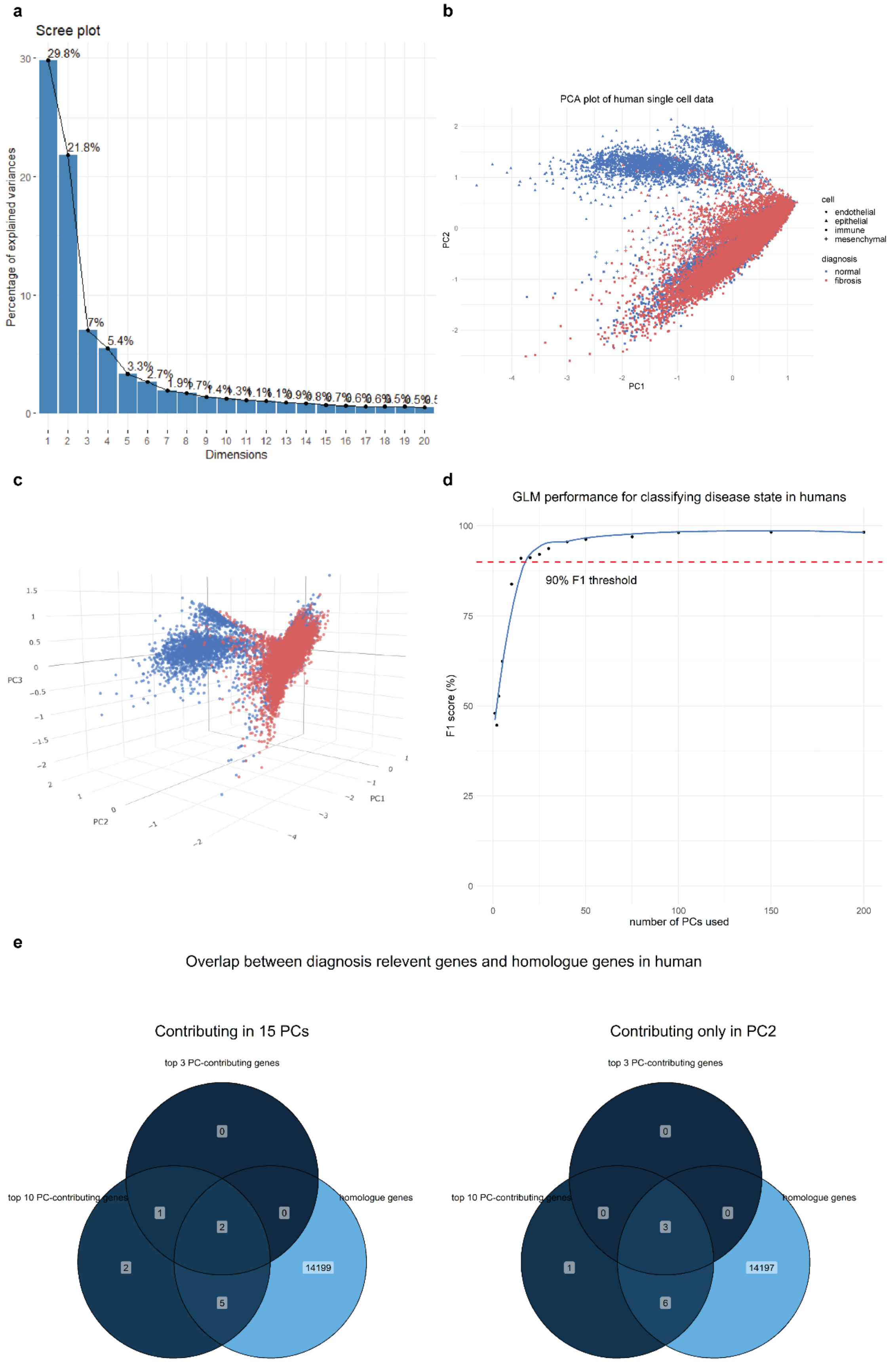

Supplementary Figure 26: Important genes analysis for human lung fibrosis **a)** Scree plot showing explained variance of each principal component (PC). The first 3 PCs capture 58.6% of the variance **b-c)**

2-D and 3-D visualization of gene expression data from the human lung fibrosis dataset. The first 3 PCs can separate samples based on fibrosis **d)** Performance in predicting fibrosis of a simple generalized linear model, using different numbers of PCs. Just with 15 PCs 90% F1 score is achieved and saturation of performance begins **e)** Overlap of important genes for human fibrosis, according to their loadings in first PCs, and homolog genes

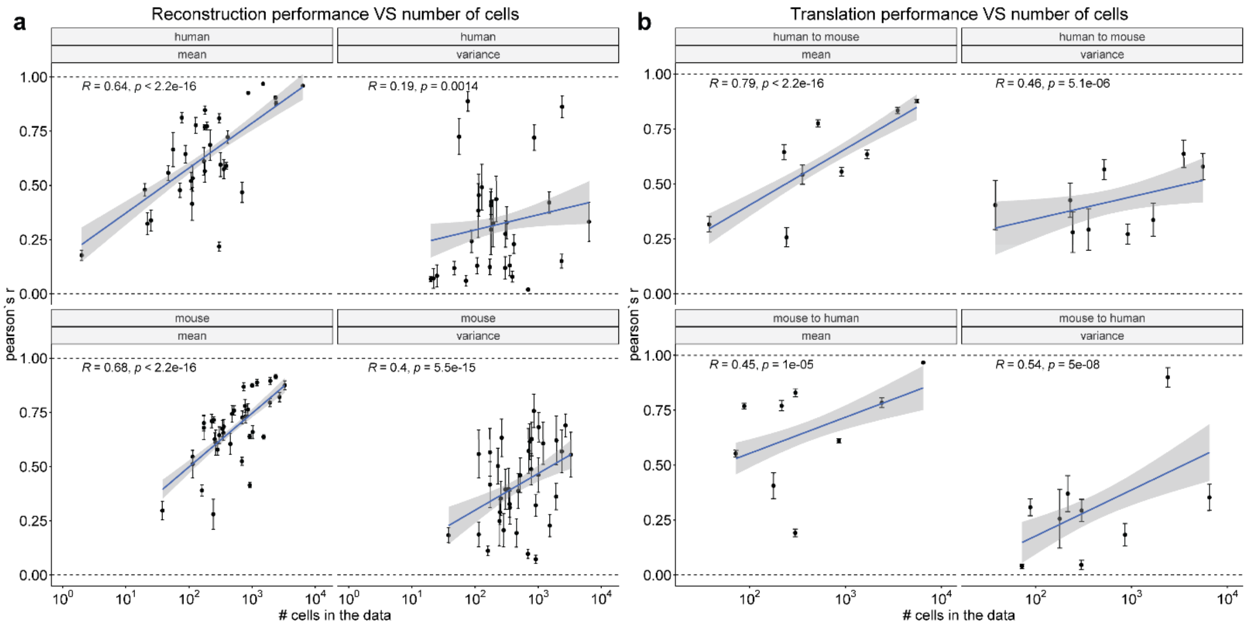

__**Supplementary Figure 27:**__ **a)** Performance in reconstructing the gene expression profile (in terms of per gene mean and variance) of each cell type, as a function of the number of cells of each cell type in the whole study **b)** Performance in translating the gene expression profile (in terms of per gene mean and variance) of each common cell type from one species to the other. The shaded areas denote 95% Confidence Intervals (CI) for predictions from a linear model, and the error bars denote a deviation of one Standard Error (SE) from the mean.

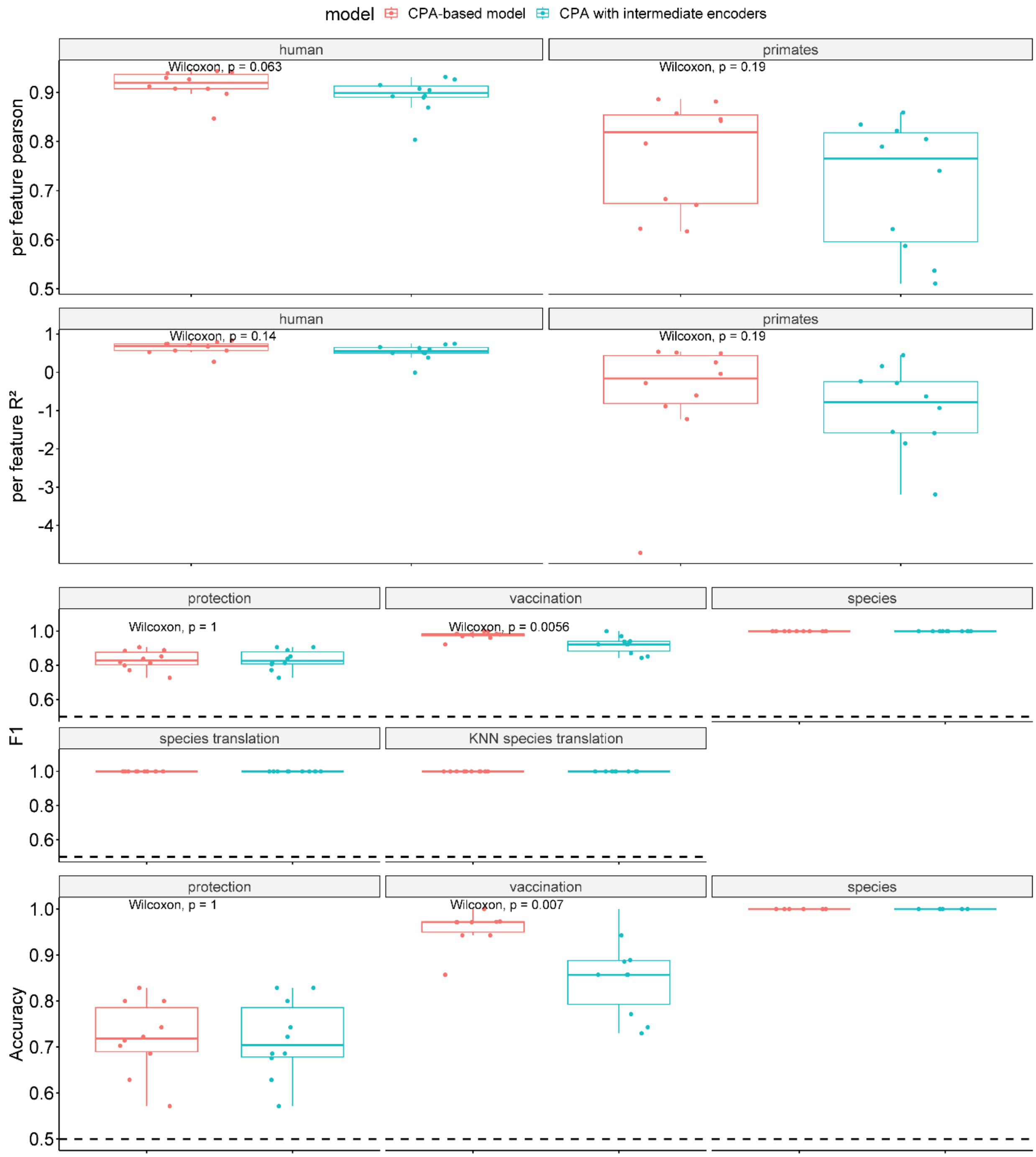

Supplementary Figure 28: Performance comparison between having a trainable vector or simple ANN to add cell line effect in the serology dataset. For all comparisons in this figure, a two-sided Wilcoxon test was used with n=10 per group. In all boxplots, the centerline denotes the median, the bounds of the box denote the 1st and 3rd quantiles, and the whiskers denote points not being further from the median than 1.5 x interquartile range (IQR).

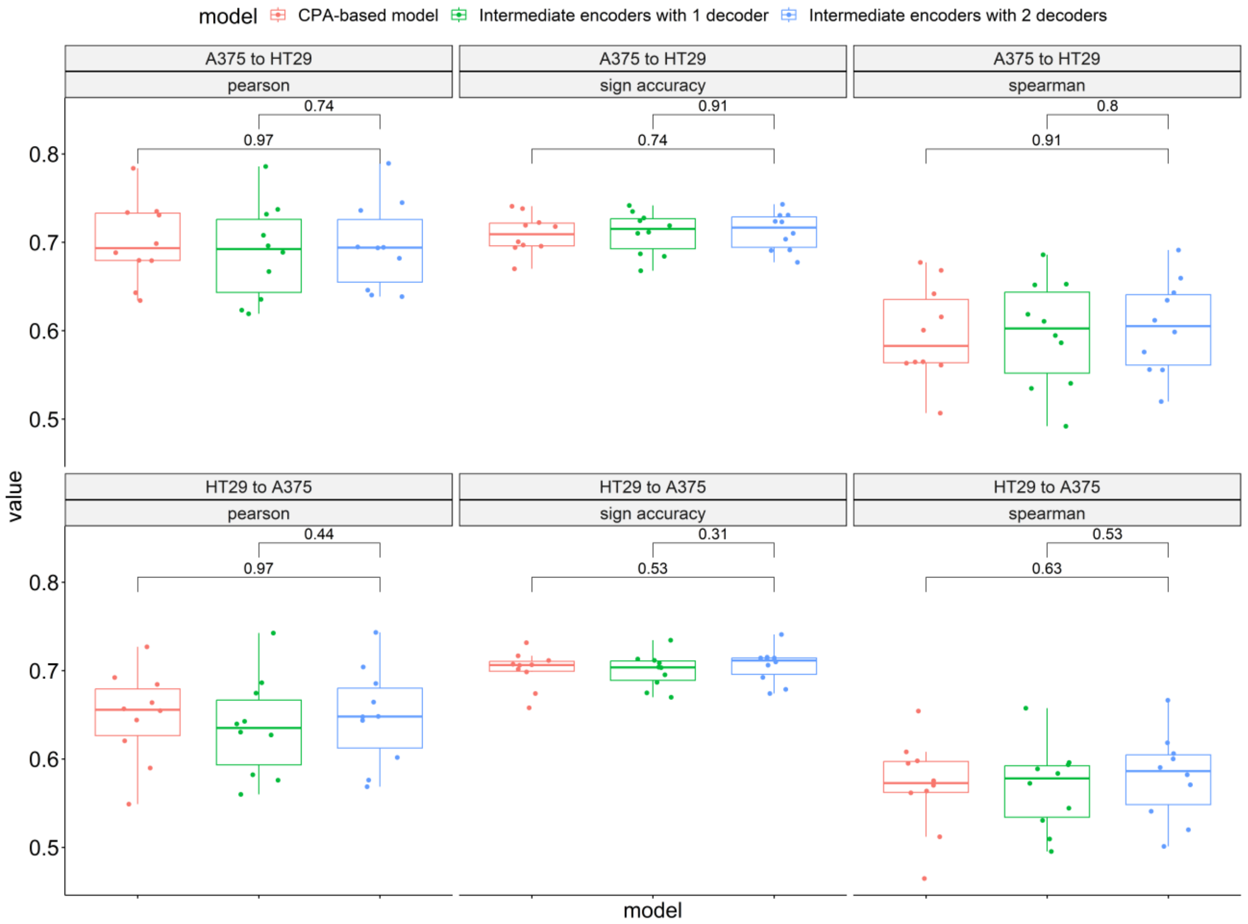

Supplementary Figure 29: Performance comparison between having a trainable vector or simple ANN to add cell line effect in the L1000 dataset. The comparison is done in the task of translating the gene expression profile of paired conditions. For all comparisons in this figure, a two-sided Wilcoxon test was used with n=10 per group. In all boxplots, the centerline denotes the median, the bounds of the box denote the 1st and 3rd quantiles, and the whiskers denote points not being further from the median than 1.5 x interquartile range (IQR).

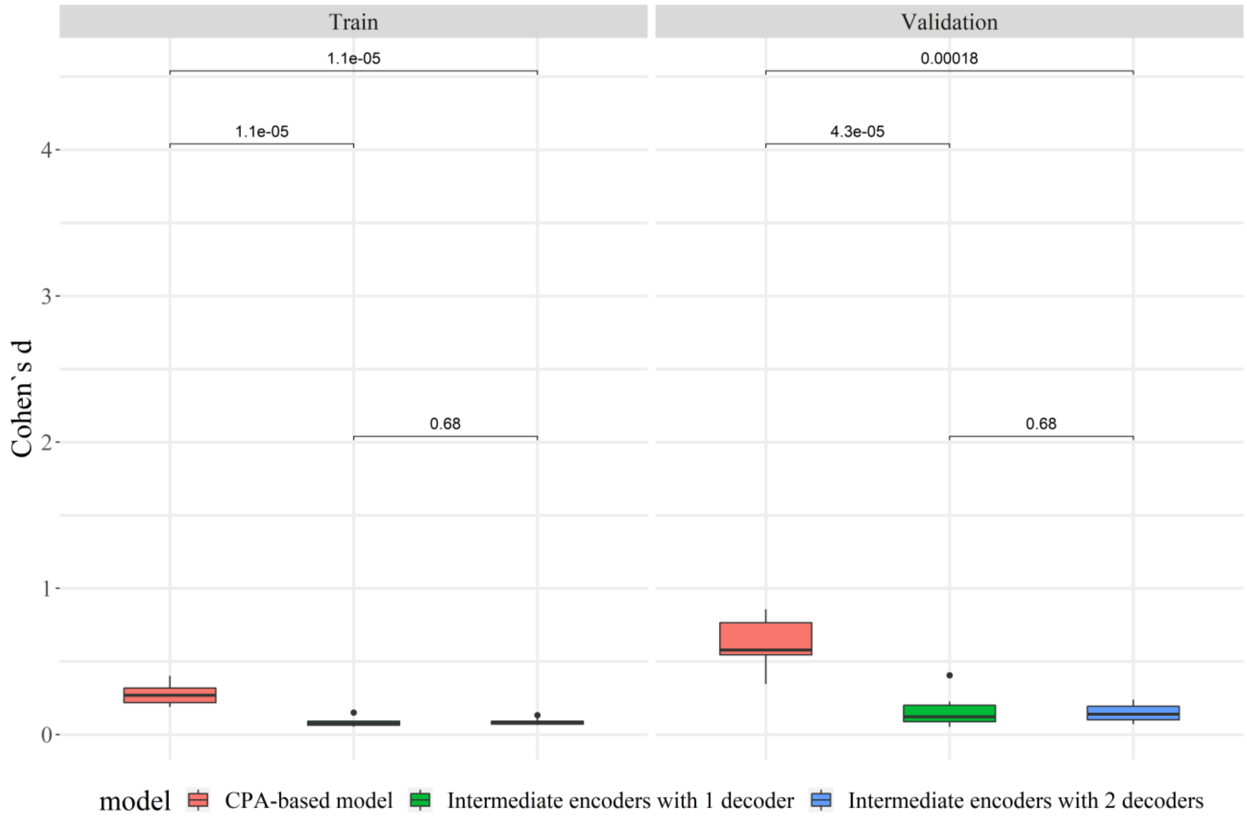

Supplementary Figure 30: Comparing Cohen's d between global and composed latent space when using a trainable vector or simple ANN to add cell line effect in the L1000 dataset. Using a trainable vector creates an even bigger difference between composed and basal latent space. For all comparisons in this figure, a two-sided Wilcoxon test was used with n=10 per group. In all boxplots, the centerline denotes the median, the bounds of the box denote the 1st and 3rd quantiles, and the whiskers denote points not being further from the median than 1.5 x interquartile range (IQR).

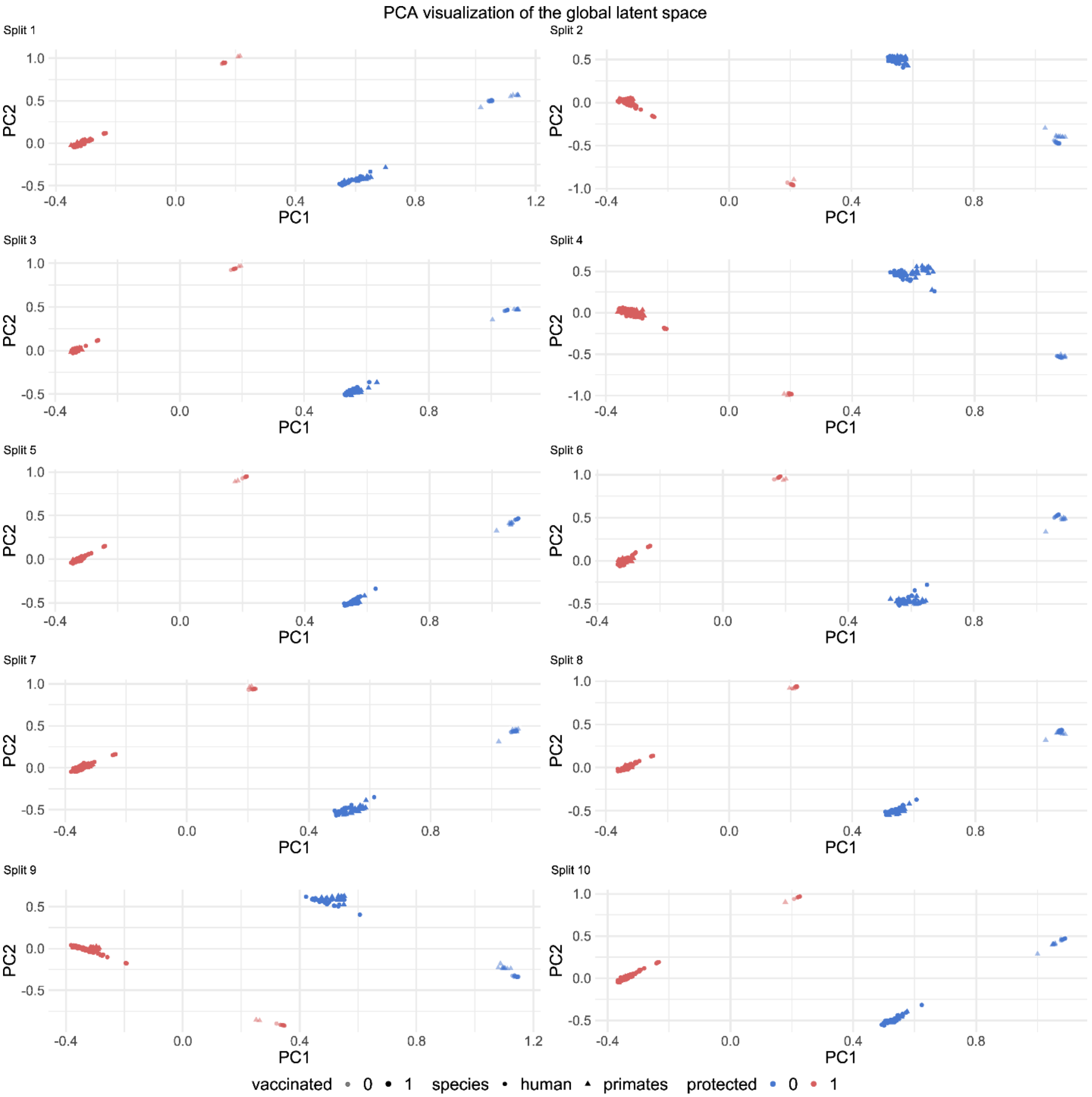

Supplementary Figure 31: Global Latent space PCA visualization for embeddings derived from the serology datasets. A clear separation based on protection and vaccination can be observed, while there is no separation based on species

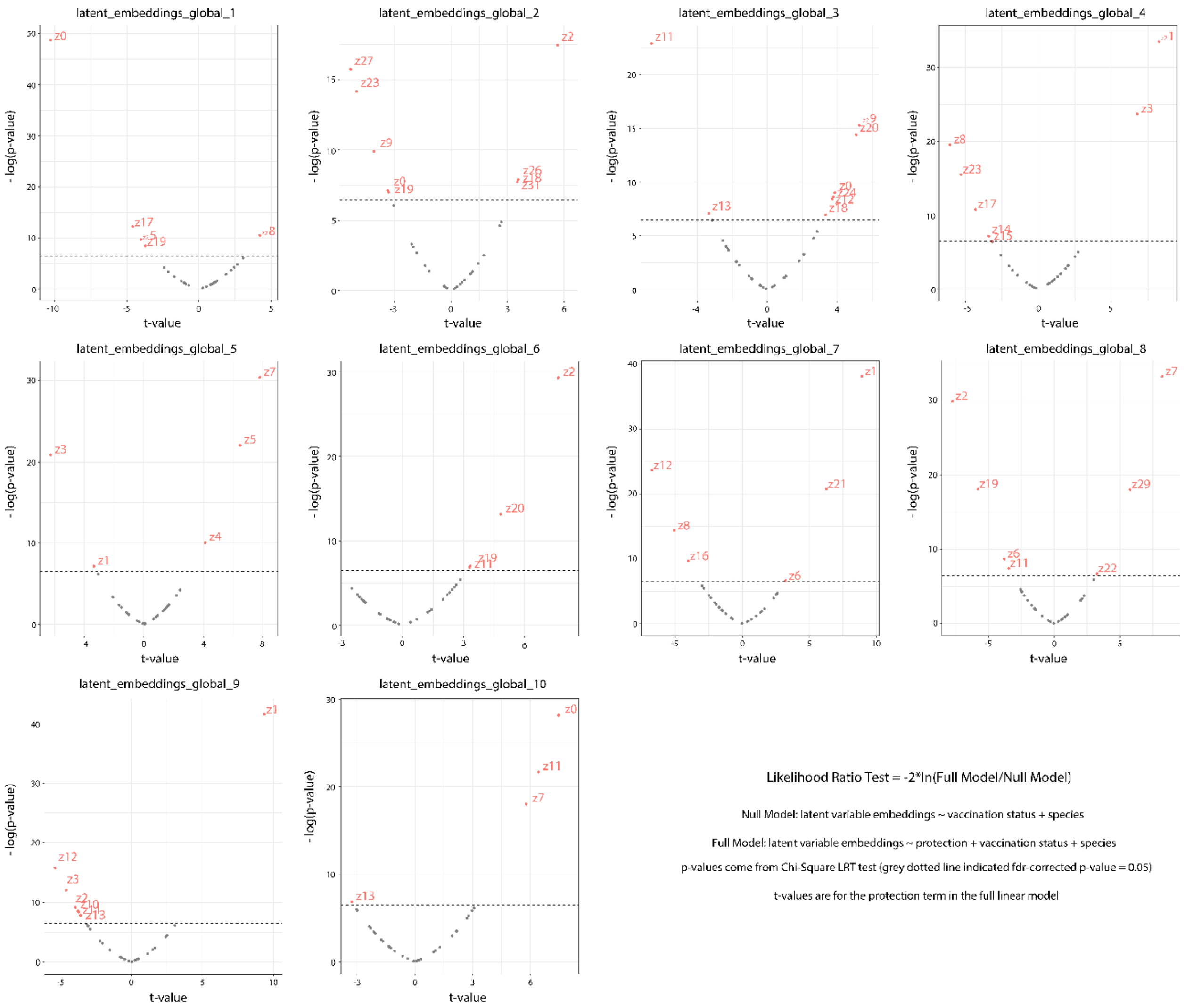

Supplementary Figure 32: Visualization of all likelihood ratio tests' results across folds, for identifying latent variables associated with viral protection in the serology case study.

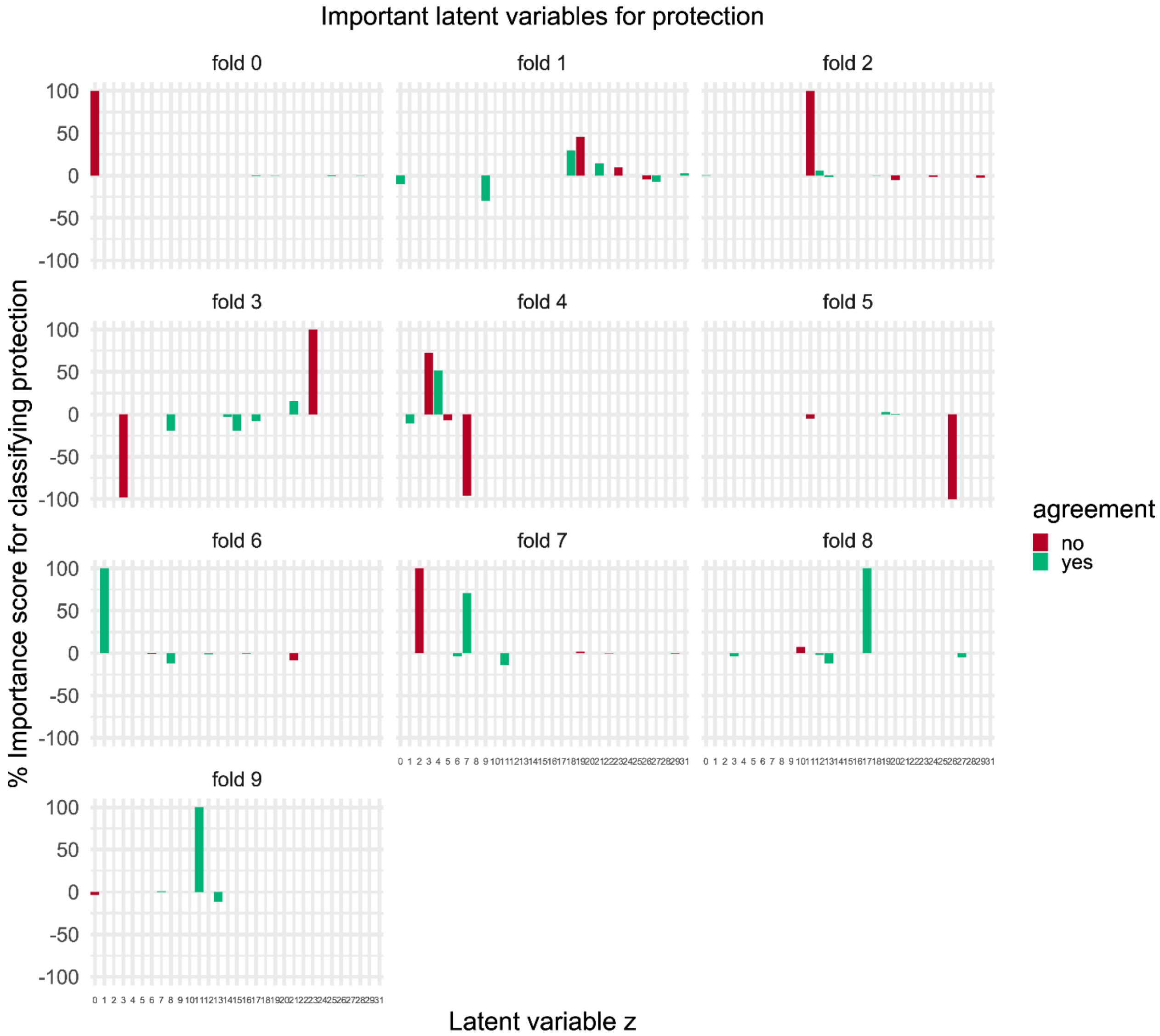

Supplementary Figure 33: Importance scores for viral protection of global latent variables, according to the classifier. Only values for statistically significant variables (according to the LRT test are shown). The agreement signifies the agreement in the sign of correlation with protection, between the classifier's score and the score from LRT.

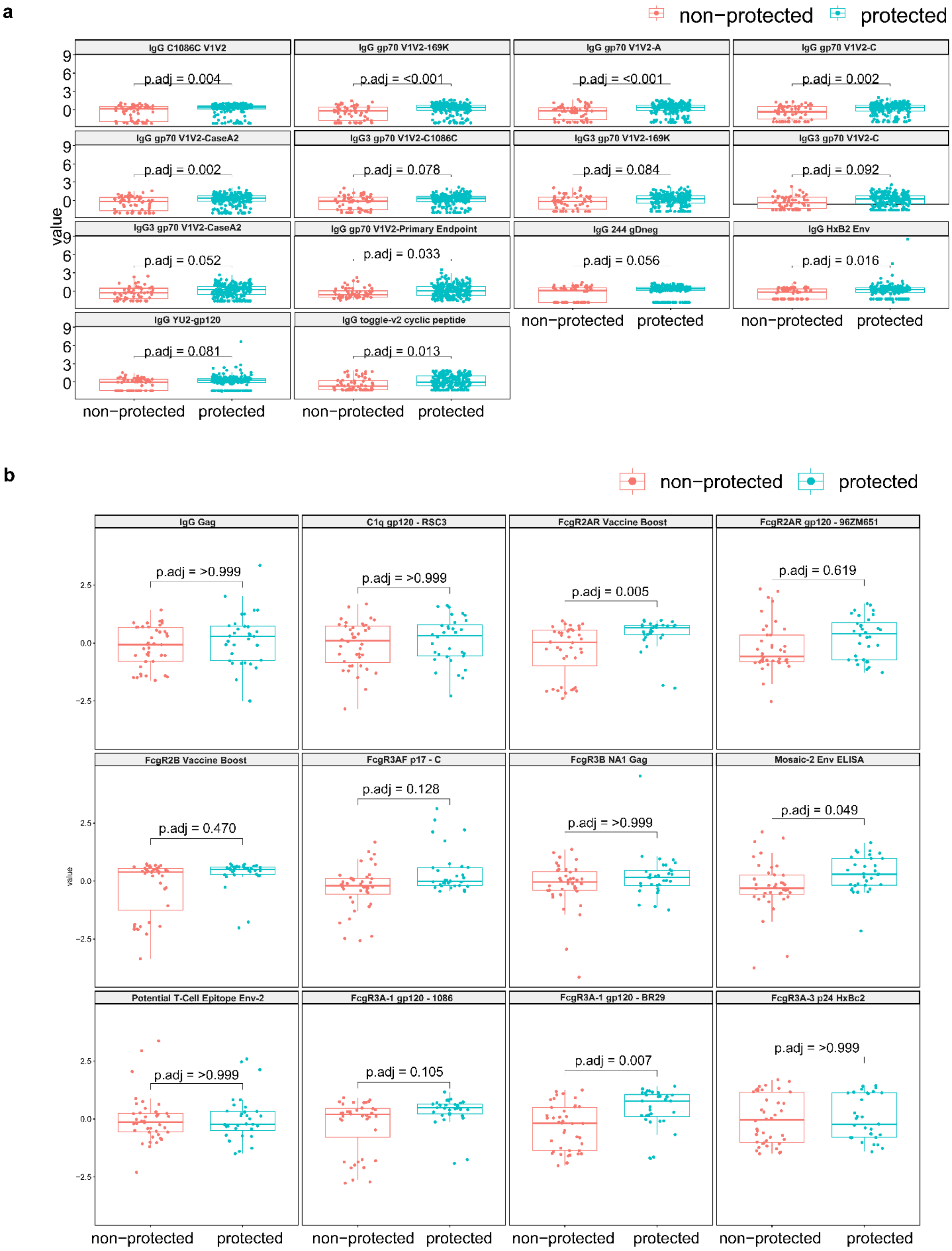

Supplementary Figure 34: **a)** Distributions of features between protected (n = 221) and non-protected (n = 60) humans. The features shown are those predictive of human viral protection. **b)** Distributions of features between protected (n = 32) and non-protected (n = 40) NHPs. The features shown are those predictive of human viral protection. A two-sided Wilcoxon test with Bonferroni correction was used. In

all boxplots, the centerline denotes the median, the bounds of the box denote the 1st and 3rd quantiles, and the whiskers denote points not being further from the median than 1.5 x interquartile range (IQR).

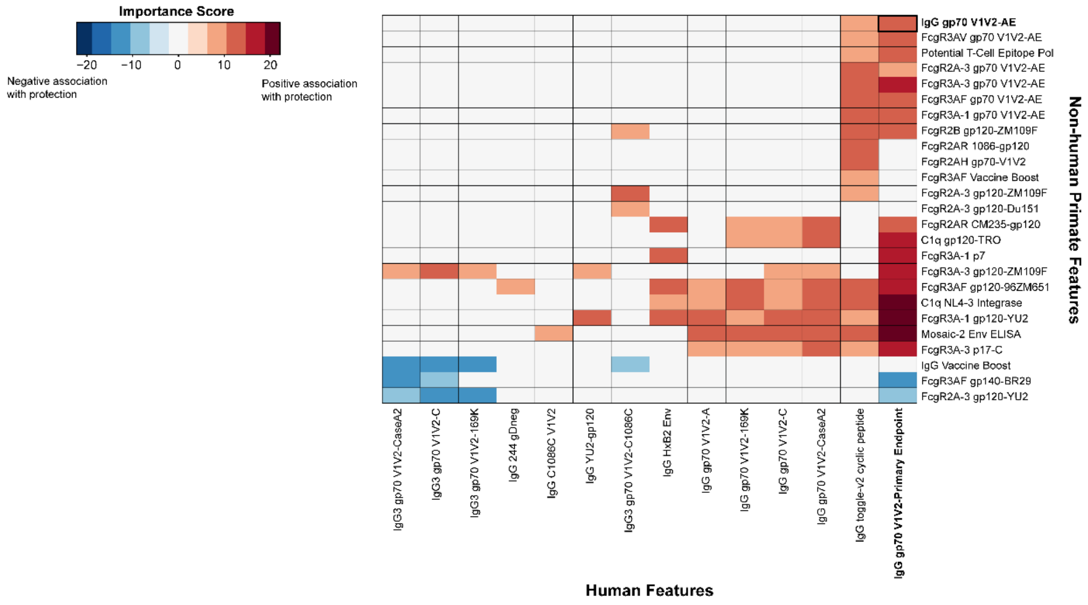

<u>Supplementary Figure 35:</u> Heatmap of non-human primate (NHP) features that have a consistently strong connection to protection-associated human features (see Methods for criteria). The features in bold fonts are the homologous features.

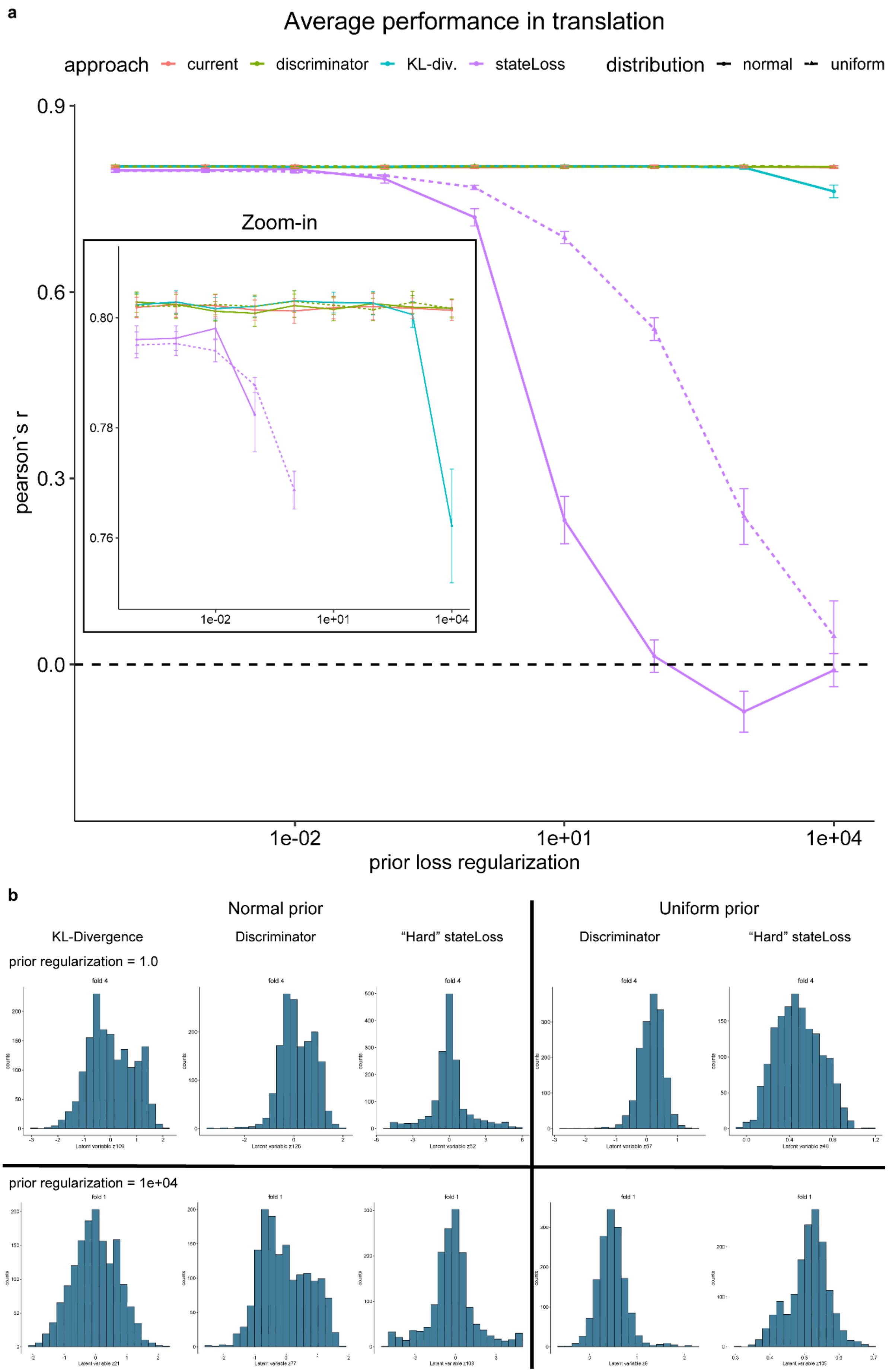
a
Average performance in translation
approach
current
discriminator
KL-div.
stateLoss
distribution
normal
uniform
0.9
0.6
0.3
0.0
pearson`s r
Zoom-in
0.80
0.78
0.76
1e-02
1e+01
1e+04
prior loss regularization
b
Normal prior
Uniform prior
KL-Divergence
Discriminator
"Hard" stateLoss
Discriminator
"Hard" stateLoss
prior regularization = 1.0
prior regularization = 1e+04

Supplementary Figure 36: **a)** Performance for different prior loss constraint functions and level of regularization. Error bars denote a deviation of one Standard Error (SE) from the mean. **b)** Distribution of latent embeddings after training with a low and a high level of regularization and different prior loss functions.

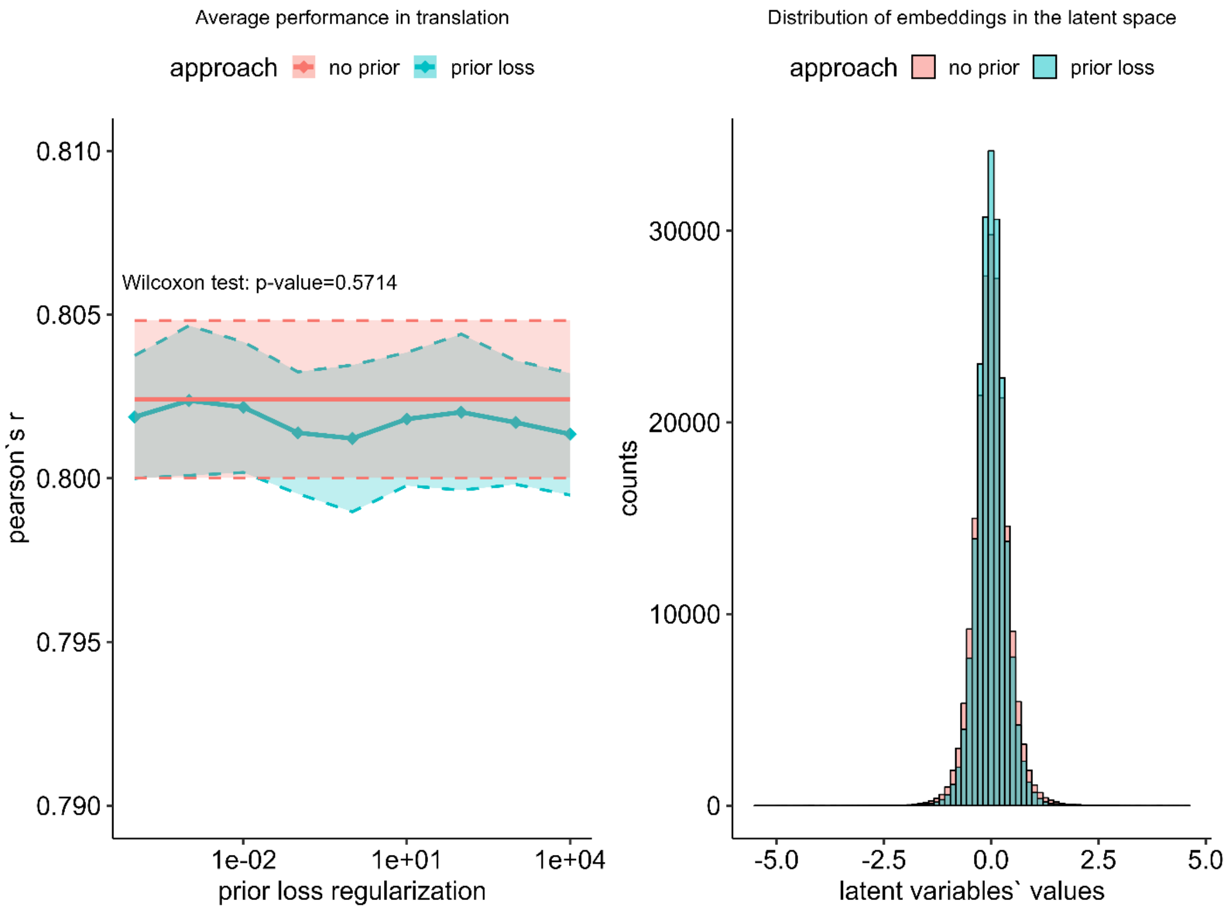

Supplementary Figure 37: **a)** Performance comparison between using a prior loss and not using the prior loss, for the translation task in the U2OS cell line. The shaded area represents a deviation of one Standard Error (SE) from the mean. **b)** Distribution of latent embeddings after training with or without a prior loss, for the U2OS cell line model.

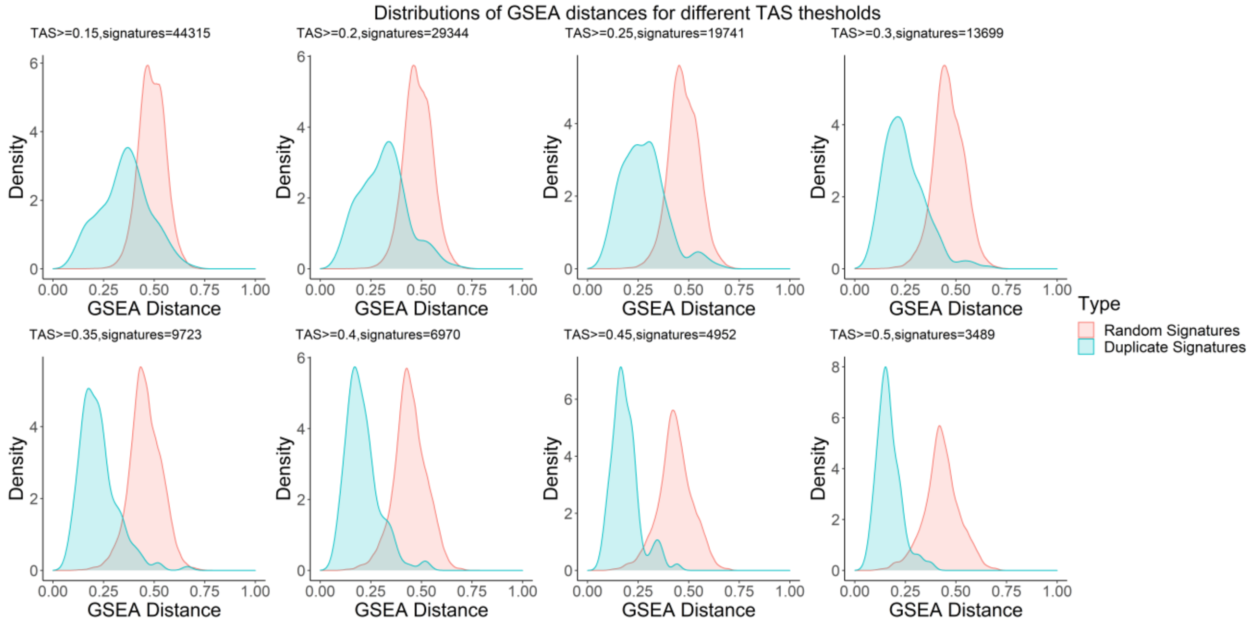

Supplementary Figure 38: Separation of GSEA-based distance (Supplementary Methods 5) distributions of gene expression between biological duplicates and randomly selected signatures for varying thresholds of Transcription Activity Score (TAS, see Main Methods), in the case of compound treatment data. There is a statistically significant separation for most TAS thresholds and a very strong separation after TAS>=0.3.

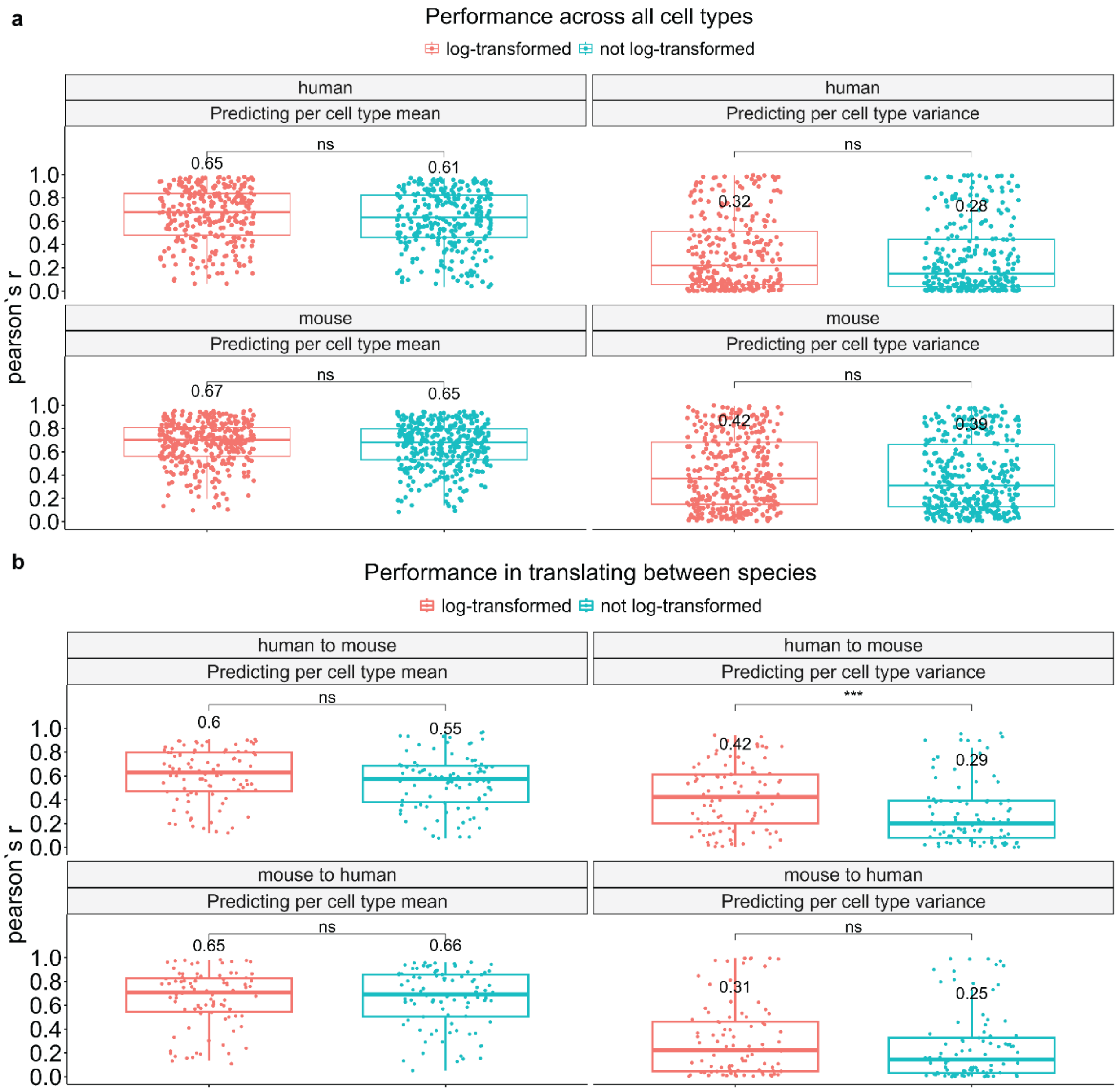

Supplementary Figure 39: Performance (Pearson's r) comparison when training the model with or without log-transformation of the raw data, in the single-cell RNA sequence of the human-mouse lung fibrosis datasets, to predict the per gene mean and variance of single-cell RNA-sequencing. **a)** Performance in the reconstruction task, across all cell types. **b)** Performance in the species translation task, across common cell types. In all boxplots, the centerline denotes the median, the bounds of the box denote the 1st and 3rd quantiles, and the whiskers denote points not being further from the median than 1.5 x interquartile range (IQR).

Supplementary notes

1. Pseudo-code of the training procedure of the model

Algorithm 1: Training of AutoTransOP v1 model

Input:

- $\boldsymbol{X_i}$, [bs x g] matrix for bs samples (same as the batch size bs) and g genes with each gene's expression under the specific condition of the sample.
- **conds$_i$,** [bs x 1] vector containing conditions' identifiers for each sample in the batch, to be used to identify similar conditions
- Enc_1, Enc_2, encoders of biological systems 1 and 2 respectively (e.g. cell lines, species, etc.), implemented as feedforward neural networks, that embed the gene expression into latent space representations ($\boldsymbol{Z_i}$)
- Dec_1, Dec_2, decoders of biological systems 1 and 2 respectively, implemented as feedforward neural networks, that reconstruct the original gene expression ($\boldsymbol{Y_i}$) from the latent space representations ($\boldsymbol{Z_i}$)
- $Discr_1$, the discriminator used to generate a similarity (probability) score between conditions in the batch. Similar conditions are denoted as p (from positives) and different conditions as n (from negatives)
- $Discr_2$, the discriminator used to calculate the probability that a latent representation is sampled from a uniform distribution

Output:

- $\boldsymbol{Z_i}$, [bs x d] matrix for bs samples (same as the batch size bs) and d dimensions, containing latent embedding representations of the input gene expression.
- $\boldsymbol{Y_i}$, [bs x g] matrix for bs samples and g genes containing the reconstructed gene expression.
- $\boldsymbol{loss}$, the value of the objective function used to train the model
- Updated weights for all parts of the model

for epochs **steps:**

    for batches **steps:**

        $\boldsymbol{X_1}, \boldsymbol{X_2} \leftarrow$ get data for the current batch *# get data for the batch*

        conds$_1$, conds$_2$ ← get conditions identifiers *# get*

        $\boldsymbol{Z_1} \leftarrow Enc_1(\boldsymbol{X_1})$ *# get latent representations*

        $\boldsymbol{Z_2} \leftarrow Enc_2(\boldsymbol{X_2})$

        $\boldsymbol{Y_1} \leftarrow Dec_1(\boldsymbol{Z_1})$ *# get reconstructed gene expression*

        $\boldsymbol{Y_2} \leftarrow Dec_2(\boldsymbol{Z_2})$

        # create mask of to identify positives (p) and negatives (n) in the batch

        $conditions \leftarrow$ concatenate$(\boldsymbol{conds_1}, \boldsymbol{conds_2})$

        $mak \leftarrow (conditions = conditions^T)$ # boolean matrix where 1 denotes similarity

        # $recon\_fun$ is the reconstruction loss function

        # different for single cell data and bulk data

        $\boldsymbol{L_{recon}} \leftarrow recon\_fun(\boldsymbol{X_1}, \boldsymbol{Y_1}) + recon\_fun(\boldsymbol{X_2}, \boldsymbol{Y_2})$

        # Calculate distance losses

        latent_vectors ← concatenate$(\boldsymbol{Z_1}, \boldsymbol{Z_2})$

        $\boldsymbol{L_{distance}} \leftarrow \frac{\sum elementwise\ multiplication(pairwise\ euclidean\ distance(\text{latent\_vectors}), mask)}{sum(mask)}$

        $w_1 \leftarrow L2\ norm\ across\ 2nd\ dimesnion(\boldsymbol{Z_1})$

$w_2 \leftarrow L2\ norm\ across\ 2nd\ dimesnion(\boldsymbol{Z_2})$

$$\boldsymbol{L_{cosine}} \leftarrow \frac{\sum elementwise\ multiplication\left(\frac{(\text{latent\_vectors} * latent\_vectors^T)}{w_1 * w_2^T}, mask\right)}{sum(mask)}$$

Calculate mutual information maximization loss

scores $\leftarrow Discr_1(\text{latent\_vectors})$

p $\leftarrow elementwise\ multiplication(scores, mask)$

n $\leftarrow elementwise\ multiplication(scores, 1 - mask)$

$E_p \leftarrow \ln(2) - softplus(-p)$

$E_q \leftarrow softplus(-n) + n - \ln(2)$

$E_p \leftarrow sum\left(elementwise\ multiplication(E_p, mask)\right) / sum(mask)$

$E_q \leftarrow sum\left(elementwise\ multiplication(E_q, 1 - mask)\right) / sum(1 - mask)$

$\boldsymbol{L_{MI}} \leftarrow -(E_p - E_q)$

Calculate prior loss

v = random uniform latent vectors

$\boldsymbol{L_{prior}} \leftarrow sum(\log(Discr_2(v)) + \log(1 - Discr_2(latent\_vectors))$

Calculate L2-regularization

$\boldsymbol{L_2} \leftarrow get\ L2\ regularization\ penalty\ of\ the\ model's\ weights$

Add to the whole loss

$\boldsymbol{loss} \leftarrow \lambda_{recon} * L_{recon} + \lambda_{distance} * L_{distance} + \lambda_{MI} * L_{MI} + \lambda_{prior} * L_{prior} - \lambda_{cosine} * L_{cosine} + L_2$

$\boldsymbol{calculate\ gradients\ with\ backpropagation\ in\ pytorch}: \boldsymbol{loss.backward}()$

$\boldsymbol{update\ weights\ with\ adam\ optimizer}: \boldsymbol{optimizer.step}()$

Supplementary Tables

Supplementary Table 1: P-values from comparing methods from Figure 1b in the task of translation. A two-sided Wilcoxon test was used to compare the performance of the different methods (n = 10 per group).

		A375									HT29								
		Autoencoders with classifier	CPA-based Autoencoders	DCS	DCS modified v1	DCS modified v2	FIT	TransCompR	direct translation	similarity-trained Autoencoders	Autoencoders with classifier	CPA-based Autoencoders	DCS	DCS modified v1	DCS modified v2	FIT	TransCompR	direct translation	similarity-trained Autoencoders
Autoencoders with classifier	pearson	1	0.6232				0.0013	0.3847			1	0.7337				$\leq 10^{-3}$	0.3075		
	sign accuracy	1	0.4727				$\leq 10^{-3}$	0.1212			1	0.6776				$\leq 10^{-3}$	0.1405		
	spearman	1	0.5205				$\leq 10^{-3}$	0.1859			1	0.6776				0.014	0.2123		
CPA-based Autoencoders	pearson		1				0.0017	0.6232				1							
	sign accuracy		1				$\leq 10^{-3}$	0.1212				1							
	spearman		1				$\leq 10^{-3}$	0.3075				1							
DCS	pearson	$\leq 10^{-3}$	$\leq 10^{-3}$	1	0.0036	$\leq 10^{-3}$	0.0539	$\leq 10^{-3}$	0.089	$\leq 10^{-3}$	$\leq 10^{-3}$	$\leq 10^{-3}$	1	0.0022	$\leq 10^{-3}$	0.0312	$\leq 10^{-3}$	0.0757	$\leq 10^{-3}$
	sign accuracy	$\leq 10^{-3}$	0.0013	1	0.0058	$\leq 10^{-3}$	$\leq 10^{-3}$	0.0013	0.064	$\leq 10^{-3}$	$\leq 10^{-3}$	$\leq 10^{-3}$	1	0.0036	$\leq 10^{-3}$	$\leq 10^{-3}$	0.001	0.0452	$\leq 10^{-3}$
	spearman	$\leq 10^{-3}$	0.0013	1	0.0036	$\leq 10^{-3}$	$\leq 10^{-3}$	0.0013	0.0376	$\leq 10^{-3}$	$\leq 10^{-3}$	$\leq 10^{-3}$	1	0.001	$\leq 10^{-3}$	$\leq 10^{-3}$	$\leq 10^{-3}$	0.0312	$\leq 10^{-3}$
DCS modified v1	pearson	0.1212	0.2413		1	0.2123	0.089	0.3847	$\leq 10^{-3}$	0.1212	0.0312	0.0376		1	0.0452	0.0376	0.1859	$\leq 10^{-3}$	0.0257
	sign accuracy	0.1041	0.2413		1	0.089	$\leq 10^{-3}$	0.9698	$\leq 10^{-3}$	0.0757	0.3075	0.273		1	0.1405	$\leq 10^{-3}$	0.9698	$\leq 10^{-3}$	0.1405
	spearman	0.2123	0.5205		1	0.1859	$\leq 10^{-3}$	1	$\leq 10^{-3}$	0.1859	0.2413	0.3847		1	0.1859	$\leq 10^{-3}$	0.9097	$\leq 10^{-3}$	0.2123
DCS modified v2	pearson	0.7337	0.9097			1	0.0013	0.5708	$\leq 10^{-3}$	0.7337	0.7337	0.9097			1	$\leq 10^{-3}$	0.3847	$\leq 10^{-3}$	0.6232
	sign accuracy	0.9698	0.5708			1	$\leq 10^{-3}$	0.0757	$\leq 10^{-3}$	0.9097	0.8501	0.5205			1	$\leq 10^{-3}$	0.0757	$\leq 10^{-3}$	1
	spearman	0.9097	0.3847			1	0.001	0.1041	$\leq 10^{-3}$	0.9097	0.7913	0.4727			1	0.0173	0.162	$\leq 10^{-3}$	0.8501
direct translation	pearson	$\leq 10^{-3}$	$\leq 10^{-3}$				0.001	$\leq 10^{-3}$	1		$\leq 10^{-3}$	$\leq 10^{-3}$				$\leq 10^{-3}$	$\leq 10^{-3}$	1	
	sign accuracy	$\leq 10^{-3}$	$\leq 10^{-3}$				$\leq 10^{-3}$	$\leq 10^{-3}$	1		$\leq 10^{-3}$	$\leq 10^{-3}$				$\leq 10^{-3}$	$\leq 10^{-3}$	1	
	spearman	$\leq 10^{-3}$	$\leq 10^{-3}$				$\leq 10^{-3}$	$\leq 10^{-3}$	1		$\leq 10^{-3}$	$\leq 10^{-3}$				$\leq 10^{-3}$	$\leq 10^{-3}$	1	
FIT	pearson						1	0.0046				$\leq 10^{-3}$				1	$\leq 10^{-3}$		
	sign accuracy						1	$\leq 10^{-3}$				$\leq 10^{-3}$				1	$\leq 10^{-3}$		
	spearman						1	$\leq 10^{-3}$				0.0058				1	$\leq 10^{-3}$		
similarity-trained Autoencoders	pearson	0.8501	0.6776				$\leq 10^{-3}$	0.273	$\leq 10^{-3}$	1	0.5205	0.4727				$\leq 10^{-3}$	0.2123	$\leq 10^{-3}$	1
	sign accuracy	1	0.4727				$\leq 10^{-3}$	0.0452	$\leq 10^{-3}$	1	0.9097	0.5708				$\leq 10^{-3}$	0.1212	$\leq 10^{-3}$	1
	spearman	0.9698	0.4727				$\leq 10^{-3}$	0.1212	$\leq 10^{-3}$	1	0.9097	0.4274				0.0257	0.162	$\leq 10^{-3}$	1
TransCompR	pearson							1				0.4274					1		
	sign accuracy							1				0.2413					1		
	spearman							1				0.4727					1		

Supplementary Table 2: P-values from comparing methods from Figure 1b in the task of reconstruction. A two-sided Wilcoxon test was used to compare the performance of the different methods (n = 10 per group).

		A375							HT29						
		Autoencoders with classifier	CPA-based Autoencoders	DCS	DCS modified v1	DCS modified v2	TransCompR	similarity-trained Autoencoders	Autoencoders with classifier	CPA-based Autoencoders	DCS	DCS modified v1	DCS modified v2	TransCompR	similarity-trained Autoencoders
Autoencoders with classifier	pearson	1	0.6232				0.3847		1	0.273				1	
	sign accuracy	1	0.2411				0.0022		1	0.4274				0.0376	
	spearman	1	0.2123				0.1859		1	0.3075				0.1212	
CPA-based Autoencoders	pearson		1							1					
	sign accuracy		1							1					
	spearman		1							1					
DCS	pearson	$\le 10^{-3}$	$\le 10^{-3}$	1	0.0036	$\le 10^{-3}$	$\le 10^{-3}$	$\le 10^{-3}$	$\le 10^{-3}$	$\le 10^{-3}$	1	$\le 10^{-3}$	$\le 10^{-3}$	$\le 10^{-3}$	$\le 10^{-3}$
	sign accuracy	$\le 10^{-3}$	$\le 10^{-3}$	1	$\le 10^{-3}$	$\le 10^{-3}$	$\le 10^{-3}$	$\le 10^{-3}$	$\le 10^{-3}$	$\le 10^{-3}$	1	$\le 10^{-3}$	$\le 10^{-3}$	$\le 10^{-3}$	$\le 10^{-3}$
	spearman	$\le 10^{-3}$	$\le 10^{-3}$	1	$\le 10^{-3}$	$\le 10^{-3}$	$\le 10^{-3}$	$\le 10^{-3}$	$\le 10^{-3}$	$\le 10^{-3}$	1	$\le 10^{-3}$	$\le 10^{-3}$	$\le 10^{-3}$	$\le 10^{-3}$
DCS modified v1	pearson	0.1212	0.2413		1	0.2123	0.3847	0.1212	0.0539	0.0036		1	$\le 10^{-3}$	0.064	0.0757
	sign accuracy	0.0113	0.0028		1	$\le 10^{-3}$	$\le 10^{-3}$	0.0173	0.273	0.0757		1	$\le 10^{-3}$	0.0058	0.273
	spearman	0.0017	$\le 10^{-3}$		1	$\le 10^{-3}$	$\le 10^{-3}$	0.0173	0.1041	0.0312		1	$\le 10^{-3}$	0.0113	0.064
DCS modified v2	pearson	0.7337	0.9097			1	0.5708	0.7337	$\le 10^{-3}$	$\le 10^{-3}$			1	$\le 10^{-3}$	$\le 10^{-3}$
	sign accuracy	$\le 10^{-3}$	$\le 10^{-3}$			1	$\le 10^{-3}$	$\le 10^{-3}$	$\le 10^{-3}$	$\le 10^{-3}$			1	$\le 10^{-3}$	$\le 10^{-3}$
	spearman	$\le 10^{-3}$	$\le 10^{-3}$			1	$\le 10^{-3}$	$\le 10^{-3}$	$\le 10^{-3}$	$\le 10^{-3}$			1	$\le 10^{-3}$	$\le 10^{-3}$
similarity-trained Autoencoders	pearson	0.8501	0.6776				0.273	1	0.7337	0.1859				0.6232	1
	sign accuracy	0.7337	0.4272				0.0036	1	0.7913	0.4274				0.0452	1
	spearman	0.9698	0.2413				0.162	1	0.9698	0.273				0.1405	1
TransCompR	pearson		0.6232				1			0.273				1	
	sign accuracy		0.0211				1			0.1405				1	
	spearman		0.9698				1			0.7913				1	

Supplementary Table 3: P-values from comparing methods from Figure 1c in the task of translation. A two-sided Wilcoxon test was used to compare the performance of the different methods (n = 10 per group).

		A375									HT29								
		Autoencoders with classifier	CPA-based Autoencoders	DCS	DCS modified v1	DCS modified v2	FIT	TransCompR	direct translation	similarity-trained Autoencoders	Autoencoders with classifier	CPA-based Autoencoders	DCS	DCS modified v1	DCS modified v2	FIT	TransCompR	direct translation	similarity-trained Autoencoders
Autoencoders with classifier	pearson	1	0.3447				0.0539	0.9698			1	0.5205				0.0028	0.6232		
	sign accuracy	1	0.273				$\le 10^{-3}$	0.8501			1	0.5205				$\le 10^{-3}$	0.5205		
	spearman	1	0.2123				$\le 10^{-3}$	0.9698			1	0.6232				$\le 10^{-3}$	0.6232		
CPA-based Autoencoders	pearson		1				0.1405	0.3847				1							
	sign accuracy		1				$\le 10^{-3}$	0.3447				1							
	spearman		1				$\le 10^{-3}$	0.3447				1							
DCS	pearson	0.273	0.6776	1	0.8501	0.0757	0.5205	0.3847	0.0312	0.3847	0.089	0.1859	1	0.9097	0.0211	0.5205	0.1405	0.0211	0.1859
	sign accuracy	0.3447	0.9698	1	0.9698	0.089	$\le 10^{-3}$	0.273	0.0257	0.6232	0.064	0.1859	1	0.9097	0.0539	$\le 10^{-3}$	0.1212	0.0173	0.3075
	spearman	0.4727	0.9698	1	1	0.089	$\le 10^{-3}$	0.3447	0.0257	0.6232	0.0757	0.273	1	0.9698	0.0539	$\le 10^{-3}$	0.1041	0.0113	0.2123
DCS modified v1	pearson	0.3447	0.8501		1	0.1041	0.5205	0.3847	0.0312	0.5205	0.089	0.1859		1	0.0376	0.4274	0.1859	0.0211	0.1859
	sign accuracy	0.3447	0.9698		1	0.089	$\le 10^{-3}$	0.273	0.0257	0.5708	0.064	0.273		1	0.0376	$\le 10^{-3}$	0.1405	0.0173	0.273
	spearman	0.3447	1		1	0.1041	$\le 10^{-3}$	0.3075	0.0257	0.6776	0.064	0.3075		1	0.0376	$\le 10^{-3}$	0.1405	0.0113	0.2123
DCS modified v2	pearson	0.4727	0.1041			1	0.0312	0.3075	0.0013	0.2413	0.4727	0.3075			1	0.0017	0.3075	$\le 10^{-3}$	0.4274
	sign accuracy	0.3075	0.1212			1	$\le 10^{-3}$	0.4727	0.0017	0.3447	0.5708	0.273			1	$\le 10^{-3}$	0.2413	$\le 10^{-3}$	0.3075
	spearman	0.273	0.1041			1	$\le 10^{-3}$	0.4274	0.001	0.2123	0.6776	0.3075			1	$\le 10^{-3}$	0.2413	$\le 10^{-3}$	0.273
direct translation	pearson	0.0022	0.0073				0.0312	0.0058	1		$\le 10^{-3}$	$\le 10^{-3}$				0.014	$\le 10^{-3}$	1	
	sign accuracy	0.0022	0.0073				$\le 10^{-3}$	0.0028	1		$\le 10^{-3}$	0.001				$\le 10^{-3}$	$\le 10^{-3}$	1	
	spearman	0.0022	0.0073				$\le 10^{-3}$	0.0022	1		$\le 10^{-3}$	$\le 10^{-3}$				$\le 10^{-3}$	$\le 10^{-3}$	1	
FIT	pearson						1	0.0757				0.0257				1	0.0091		
	sign accuracy						1	$\le 10^{-3}$				$\le 10^{-3}$				1	$\le 10^{-3}$		
	spearman						1	$\le 10^{-3}$				$\le 10^{-3}$				1	$\le 10^{-3}$		
similarity-trained Autoencoders	pearson	0.5205	0.6232				0.1041	0.7337	0.0036	1	0.5205	0.7913				0.0173	0.9097	$\le 10^{-3}$	1
	sign accuracy	0.5708	0.7913				$\le 10^{-3}$	0.5205	0.0046	1	0.5708	1				$\le 10^{-3}$	1	0.001	1
	spearman	0.5708	0.8501				$\le 10^{-3}$	0.7337	0.0036	1	0.6232	0.9698				$\le 10^{-3}$	0.9097	$\le 10^{-3}$	1
TransCompR	pearson							1				0.9698					1		
	sign accuracy							1				0.9698					1		
	spearman							1				0.7913					1		

Supplementary Table 4: P-values from comparing methods from Figure 1c in the task of reconstruction. A two-sided Wilcoxon test was used to compare the performance of the different methods (n = 10 per group).

		A375							HT29						
		Autoencoders with classifier	CPA-based Autoencoders	DCS	DCS modified v1	DCS modified v2	TransCompR	similarity-trained Autoencoders	Autoencoders with classifier	CPA-based Autoencoders	DCS	DCS modified v1	DCS modified v2	TransCompR	similarity-trained Autoencoders
Autoencoders with classifier	pearson	1	0.6776				$\leq 10^{-3}$		1	0.8501				0.0013	
	sign accuracy	1	0.6776				$\leq 10^{-3}$		1	0.7337				0.014	
	spearman	1	0.7337				$\leq 10^{-3}$		1	0.7913				0.0073	
CPA-based Autoencoders	pearson		1							1					
	sign accuracy		1							1					
	spearman		1							1					
DCS	pearson	$\leq 10^{-3}$	$\leq 10^{-3}$	1	$\leq 10^{-3}$	$\leq 10^{-3}$	$\leq 10^{-3}$	$\leq 10^{-3}$	$\leq 10^{-3}$	$\leq 10^{-3}$	1	$\leq 10^{-3}$	$\leq 10^{-3}$	$\leq 10^{-3}$	$\leq 10^{-3}$
	sign accuracy	$\leq 10^{-3}$	$\leq 10^{-3}$	1	$\leq 10^{-3}$	$\leq 10^{-3}$	$\leq 10^{-3}$	$\leq 10^{-3}$	$\leq 10^{-3}$	$\leq 10^{-3}$	1	$\leq 10^{-3}$	$\leq 10^{-3}$	$\leq 10^{-3}$	$\leq 10^{-3}$
	spearman	$\leq 10^{-3}$	$\leq 10^{-3}$	1	$\leq 10^{-3}$	$\leq 10^{-3}$	$\leq 10^{-3}$	$\leq 10^{-3}$	$\leq 10^{-3}$	$\leq 10^{-3}$	1	$\leq 10^{-3}$	$\leq 10^{-3}$	$\leq 10^{-3}$	$\leq 10^{-3}$
DCS modified v1	pearson	$\leq 10^{-3}$	$\leq 10^{-3}$		1	$\leq 10^{-3}$	$\leq 10^{-3}$	$\leq 10^{-3}$	$\leq 10^{-3}$	$\leq 10^{-3}$		1	$\leq 10^{-3}$	$\leq 10^{-3}$	$\leq 10^{-3}$
	sign accuracy	$\leq 10^{-3}$	$\leq 10^{-3}$		1	$\leq 10^{-3}$	$\leq 10^{-3}$	$\leq 10^{-3}$	$\leq 10^{-3}$	$\leq 10^{-3}$		1	$\leq 10^{-3}$	$\leq 10^{-3}$	$\leq 10^{-3}$
	spearman	$\leq 10^{-3}$	$\leq 10^{-3}$		1	$\leq 10^{-3}$	$\leq 10^{-3}$	$\leq 10^{-3}$	$\leq 10^{-3}$	$\leq 10^{-3}$		1	$\leq 10^{-3}$	$\leq 10^{-3}$	$\leq 10^{-3}$
DCS modified v2	pearson	$\leq 10^{-3}$	$\leq 10^{-3}$			1	$\leq 10^{-3}$	$\leq 10^{-3}$	$\leq 10^{-3}$	$\leq 10^{-3}$			1	$\leq 10^{-3}$	$\leq 10^{-3}$
	sign accuracy	$\leq 10^{-3}$	$\leq 10^{-3}$			1	$\leq 10^{-3}$	$\leq 10^{-3}$	$\leq 10^{-3}$	$\leq 10^{-3}$			1	$\leq 10^{-3}$	0.0017
	spearman	$\leq 10^{-3}$	$\leq 10^{-3}$			1	$\leq 10^{-3}$	$\leq 10^{-3}$	$\leq 10^{-3}$	$\leq 10^{-3}$			1	$\leq 10^{-3}$	$\leq 10^{-3}$
similarity-trained Autoencoders	pearson	0.7337	0.9097				$\leq 10^{-3}$	1	0.6776	0.9097				0.0073	1
	sign accuracy	0.7337	0.6776				$\leq 10^{-3}$	1	0.9097	0.7337				0.0312	1
	spearman	0.6776	0.5205				$\leq 10^{-3}$	1	0.7913	0.8501				0.014	1
TransCompR	pearson		$\leq 10^{-3}$				1			0.0058				1	
	sign accuracy		$\leq 10^{-3}$				1			0.0211				1	
	spearman		$\leq 10^{-3}$				1			0.0046				1	

Supplementary Table 5: Regularization λ terms in the loss function of different cases

Case	λ_{recon}	$\lambda_{distance}$	λ_{cosine}	λ_{MI}	λ_{prior}	$\lambda_{enc,i}$	$\lambda_{dec,i}$	$\lambda_{L2class,i}$	$\lambda_{class,i}$	$\lambda_{adverse}$	$\lambda_{trained_{effect}}$	$\lambda_{intermediate,enc}$
L1000 10k genes	1	1	40	100	1	10^{-2}	10^{-2}	10^{-2}	500	500	10^{-4}	10^{-5}
L1000 landmark genes	1	10	10	100	1	10^{-2}	10^{-2}	10^{-2}	1000	1000	10^{-4}	10^{-5}
Lung fibrosis	1	100	100	1	1	10^{-7}	10^{-7}	10^{-7}	1	10	10^{-6}	10^{-5}
Serology dataset	1	40	70	100	1	10^{-6}	10^{-6}	$10^{-4} - 10^{-5}$	100	100	10^{-4}	10^{-5}

Supplementary References

1. Subramanian, A. *et al.* A Next Generation Connectivity Map: L1000 Platform and the First 1,000,000 Profiles. *Cell* **171**, 1437-1452.e17 (2017).
2. Umarov, R., Li, Y. & Arner, E. DeepCellState: An autoencoder-based framework for predicting cell type specific transcriptional states induced by drug treatment. *PLOS Computational Biology* **17**, e1009465 (2021).

3. Brubaker, D. K. *et al.* An interspecies translation model implicates integrin signaling in infliximab-resistant inflammatory bowel disease. *Science Signaling* **13**, eaay3258 (2020).
