# Supplementary material for: AutoTransOP: translating omics signatures without orthologue requirements using deep learning
Source: NPJ Syst Biol Appl. 2024 Jan 29;10:13. doi: 10.1038/s41540-024-00341-9 (PMC10825146; doi:10.1038/s41540-024-00341-9)
Supplement: Supplementary file 2 — Reporting summary [file 41540_2024_341_MOESM2_ESM.pdf]

Corresponding author(s): Douglas A Lauffenburger
Last updated by author(s): 2024/01/15

Reporting Summary

Nature Portfolio wishes to improve the reproducibility of the work that we publish. This form provides structure for consistency and transparency in reporting. For further information on Nature Portfolio policies, see our Editorial Policies and the Editorial Policy Checklist.

Statistics

For all statistical analyses, confirm that the following items are present in the figure legend, table legend, main text, or Methods section.

n/a	Confirmed	
☐	☒	The exact sample size (*n*) for each experimental group/condition, given as a discrete number and unit of measurement
☒	☐	A statement on whether measurements were taken from distinct samples or whether the same sample was measured repeatedly
☐	☒	The statistical test(s) used AND whether they are one- or two-sided<br>*Only common tests should be described solely by name; describe more complex techniques in the Methods section.*
☒	☐	A description of all covariates tested
☐	☒	A description of any assumptions or corrections, such as tests of normality and adjustment for multiple comparisons
☒	☐	A full description of the statistical parameters including central tendency (e.g. means) or other basic estimates (e.g. regression coefficient) AND variation (e.g. standard deviation) or associated estimates of uncertainty (e.g. confidence intervals)
☐	☒	For null hypothesis testing, the test statistic (e.g. F, t, r) with confidence intervals, effect sizes, degrees of freedom and P value noted<br>*Give P values as exact values whenever suitable.*
☒	☐	For Bayesian analysis, information on the choice of priors and Markov chain Monte Carlo settings
☒	☐	For hierarchical and complex designs, identification of the appropriate level for tests and full reporting of outcomes
☐	☒	Estimates of effect sizes (e.g. Cohen's *d*, Pearson's *r*), indicating how they were calculated

Our web collection on statistics for biologists contains articles on many of the points above.

Software and code

Policy information about availability of computer code

Data collection	The study did not produce new experimental data. All data were retrieved from publicly available repositories (e.g. CMAP, L1000) manually, or retrieved from their respective publication for the case of published data. Links and information about the exact location of the publicly available data used is available in the following GitHub repository: https://github.com/Lauffenburger-Lab/OmicTranslationBenchmark (corresponding Zenodo: https://zenodo.org/doi/10.5281/zenodo.10475298).
Data analysis	All models were expressed in and trained using the PyTorch framework (version 1.12) in Python (version 3.8.8). Pre-processing and statistical analysis of the results were done in the R programming language (version 4.1.2). Visualization of results was done mainly using ggplot2, and figures were polished in Adobe Illustrator. More information about the versions of each library used and the exact code and scripts used, are available in the following GitHub repository: https://github.com/Lauffenburger-Lab/OmicTranslationBenchmark (corresponding Zenodo: https://zenodo.org/doi/10.5281/zenodo.10475298).

For manuscripts utilizing custom algorithms or software that are central to the research but not yet described in published literature, software must be made available to editors and reviewers. We strongly encourage code deposition in a community repository (e.g. GitHub). See the Nature Portfolio guidelines for submitting code & software for further information.

Data

Policy information about availability of data

All manuscripts must include a data availability statement. This statement should provide the following information, where applicable:

- Accession codes, unique identifiers, or web links for publicly available datasets
- A description of any restrictions on data availability
- For clinical datasets or third party data, please ensure that the statement adheres to our policy

The study did not produce any new experimental data. The L1000 dataset was accessed via clue.io. The single-cell RNA-sequence data from Strunz and Simon et al. and Habermann, Gutierrez, and Bui et al. can be found in the Gene Expression Omnibus under their respective accession numbers GSE141259 and GSE135893. The serology datasets were retrieved from Chung, Kumar, Arnold, et al., upon request, and Barouch, Tomaka, Wegmann, et al., upon request. All analyzed data that were used to train our models and produce all tables and figures are available at https://github.com/Lauffenburger-Lab/OmicTranslationBenchmark (corresponding Zenodo: https://zenodo.org/doi/10.5281/zenodo.10475298).

Research involving human participants, their data, or biological material

Policy information about studies with human participants or human data. See also policy information about sex, gender (identity/presentation), and sexual orientation and race, ethnicity and racism.

Reporting on sex and gender	*Use the terms sex (biological attribute) and gender (shaped by social and cultural circumstances) carefully in order to avoid confusing both terms. Indicate if findings apply to only one sex or gender; describe whether sex and gender were considered in study design; whether sex and/or gender was determined based on self-reporting or assigned and methods used. Provide in the source data disaggregated sex and gender data, where this information has been collected, and if consent has been obtained for sharing of individual-level data; provide overall numbers in this Reporting Summary. Please state if this information has not been collected. Report sex- and gender-based analyses where performed, justify reasons for lack of sex- and gender-based analysis.*
Reporting on race, ethnicity, or other socially relevant groupings	*Please specify the socially constructed or socially relevant categorization variable(s) used in your manuscript and explain why they were used. Please note that such variables should not be used as proxies for other socially constructed/relevant variables (for example, race or ethnicity should not be used as a proxy for socioeconomic status). Provide clear definitions of the relevant terms used, how they were provided (by the participants/respondents, the researchers, or third parties), and the method(s) used to classify people into the different categories (e.g. self-report, census or administrative data, social media data, etc.) Please provide details about how you controlled for confounding variables in your analyses.*
Population characteristics	*Describe the covariate-relevant population characteristics of the human research participants (e.g. age, genotypic information, past and current diagnosis and treatment categories). If you filled out the behavioural & social sciences study design questions and have nothing to add here, write "See above."*
Recruitment	*Describe how participants were recruited. Outline any potential self-selection bias or other biases that may be present and how these are likely to impact results.*
Ethics oversight	*Identify the organization(s) that approved the study protocol.*

Note that full information on the approval of the study protocol must also be provided in the manuscript.

Field-specific reporting

Please select the one below that is the best fit for your research. If you are not sure, read the appropriate sections before making your selection.

☒ Life sciences ☐ Behavioural & social sciences ☐ Ecological, evolutionary & environmental sciences

For a reference copy of the document with all sections, see nature.com/documents/nr-reporting-summary-flat.pdf

Life sciences study design

All studies must disclose on these points even when the disclosure is negative.

Sample size	No statistical method was used to predetermine sample size. Sample size was dictated by the already publicly available datasets. All the data not excluded (see below) were used to train deep learning models and perform downstream analysis.
Data exclusions	For the publicly available data of the serology and lung fibrosis case studies, all the available samples were used to train models and perform analysis. For the case of L1000 dataset, quality of each sample was estimated as described in the Methods section and data were filtered resulting in 13,699 samples coming from drug perturbations to train models. Briefly, data were filtered based on the similarity of the gene expression profiles of true biological duplicates (see manuscripts Methods for more details)
Replication	No new experimental data were generated or collected. No attempts were made to reproduce the already publicly available data.
Randomization	No new experimental data were generated or collected. Publicly available samples were divided for downstream analysis by disease or

Randomization	phenotype status (vaccinated, protected against infection, fibrosis diagnosis, species or cell type) or by the type of perturbation in the case of the L1000 (drug name, dose, time, cell line). No other randomization was performed.
Blinding	No new experimental data were generated or collected, so blinding does not apply for this study.

Reporting for specific materials, systems and methods

We require information from authors about some types of materials, experimental systems and methods used in many studies. Here, indicate whether each material, system or method listed is relevant to your study. If you are not sure if a list item applies to your research, read the appropriate section before selecting a response.

Materials & experimental systems

n/a	Involved in the study
☒	☐ Antibodies
☒	☐ Eukaryotic cell lines
☒	☐ Palaeontology and archaeology
☒	☐ Animals and other organisms
☒	☐ Clinical data
☒	☐ Dual use research of concern
☒	☐ Plants

Methods

n/a	Involved in the study
☒	☐ ChIP-seq
☒	☐ Flow cytometry
☒	☐ MRI-based neuroimaging

Plants

Seed stocks	*Report on the source of all seed stocks or other plant material used. If applicable, state the seed stock centre and catalogue number. If plant specimens were collected from the field, describe the collection location, date and sampling procedures.*
Novel plant genotypes	*Describe the methods by which all novel plant genotypes were produced. This includes those generated by transgenic approaches, gene editing, chemical/radiation-based mutagenesis and hybridization. For transgenic lines, describe the transformation method, the number of independent lines analyzed and the generation upon which experiments were performed. For gene-edited lines, describe the editor used, the endogenous sequence targeted for editing, the targeting guide RNA sequence (if applicable) and how the editor was applied.*
Authentication	*Describe any authentication procedures for each seed stock used or novel genotype generated. Describe any experiments used to assess the effect of a mutation and, where applicable, how potential secondary effects (e.g. second site T-DNA insertions, mosiacism, off-target gene editing) were examined.*
